# Management of Central Nervous System Infections, Vientiane, Laos, 2003–2011

**DOI:** 10.3201/eid2505.180914

**Published:** 2019-05

**Authors:** Audrey Dubot-Pérès, Mayfong Mayxay, Rattanaphone Phetsouvanh, Sue J. Lee, Sayaphet Rattanavong, Manivanh Vongsouvath, Viengmon Davong, Vilada Chansamouth, Koukeo Phommasone, Catrin Moore, Sabine Dittrich, Olay Lattana, Joy Sirisouk, Phonelavanh Phoumin, Phonepasith Panyanivong, Amphonesavanh Sengduangphachanh, Bountoy Sibounheuang, Anisone Chanthongthip, Manivone Simmalavong, Davanh Sengdatka, Amphaivanh Seubsanith, Valy Keoluangkot, Prasith Phimmasone, Kongkham Sisout, Khamsai Detleuxay, Khonesavanh Luangxay, Inpanh Phouangsouvanh, Scott B. Craig, Suhella M. Tulsiani, Mary-Anne Burns, David A.B. Dance, Stuart D. Blacksell, Xavier de Lamballerie, Paul N. Newton

**Affiliations:** Lao-Oxford-Mahosot Hospital-Wellcome Trust Research Unit, Mahosot Hospital, Vientiane, Laos (A. Dubot-Pérès, M. Mayxay, R. Phetsouvanh, S. Rattanavong, M. Vongsouvath, V. Davong, V. Chansamouth, K. Phommasone, C. Moore, S. Dittrich, O. Lattana, J. Sirisouk, P. Phoumin, P. Panyanivong, A. Sengduangphachanh, B. Sibounheuang, A. Chanthongthip, M. Simmalavong, D. Sengdatka, A. Seubsanith, D.A.B. Dance, P.N. Newton);; University of Oxford Nuffield Department of Clinical Medicine Center for Tropical Medicine and Global Health, Oxford, UK (A. Dubot-Pérès, S.J. Lee, C. Moore, S. Dittrich, D.A.B. Dance, S.D. Blacksell, P.N. Newton);; Unité des Virus Émergents (UVE: Aix-Marseille Univ-IRD 190-INSERM 1207-IHU Méditerranée Infection), Marseille, France (A. Dubot-Pérès, X. de Lamballerie);; University of Health Sciences Institute of Research and Education Development, Vientiane (M. Mayxay);; Mahidol University Faculty of Tropical Medicine Mahidol– Oxford Tropical Medicine Research Unit, Bangkok, Thailand (S.J. Lee, S.D. Blacksell);; Mahosot Hospital, Vientiane (V. Keoluangkot, P. Phimmasone, K. Sisout, K. Detleuxay, K. Luangxay, I. Phouangsouvanh);; Queensland Health Forensic and Scientific Service World Health Organization Collaborating Centre for Reference and Research on Leptospirosis, Brisbane, Queensland, Australia (S.B. Craig, S.M. Tulsiani, M.-A. Burns);; London School of Hygiene and Tropical Medicine Faculty of Infectious and Tropical Diseases, London, UK (D.A.B. Dance, P.N. Newton)

**Keywords:** central nervous system infections, patient care management, meningitis, encephalitis, meningitis/encephalitis, bacterial infections, viral infections, Laos, Lao, Asia, bacteria, viruses, Japanese encephalitis virus, *Orientia tsutsugamushi*, *Leptospira*, *Rickettsia*, *Cryptococcus*, antibiotics, antimicrobial medicines, diabetes, mortality, WHO meningitis, WHO encephalitis

## Abstract

During 2003–2011, we recruited 1,065 patients of all ages admitted to Mahosot Hospital (Vientiane, Laos) with suspected central nervous system (CNS) infection. Etiologies were laboratory confirmed for 42.3% of patients, who mostly had infections with emerging pathogens: viruses in 16.2% (mainly Japanese encephalitis virus [8.8%]); bacteria in 16.4% (including *Orientia tsutsugamushi* [2.9%], *Leptospira* spp. [2.3%], and *Rickettsia* spp. [2.3%]); and *Cryptococcus* spp. fungi in 6.6%. We observed no significant differences in distribution of clinical encephalitis and meningitis by bacterial or viral etiology. However, patients with bacterial CNS infection were more likely to have a history of diabetes than others. Death (26.3%) was associated with low Glasgow Coma Scale score, and the mortality rate was higher for patients with bacterial than viral infections. No clinical or laboratory variables could guide antibiotic selection. We conclude that high-dependency units and first-line treatment with ceftriaxone and doxycycline for suspected CNS infections could improve patient survival in Laos.

Central nervous system (CNS) infections, which can be caused by a number of different viruses, bacteria, fungi, and parasites, cause substantial disease and death in Southeast Asia (*1*). The etiologies of these infections are usually confirmed in <50% patients globally (*2*,*3*). Conventionally, most CNS infections are classified as meningitis or encephalitis by using a diverse set of clinical and laboratory definitions. The main causes of meningitis reported in Asia are *Mycobacterium tuberculosis*, *Streptococcus pneumoniae*, *Streptococcus suis*, *Neisseria meningitidis*, and *Cryptococcus* spp. (Appendix Table 1). Physicians rarely consider rickettsial and leptospiral pathogens, but interest in these reemerging treatable etiologies is resurfacing (*4*). Emerging viruses are important causes of CNS infections in Asia. Japanese encephalitis virus (JEV) causes ≈68,000 cases of encephalitis a year (*5*), and dengue virus is increasingly reported as a cause of neurologic disease, occurring in 0.5%–6.2% of dengue patients (*6*–*9*). Other common viral causes of encephalitis include enterovirus and herpes simplex viruses (HSVs) 1 and 2 (*10*).

Few data globally are available to guide policy on the prevention, diagnosis, and treatment of CNS infections, and the diversity of definitions for different CNS infection syndromes is confusing (*11*); some case definitions use clinical criteria only (*12*,*13*), and others include additional laboratory variables (*10*,*14*). Meningitis (i.e., meningeal infection) and encephalitis (i.e., parenchymal infection) presumably represent a continuum, but the diversity of clinical and laboratory features and etiologies across this wide spectrum is poorly understood. The standard for differentiating encephalitis from meningitis is histopathology, but biopsies and autopsies are rarely performed in Asia.

In Laos, the only comprehensive routine cerebrospinal fluid (CSF) diagnostic service available is in the capital city, Vientiane, at Mahosot Hospital (*15*–*17*). After a publication reporting rickettsial and leptospiral pathogens as important causes of CNS infections in Laos (*4*), we present the results of the full investigation conducted on the causes of CNS infection in this hospital to guide public health policy and treatment guidelines.

## Methods

### Study Site and Patient Recruitment

This study was prospectively conducted (January 2003–August 2011) with inpatients on all wards of Mahosot Hospital in Vientiane (17.959431°N, 102.613144°E, 188 m above mean sea level), an ≈400-bed hospital providing primary, secondary, and tertiary care and admitting ≈2,000 patients/month. We recruited inpatients of all ages for whom diagnostic lumbar puncture was indicated for suspicion of CNS infection because of altered consciousness or neurologic findings and for whom lumbar puncture was not contraindicated. For patient inclusion, we used no formal definition for CNS infection; patient recruitment was at the discretion of the responsible physician, reflecting local clinical practice. We recorded patient history and examination findings on standardized forms.

### Ethics Statement

We obtained verbal (2003–2006) or written (2006–2011) informed consent from all recruited patients or close relatives. Ethics clearance was granted by the Ethical Review Committee of the Faculty of Medical Sciences, National University of Laos (Vientiane, Laos), and the Oxford University Tropical Ethics Research Committee (Oxford, UK).

### Encephalitis and Meningitis Clinical Case Definitions

**Figure 1:**
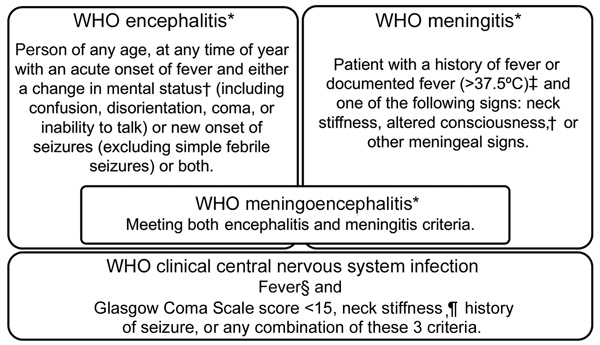
WHO encephalitis and meningitis case definitions. *Definitions from WHO (*18*). †Defined here as Glasgow Coma Scale score <15. ‡Not “with sudden onset of fever >38.5°C” as recommended by the WHO because we saw patients, especially young children, with meningitis but with temperatures below the WHO temperature criterion. §Patients with history of fever or documented fever (>37.5°C). ¶History of neck stiffness or neck stiffness on examination. WHO, World Health Organization.

We classified febrile patients meeting the World Health Organization (WHO) criteria for encephalitis or meningitis (Figure 1) (*18*) as patients with WHO clinical CNS (hereafter WHO CNS) infection. Because of the overlapping WHO case definitions for encephalitis and meningitis, which both include a Glasgow Coma Scale (GCS) score <15 as criteria, we created additional classifications for febrile patients: those with stiff neck; reduced GCS score (<15), seizures, or both; stiff neck and reduced GCS score, seizures, or both; no stiff neck but reduced GCS score, seizures, or both; and stiff neck, a GCS score of 15, and no seizures (Table 1).

**Table 1:** Classifications of febrile patients with clinical central nervous system infection (n = 771), Laos, January 2003–August 2011*

Clinical sign			No. (%) patients					Additional classification, %				
								Stiff neck	GCS score <15, seizure, or both			Stiff neck + GCS score 15 + no seizure
Stiff neck	GCS score <15	Seizure		WHO classification, %†					All	Stiff neck	No stiff neck	
				MEN	E	ME						
+			191 (24.8)	96.2				83.5				24.8
+	+		244 (31.6)		75.2	71.5			75.2	58.8		
+	+	+	171 (22.2)									
+		+	38 (4.9)									
	+	+	57 (7.4)								16.5	
	+		41 (5.3)									
		+	29 (3.8)									

*Each line of the table corresponds to a given combination of criteria presented on the left side of the table. Each combination is then assigned (shaded cell) to >1 classifications (WHO and additional classifications) as defined in the Methods section. Green-shaded cells are the additional classifications used throughout our study. E, encephalitis; GCS, Glasgow Coma Scale; ME, meningoencephalitis; MEN, meningitis; WHO, World Health Organization; +, clinical sign present. †Definitions of WHO classification provided in Figure 1.

### Laboratory Tests

Cerebrospinal fluid (CSF) was collected from patients (≈8 mL for adults [defined as patients >15 years of age], ≈3.5 mL for children 1–14 years of age, and ≈2.5 mL for children <1 year of age), and opening pressure was recorded. Venous blood (≈18.5 mL for adults, 10 mL for children 1–14 years of age, and 5.5 mL for children <1 year of age) was drawn on the same day. We aimed to collect ≈2 mL follow-up serum 7–10 days after lumbar puncture. Specimens were transported to the laboratory within ≈30 minutes, and we aliquoted and immediately tested or stored them at −80°C. We submitted all patient samples for a panel of laboratory tests: complete blood count; biochemistry panel; culture; and serologic and molecular assays for a range of bacteria, viruses, parasites, and fungi (Table 2; Appendix). HIV-1 and HIV-2 rapid diagnostic tests were performed when indicated by the physician. Computed tomography brain scan was available starting in 2002 but rarely used, especially for intensive care patients, because of difficulties transferring patients. Magnetic resonance imaging and electroencephalographic facilities were not available.

**Table 2:** Diagnostic laboratory tests used to confirm etiology in patients with suspected central nervous system infection, by sample type, Laos, January 2003–August 2011*

Pathogens tested	Cerebrospinal fluid†	Blood
Malaria pathogens	None	Giemsa thick and thin smear (if patient from endemic area)
*Leptospira* spp.	Hydrolysis probe real-time PCR (*19*)	Culturing of blood clot on EMJH medium; microscopic agglutination test of admission and follow-up serum samples (4-fold rise considered positive result) (*20*); hydrolysis probe real-time RT-PCR from buffy coat (*19*)
*Cryptococcus* spp.	Indian ink stain; if HIV suspected, *Cryptococcus* Antigen Latex Agglutination Test (IMMY, http://www.immy.com); if Indian ink positive and HIV suspected, culture on Sabouraud agar	None
*Mycobacterium tuberculosis*	Lowenstein-Jensen culture; auramine and Ziehl-Neelsen stains	None
*Rickettsia* spp.‡	Hydrolysis probe real-time PCR (*21**,**22*)	Hydrolysis probe real-time and conventional PCR from buffy coat (*21**,**22*); sequencing
*R. typhi*, *Orientia tsutsugamushi*‡	Hydrolysis probe real-time PCR (*21**,**23*)	Hydrolysis probe real-time PCR on buffy coat (*21**,**23*); IgM and IgG indirect immunofluorescent assay of admission and follow-up serum samples (4-fold rise considered positive result) (*24*)
Community-acquired bacterial infection	Gram stain; culture in blood agar and chocolate agar	Blood culture bottle
*Streptococcus pneumoniae, Streptococcus suis, Haemophilus influenzae*, *Neisseria meningitidis*	Culture on blood agar, chocolate agar, and MacConkey plates (for patients <1 year of age); hydrolysis probe real-time RT-PCR (*25**–**27*)	Blood culture bottle
Dengue virus	Hydrolysis probe real-time RT-PCR (*28*); NS1 ELISA (Dengue Early ELISA, Panbio, https://www.alere.com); ELISA IgM (Japanese Encephalitis/Dengue IgM Combo ELISA, Panbio)	Hydrolysis probe real-time RT-PCR on serum samples (*28*); NS1 ELISA on serum samples; ELISA IgM on admission and follow-up serum samples (assessed seroconversion)
JEV§	ELISA IgM (Japanese Encephalitis/Dengue IgM Combo ELISA, Panbio)	ELISA IgM on admission and follow-up serum samples (assessed seroconversion)
Enterovirus, West Nile virus, influenza viruses A and B, Henipavirus	Hydrolysis probe real-time RT-PCR (*29**–**31*) (in house)	Hydrolysis probe real-time RT-PCR on serum samples (*29**–**31*) (in house)
Flavivirus¶	SYBR Green real-time RT-PCR (*32**,**33*)	SYBR Green real-time RT-PCR on serum samples (*32**,**33*)
Herpes simplex virus 1 and 2, Varicella zoster virus, human cytomegalovirus	Hydrolysis probe real-time RT-PCR (*34**–**36*)	None
Measles virus, mumps virus	Hydrolysis probe real-time RT-PCR (*37**,**38*); if seropositive in blood sample, IgM ELISA with Enzygnost Anti-Measles Virus/IgM or Enzygnost Anti-Parotidis/IgM kits (Dade Behring, https://www.healthcare.siemens.com)	Hydrolysis probe real-time RT-PCR on serum samples (*37**,**38*); IgM and IgG ELISAs: Enzygnost Anti-Measles Virus/IgM, Enzygnost Anti-Measles Virus/IgG, Enzygnost Anti-Parotidis/IgM, and Enzygnost Anti-Parotidis/IgG kits (Dade Behring) (assessed seroconversion)
HIV	None	Determine HIV-1/2 Combo (Alere, https://www.alere.com) or Uni-Gold HIV (Trinity Biotech, https://www.trinitybiotech.com)

*See Appendix (https://wwwnc.cdc.gov/EID/article/25/5/18-0914-App1.pdf) for further details on methods. Cerebrospinal fluid and serum samples were inoculated on Vero and buffalo green monkey kidney cells for viral isolation. The median (interquartile range) interval between admission and follow-up serum sample collection was 9 (6–16) days. EMJH, Ellinghausen-McCullough-Johnson-Harris; JEV, Japanese encephalitis virus; NS1, nonstructural protein 1; RT-PCR, reverse transcription PCR. †Contraindications for lumbar puncture included focal neurologic signs on examination, clinical evidence for raised intracranial pressure, skin or soft tissue sepsis at the proposed puncture site, severe coagulopathy, or severe thrombocytopenia. ‡Buffy coats were inoculated on Vero and L929 cells for *O. tsutsugamushi* and *Rickettsia* sp isolation. §Detection of JEV IgM in a single sample of serum is considered as laboratory confirmation according to World Health Organization criteria. However, in this study, to be conservative and consistent with interpretation of other test results, a single detection of JEV IgM in serum was not counted as confirming JEV central nervous system infection. ¶Flavivirus RT-PCR would have likely detected the main flaviviruses circulating in Laos.

### Interpretation

The confirmed etiology was determined by the results of a panel of diagnostic tests (Table 2), which included tests for the direct detection of pathogens in CSF or blood, specific IgM in CSF, seroconversion, or a 4-fold rise in antibody titer between admission and follow-up serum samples. Pathogen detection was confirmed after critical analysis of test results to rule out possible contamination. When evidence of >1 pathogen was obtained for a patient, we defined the confirmed etiology as detection by direct tests over indirect tests (antibody-based tests) and prioritized CSF detection over blood detection (*39*). We defined a confirmed co-infection as the direct (or indirect, if only indirect tests were positive) detection of >1 pathogen in the same matrix (CSF or blood).

### Statistical Analyses

We compared patients with confirmed bacterial infection (including co-infections involving only bacteria) and patients with confirmed viral infection (including co-infections involving only viruses) with all other patients, excluding those with mixed co-infections (i.e., co-infections with fungi or infections with both bacteria and viruses). We investigated the factors associated with death (died in hospital or discharged moribund), bacterial infection, or viral infection by univariate analysis using the χ^2^ or Fisher exact test for categorical variables or the Mann-Whitney U test for continuous variables. We analyzed the independent predictors of death, bacterial infection, and viral infection using multivariate logistic regression models. In multivariate analyses, we included all factors having a p value <0.010 in the univariate analysis.

For variables with 6%–20% of the values missing, we executed multiple imputation models using chained equations and used a number of imputations that exceeded the highest proportion of missing values (*40*). We added age and sex to imputation models as auxiliary variables. We specified the imputation methods as linear for continuous normally distributed variables, logistic for binary variables, ordered logistic for ordinal variables, and predictive mean matching for continuous skewed variables. We performed logistic regression with the dependent variable (death, bacterial or viral infection) and all relevant covariates on each imputed data set and combined results using Rubin rules to take into account the variability in estimates among imputed data sets (*41*). Only variables that were significant (p<0.050) were retained in the final models. For comparison of analysis methods, we provided the results using the corresponding complete case analysis. We conducted analyses using Stata/SE version 14.0 (StataCorp, https://www.stata.com).

## Results

### Patients

**Figure 2:**
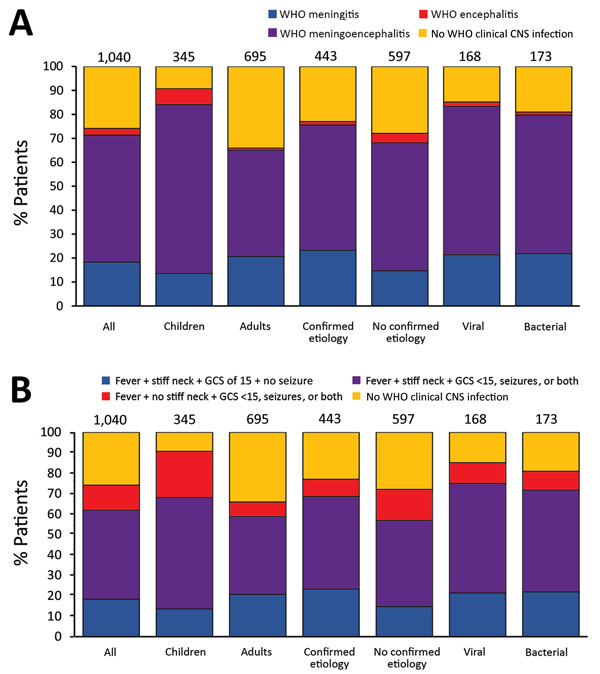
Distribution of clinical manifestations among patients with suspected CNS infection, by age group and etiology, Laos, January 2003–August 2011. A) WHO criteria; B) additional criteria (Table 1). Children were patients <15 years of age and adults patients >15 years of age. Numbers above bars indicate number of patients in group. CNS, central nervous system; GCS, Glasgow Coma Scale; WHO, World Health Organization.

**Figure 3:**
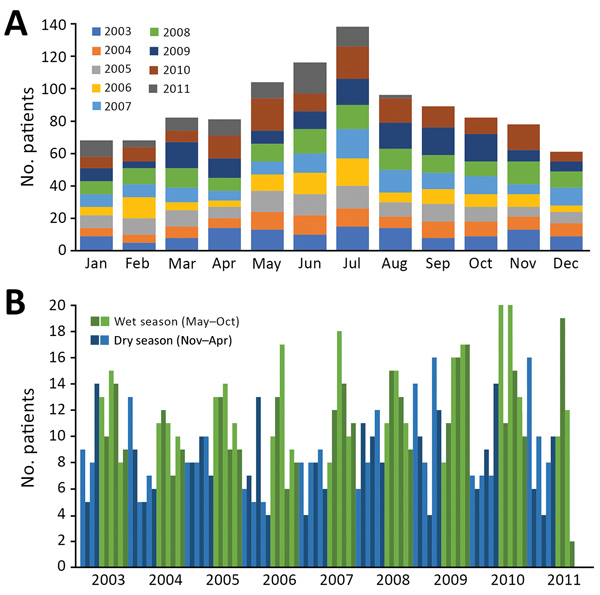
Recruited patients with suspected central nervous system infection, by month, Laos, January 2003–August 2011. A) Total patients recruited by month cumulating all studied years. B) Patients recruited each month of each year. Light and dark shades of colors were used in an alternating pattern to facilitate graph reading.

In total, 1,065 inpatients with suspected CNS infection consented to study participation (Appendix Figure 1); 80% were recruited from the pediatric and adult intensive care wards and adult infectious disease ward. On each ward, ≈8 physicians were in charge of patient recruitment. All were general hospital or infectious disease physicians; none were neurologists. We collected information on patient demographics, clinical presentation, and blood and CSF parameters (Table 3–4; Figure 2). More patients were recruited during the rainy season (Figure 3). The median time between admission and follow-up blood collection was 9 (interquartile range [IQR] 6–16) days. One third (33.6%, 358/1,065) were children <15 years of age (Table 3; Appendix Table 2).

**Table 3:** Demographic and clinical data at admission of patients with suspected CNS infection, by age group and etiology, Laos, January 2003–August 2011*

Characteristic					Etiology			
	Age group				Confirmed, n = 450	None confirmed, n = 615	Confirmed viral, n = 172	Confirmed bacterial, n = 175
	All, n = 1,065	Children, n = 358	Adults, n = 707					
Demographic								
M	666 (62.5)	207 (57.8)	459 (64.9)		288 (64.0)	378 (61.5)	111 (64.5)	117 (66.9)
F	399 (37.5)	151 (42.2)	248 (35.1)		162 (36.0)	237 (38.5)	61 (35.5)	58 (33.1)
Age, y, median (IQR)	23 (8–38)	3 (0.41–8)	32 (24–47)		23 (10–38)	24 (6–40)	16 (7–28)	23 (9–45)
History								
HIV seropositive, n = 703	119 (16.9)	1 (0.4)	118 (24.8)		75 (27.1)	44 (10.33)	8 (8.0)	6 (6.2)
Diabetes, n = 850	24 (2.8)	0	24 (4.2)		12 (3.5)	12 (2.4)	1 (0.8)	10 (7.5)
Antibiotic use before lumbar puncture, n = 953	590 (61.9)	238 (71.9)	352 (56.6)		252 (64.0)	338 (60.5)	109 (69.9)	100 (62.5)
Signs and symptoms								
Days of fever at admission, median (IQR), n = 1,058	4 (2–8)	4 (2–6)	5 (2–10)		5 (3–10)	4 (1–7)	5 (3–7)	5 (3–8)
Fever, n = 1,059	962 (90.8)	340 (95.2)	622 (88.6)		425 (94.9)	537 (87.9)	162 (95.3)	171 (97.7)
Headache,† n = 893	787 (88.1)	155 (83.3)	632 (89.4)		369 (92.5)	418 (84.6)	139 (90.9)	135 (91.2)
Hearing loss,† n = 893	51 (5.7)	10 (5.4)	41 (5.8)		20 (5.0)	31 (6.3)	8 (5.2)	7 (4.7)
Dysuria,† n = 891	28 (3.1)	4 (2.2)	24 (3.4)		10 (2.5)	18 (3.7)	3 (2.0)	3 (2.0)
Visual loss,† n = 885	66 (7.5)	14 (7.7)	52 (7.4)		23 (5.8)	43 (8.8)	11 (7.2)	5 (3.4)
Diplopia,† n = 889	36 (4.1)	4 (2.2)	32 (4.5)		15 (3.4)	21 (4.3)	6 (4.0)	6 (4.1)
Photophobia, n = 850	52 (5.8)	14 (7.4)	38 (5.4)		23 (5.8)	29 (5.9)	7 (4.6)	10 (6.8)
Focal neurologic signs, n = 939	22‡ (2.3)	5 (1.6)	17 (2.7)		8 (2.1)	14 (2.5)	1 (0.7)	6 (4.1)
Neck stiffness, n = 1,064	683 (64.2)	245 (68.4)	438 (62.0)		316 (70.2)	367 (59.8)	130 (75.6)	128 (73.1)
Confusion, n = 1,060	608 (57.4)	232 (65.5)	376 (53.3)		254 (56.7)	354 (57.8)	114 (66.3)	103 (59.5)
Convulsions, n = 1,063	319 (30.0)	233 (65.3)	86 (12.2)		119 (26.5)	200 (32.6)	65 (37.8)	44 (25.3)
GCS score, median (IQR), n = 1,010	14 (11–15)	14 (10–15)	15 (11–15)		15 (11–15)	14 (10–15)	13 (10–15)	14 (11–15)
GCS score <15,§ n = 1,047	551 (52.6)	220 (63.4)	331 (47.3)		225 (50.5)	326 (54.2)	101 (59.4)	94 (54.0)
WHO clinical CNS infection,¶ n = 1,040	771 (74.1)	313 (90.7)	458 (65.9)		341 (77.0)	430 (72.0)	143 (85.1)	140 (80.9)
Outcome								
Days of hospitalization, n = 846, median (IQR)	9 (5–14)	8 (5–13)	10 (5–15.5)		11 (6–17)	8 (5–13)	10 (6–14)	11 (7–17)
Death,# n = 893	235 (26.3)	70 (22.5)	165 (28.4)		94 (25.0)	141 (27.3)	23 (15.7)	43 (27.9)

*Values are no. (%) unless indicated otherwise. We defined children as patients <15 years of age and adults >15 years of age. History or physical examination or both, were taken into account for confusion, neck stiffness, photophobia, fever (history of fever or >37.5ºC during physical examination). In total, 8 women in the patient population were pregnant; 26 (2.4%) patients had computed tomography brain scans, and 2 of these scans demonstrated brain abscesses. The confirmed viral group includes patients infected with multiple viruses, and the confirmed bacterial group includes patients infected with multiple bacteria. CNS, central nervous system; GCS, Glasgow Coma Scale; IQR, interquartile range; WHO, World Health Organization.  †Data from children <3 years of age were considered not reliable and were thus excluded from analysis.  ‡Of these patients, 7 had hemiplegia, 11 had limb weakness, and 1 had paraplegia; 13 patients had admission or discharge diagnoses of Guillain-Barre syndrome. Retrospective evaluation of the likelihood of this diagnosis by using the Brighton system suggested that 4 patients met level 3 criteria for Guillain-Barre syndrome diagnostic certainty (*42*). §Includes confused and disoriented patients. ¶Defined as fever with GCS score <15, neck stiffness (history of or present during examination), or history of seizures or any of these signs in combination. Patients with missing data for 1 of these criteria were not counted. #Includes patients who died at the hospital and those taken home to die.

**Table 4:** Characteristics of peripheral blood and cerebrospinal fluid at admission of patients with suspected central nervous system infection, by age group and etiology, Laos, January 2003–August 2011*

Sample type and parameter	Age group				Etiology			
	All, n = 1,065	Children, n = 358	Adults, n = 707		Confirmed, n = 450	None confirmed, n = 615	Confirmed viral, n = 172	Confirmed bacterial, n = 175
Peripheral blood								
Elevated white cell count,† n = 952	449 (47.2)	150 (47.9)	299 (46.8)		198 (49.0)	251 (45.8)	84 (53.9)	84 (53.5)
Low white cell count, n = 952	45 (4.7)	22 (7.0)	23 (3.6)		22 (5.5)	23 (4.2)	6 (3.9)	7 (4.5)
Anemia, n = 948	355 (37.5)	112 (35.7)	243 (38.3)		160 (39.8)	195 (35.7)	44 (28.2)	68 (43.9)
Thrombocytopenia, n = 649	55 (8.5)	16 (6.8)	39 (9.4)		22 (7.8)	33 (9.0)	4 (3.5)	12 (10.6)
Elevated C-reactive protein, n = 868	547 (63.0)	145 (51.6)	402 (68.5)		265 (69.2)	282 (58.1)	98 (64.9)	114 (79.7)
Hyperglycemia,† n = 991	237 (23.9)	81 (25.8)	156 (23.0)		105 (24.5)	132 (23.5)	40 (24.0)	53 (32.3)
Severe hyperglycemia,† n = 991	72 (7.3)	26 (8.3)	46 (6.8)		35 (8.2)	37 (6.6)	12 (7.2)	22 (13.4)
Elevated serum sodium,‡ n = 807	225 (27.9)	45 (17.8)	180 (32.5)		82 (22.8)	143 (31.9)	40 (28.6)	26 (19.4)
Low serum sodium,‡ n = 807	63 (7.8)	31 (12.3)	32 (5.8)		31 (8.6)	32 (7.1)	8 (5.7)	16 (11.9)
Cerebrospinal fluid								
Turbid, n = 999	145 (14.5)	40 (12.2)	105 (15.7)		80 (18.4)	65 (11.5)	21 (12.4)	38 (23.2)
Elevated opening pressure, n = 977	334 (34.2)	86 (27.6)	248 (37.3)		155 (36.4)	179 (32.5)	42 (24.9)	60 (37.3)
Elevated white cell count,§ n = 975	729 (74.8)	237 (74.8)	492 (74.8)		341 (80.2)	388 (70.6)	141 (84.9)	129 (80.1)
Elevated lymphocyte count, n = 890	467 (52.5)	149 (51.2)	318 (53.1)		234 (59.5)	233 (46.9)	106 (68.4)	91 (62.3)
Elevated neutrophil count, n = 889	644 (72.4)	213 (73.5)	431 (72.0)		309 (78.8)	335 (67.4)	130 (83.9)	116 (80.0)
Elevated eosinophil count,¶ n = 1,001	46 (4.6)	7 (2.1)	39 (5.8)		11 (2.5)	35 (6.2)	9 (5.3)	2 (1.2)
Elevated protein, n = 955	601 (62.9)	177 (57.3)	424 (65.6)		281 (66.9)	320 (59.8)	112 (66.3)	108 (69.7)
Decreased glucose, n = 957	280 (29.3)	58 (18.8)	222 (34.3)		138 (32.8)	142 (26.5)	45 (26.6)	51 (32.9)
Decreased cerebrospinal fluid:venous glucose ratio, n = 929	540 (58.1)	159 (54.8)	381 (59.6)		253 (61.7)	287 (55.3)	97 (58.8)	97 (64.2)
Elevated lactate, n = 985	650 (66.0)	217 (67.8)	433 (65.1)		298 (69.8)	352 (63.1)	93 (56.0)	132 (80.5)

*Values are no. (%). We defined children as patients <15 years of age and adults >15 years of age. The confirmed viral group includes patients infected with multiple viruses, and the confirmed bacterial group includes patients infected with multiple bacteria. Elevated and low parameters mean above or below reference ranges. Anemia is defined as hematocrit below reference range. Thrombocytopenia is defined as platelet count below reference range. See Appendix Table 3 for reference ranges. CSF, cerebrospinal fluid. †Hyperglycemia was defined as a blood glucose level of >7.7 mmol/L and severe hyperglycemia as a blood glucose level >11.1 mmol/L. ‡Serum sodium levels >150 mmol/L were considered elevated and <130 mmol/L considered low; 5 patients (0.6%) had serum sodium <115 mmol/L. §Samples with high turbidity could not be counted and were thus not included. ¶An eosinophil count >10% was considered elevated.

Of 476 adults tested for HIV, 118 (24.8%) were seropositive; of 227 children tested, 1 (0.4%) was seropositive. More than half (61.9%, 590/953) of patients had a history or hospital chart evidence of antibiotic use before lumbar puncture. Most (90.8%, 962/1,059) patients had a history of fever or documented admission fever. The median length of fever at admission was 4 (IQR 2–8) days. The most frequent symptoms and signs were headache (88.1%, 787/893); neck stiffness (64.2%, 683/1,064); confusion (57.4%, 608/1,060); GCS score <15 (52.6%, 551/1,047); and vomiting, diarrhea, or both (54%, 575/1,064). All symptoms and signs were more frequent in children than adults (p<0.05), except headache, which was more frequent in adults (p = 0.023). Most (93.6%, 832/889) patients had CSF findings outside reference ranges (elevated CSF white cell count, elevated CSF lactate, elevated CSF protein, low CSF glucose, or any combination of these parameters) (Table 4; Appendix Table 3, Figure 2).

**Figure 4:**
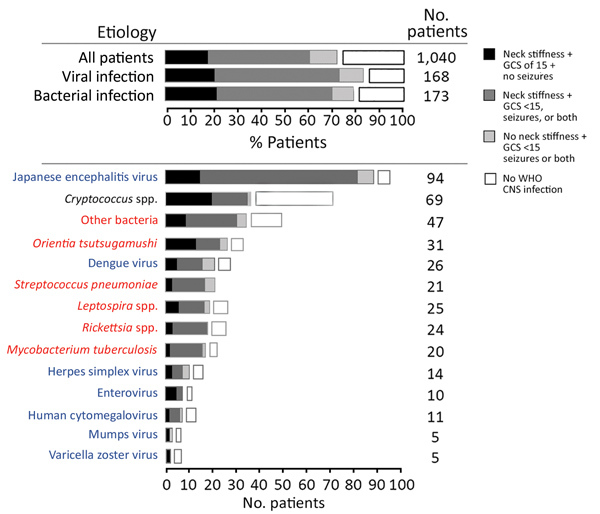
Distribution of clinical presentations in patients with suspected central nervous system infection, by confirmed etiology, Laos, January 2003–August 2011. Analysis per pathogen includes only patients with monoinfections. Other bacteria include 7 *Escherichia coli*, 4 *Streptococcus agalactiae*, 4 *Neisseria meningitidis*, 1 *Salmonella enterica* group D, 1 *S.* *enterica* group B or C, 5 *S.* *enterica* serovar typhi, 4 *Streptococcus suis*, 3 *Klebsiella pneumoniae*, 7 *Haemophilus influenzae* type b, 5 *Burkholderia pseudomallei*, 6 *Staphylococcus aureus*, and 1 *Morganella morganii.* Blue font indicates viruses, red font indicates bacteria, and black font indicates fungi. CNS, central nervous system; GCS, Glasgow Coma Scale; WHO, World Health Organization.

Etiology was confirmed in 450 (42.3%) patients; 413 (38.8%) had monoinfections and 37 (3.5%) co-infections (Appendix Tables 4–8). The identified monoinfections were JEV (8.8%, 94/1,065), *Cryptococcus* spp. (6.6%, 70/1,065; 9 *C. gattii*), *Orientia tsutsugamushi* (2.9%, 31/1,065), dengue virus (2.5%, 27/1,065), *Leptospira* spp. (2.3%, 25/1,065), *Rickettsia* spp. (2.3%, 24/1,065), *S. pneumoniae* (2.1%, 22/1,065), *M. tuberculosis* (1.9%, 20/1,065), HSV-1 or HSV-2 (1.4%, 15/1,065), human cytomegalovirus (1.1%, 12/1,065), enterovirus (0.9%, 10/1,065), varicella zoster virus (0.6%, 6/1,065), mumps virus (0.5%, 5/1,065), and *Plasmodium falciparum* (0.4%, 4/1,065). Other bacteria were detected in 48 (4.5%) patients (Figure 4; Appendix Table 5). All samples were negative for West Nile virus, influenza A and B, Henipavirus, and measles virus by PCR. Infection by *M. tuberculosis*, *Cryptococcus* spp., or varicella zoster virus was not detected in children (Appendix Table 8). The median age of children with enterovirus infection was 4.5 (IQR 1–11) years and JEV infection 13 (IQR 8–20) years. The proportion of patients with JEV infection was higher for children (14%, 50/358) than adults (6%, 44/707, p<0.001). Significantly more enterovirus patients (80%) than nonenterovirus patients (33%; p = 0.002) were children.

### Factors Associated with Bacterial and Viral Infections

We compared patients with single (n = 170) or multiple (n = 5) bacterial infections (excluding co-infections with viruses or fungi) with all other patients (n = 875). Factors significantly associated with bacterial infections on univariate analysis (p<0.01; Appendix Table 9) were included in multivariate analysis. Diabetes (adjusted odds ratio [aOR] 3.1, 95% CI 1.2−7.7), history of fever or fever at admission (aOR 3.9, 95% CI 1.4−11.1), higher serum C-reactive protein (aOR 1.08, 95% CI 1.05−1.11), and higher CSF lactate (aOR 3.5, 95% CI 2.3−5.4) were independent predictors of bacterial infection (Appendix Table 10).

We compared patients with single (n = 169) or multiple (n = 3) viral infections (excluding co-infections with bacteria or fungi) with all other patients (n = 867). Factors significantly associated with viral infections on univariate analysis (p<0.01; Appendix Table 11) were included in multivariate analysis. Neck stiffness (aOR 1.9, 95% CI 1.3−2.8) and higher hematocrit (aOR 1.4, 95% CI 1.1−1.9) were associated with viral infection, whereas higher CSF lactate (aOR 0.3, 95% CI 0.1–0.5), older age (aOR 0.8, 95% CI 0.7–0.9), and longer interval between admission and lumbar puncture (aOR 0.9, 95% CI 0.8–1.0) were negatively associated with viral infection (Appendix Table 12).

### Relationships between Clinical Presentation and Etiology

In total, 771 (74.1%) of 1,040 patients had WHO CNS infection; 44.2% of these patients had confirmed etiologies compared with 37.9% of patients not fulfilling WHO CNS infection criteria (p = 0.063; Appendix Table 13). Because of the considerable overlap between the WHO encephalitis and meningitis definitions, 551 (71.5%) patients were classified as having meningoencephalitis. Therefore, we analyzed the frequency of neck stiffness, reduced GCS score, and seizures among febrile patients with clinical CNS infection (Table 1).

When comparing viral and bacterial infections, we observed no significant differences (p>0.05) in the proportions of encephalitis and meningitis syndromes, although differences were observed for some specific etiologies (Figure 4). In total, 90 (53.6%) febrile patients with viral infection and 86 (49.7%) with bacterial infection had neck stiffness and reduced GCS score, seizures, or both; 17 (10.1%) patients with viral infection and 16 (9.3%) with bacterial infection had reduced GCS score, seizures, or both without neck stiffness; and 36 (21.4%) patients with viral infection and 38 (22.0%) with bacterial infection had neck stiffness, a GCS score of 15, and no seizures (Figure 4). We obtained similar results using the WHO definitions. In total, 25 (14.9%) patients with viral infection and 33 (19.1%) with bacterial infection did not fulfill the WHO CNS infection definition.

In comparison with the distribution of syndromes observed for all patients, the distribution in patients with some etiologies were significantly different (p<0.05). Of the 89 JEV patients with WHO CNS infection, 75.3% had fever; neck stiffness; and reduced GCS score, seizures, or both. Of the 26 *O. tsutsugamushi* patients with WHO CNS infection, 50% had fever, neck stiffness, a GCS score of 15, and no seizures. Of note, almost half (47.8%) of the patients with cryptococcal infection did not fulfill the definition for WHO CNS infection, and of the 36 who did, 55.6% had fever, neck stiffness, a GCS score of 15, and no seizures.

### Risk Factors for Death

Of 893 patients, 235 (26.3%) died, including those discharged moribund; we compared them to the 658 (73.7%) patients discharged alive and not moribund. For factors significantly associated with death (p<0.01; Appendix Table 14) in the univariate analysis, we conducted multivariate analysis. The variables strongly associated with death were higher CSF lactate (aOR 1.1, 95% CI 1.0–1.1) and reduced GCS score (aOR 0.8, 95% CI 0.8–0.9). Patients with viral infection were less likely to die than those with other diagnoses (aOR 0.4, 95% CI 0.3–0.7) (Appendix Table 15). Diabetes and hyperglycemia (glucose >7.7 mmol/L) at admission were not associated with death.

### Indications for Antibiotic Treatment

In total, 56 patients (12.4% of the 450 patients with confirmed etiologies) were infected with bacteria treatable by ceftriaxone and 64 patients (14.2% of the patients with confirmed etiologies) with bacteria treatable by doxycycline but not ceftriaxone (Table 5). Twenty-eight patients were infected with a *Leptospira* spp. treatable by ceftriaxone or doxycycline, but 2 were co-infected with *O. tsutsugamushi* not treatable by ceftriaxone. Of 142 patients infected by bacteria treatable by ceftriaxone or doxycycline, 89 (62.7%) received appropriate treatment, 17% (13/77) of whom died. In comparison, 25% (12/48) of the patients who did not receive appropriate treatment died (p = 0.270).

**Table 5:** Frequency of criteria consistent with bacterial meningitis among patients with suspected central nervous system infection, by etiology and antibiotic susceptibility, Laos, January 2003–August 2011*

Variables	Patients with confirmed etiology, n = 450							Patients without confirmed etiology, n = 615	Total, n = 1,065
	All	Patients infected by bacteria treatable by†					Other, n = 305		
		Ceftriaxone			Doxycycline				
		Not including *Leptospira* infections, n = 56‡	Including *Leptospira* infections, n = 84		Not including *Leptospira* infections, n = 64§	Including *Leptospira* infections, n = 90			
Neck stiffness¶	316 (70.2)	41 (73.2)	60 (71.4)		46 (71.9)	63 (70.0)	213 (69.8)	367 (59.8)	683 (64.2)
GCS score <15	225 (50.5)	34 (61.8)	47 (56.6)		27 (42.2)	40 (44.4)	152 (50.3)	326 (54.2)	551 (52.6)
Elevated CRP	265 (69.2)	44 (91.7)	60 (87.0)		36 (70.6)	51 (72.9)	171 (64.3)	282 (58.1)	547 (63.0)
CSF turbid	80 (18.4)	27 (49.1)	31 (37.8)		6 (10.7)	9 (11.1)	45 (15.1)	65 (11.5)	145 (14.5)
Elevated CSF lactate	298 (69.8)	44 (83.0)	63 (78.8)		44 (74.6)	62 (73.8)	193 (66.3)	352 (63.1)	650 (66.0)
Elevated CSF protein	281 (66.9)	44 (81.5)	57 (73.1)		32 (62.7)	43 (58.9)	195 (66.3)	320 (59.8)	601 (62.9)
Decreased CSF glucose	138 (32.8)	23 (42.6)	26 (33.3)		12 (23.5)	15 (20.5)	101 (34.2)	142 (26.5)	280 (29.3)
Decreased CSF:venous glucose ratio	253 (61.7)	40 (76.9)	49 (64.5)		27 (54)	35 (48.6)	179 (62.4)	287 (55.3)	540 (58.1)
Elevated CSF leukocyte count#	341 (80.2)	44 (86.3)	64 (82.1)		39 (69.6)	57 (70.4)	241 (82.0)	388 (70.6)	729 (74.8)
Combinations of >1 of the above findings									
Elevated CSF lactate, protein, leukocyte count; decreased CSF glucose; CSF turbid#	418 (95.9)	53 (96.4)	76 (93.8)		54 (90.0)	75 (89.3)	291 (97.7)	534 (93.2)	952 (94.4)
Elevated CRP; elevated CSF lactate, protein; CSF turbid	427 (96.4)	56 (100)	82 (100)		59 (92.2)	83 (94.3)	289 (96.3)	526 (93.4)	953 (94.7)
Elevated CRP; elevated CSF lactate, protein	425 (95.4)	56 (100)	82 (100)		58 (92.1)	82 (94.3)	288 (96.3)	525 (93.4)	950 (94.7)
Elevated CRP; elevated CSF lactate	385 (91.2)	54 (98.2)	78 (98.7)		56 (91.8)	78 (94.0)	254 (89.1)	478 (88.5)	863 (89.7)
Elevated CRP; elevated CSF protein	382 (90.1)	54 (98.2)	75 (94.9)		49 (89.1)	68 (88.3)	261 (89.1)	442 (84.2)	824 (86.8)
Elevated CRP; GCS score <15	348 (83.9)	50 (100.0)	72 (98.6)		49 (86.0)	70 (89.7)	229 (79.5)	448 (83.1)	796 (83.4)
GCS score <15; elevated CSF protein	348 (81.1)	49 (90.7)	68 (85.0)		44 (77.2)	61 (75.3)	239 (81.0)	454 (80.8)	802 (80.9)
GCS score <15; elevated CSF lactate	361 (84.1)	48 (88.9)	69 (85.2)		50 (83.3)	70 (82.4)	244 (83.8)	466 (80.3)	827 (82.0)
GCS score <15; elevated CSF lactate, protein	404 (92.9)	52 (94.5)	75 (91.5)		53 (88.3)	74 (87.1)	279 (94.3)	515 (89.4)	919 (90.9)

*All values are no. (%). See Appendix Table 3 for reference ranges. Only patients with confirmed etiology strictly sensitive to ceftriaxone or doxycycline are included in the analysis. Classification was based on a combination of susceptibility testing of isolates from patients and information from Principles and Practice of Infectious Diseases (*43*). Patients who were confused or disoriented who had their GCS score missing were considered to have a GCS score <15. CRP, C-reactive protein; CSP, cerebrospinal fluid; GCS, Glasgow Coma Scale. †In total, 28 patients were infected with *Leptospira* spp. treatable by either ceftriaxone or doxycycline, but 2 were also co-infected with *Orientia tsutsugamushi* not treatable with ceftriaxone. One patient co-infected with *Streptococcus suis* and *Rickettsis typhi* required therapy with both ceftriaxone and doxycycline. ‡Includes 24 patients infected with *Streptococcus pneumoniae* and 32 infected with other bacteria (7 *Escherichia coli*, 4 Group B *Streptococcus*, 4 *Neisseria meningitidis*, 1 *Salmonella enterica* group D, 1 *S. enterica* group B or C, 5 *S. suis*, 5 *S. enterica* serovar Typhi, 2 *Klebsiella pneumoniae*, 2 *Haemophilus influenzae*, and 1 *Edwardsiella tarda*). §Includes 31 patients with *Rickettsia* spp. infection and 33 with *O. tsutsugamushi* infection. ¶History of neck stiffness or neck stiffness on examination. #Samples with high turbidity could not be counted and were thus not included.

Including the 450 patients with confirmed diagnoses, we analyzed the criteria for bacterial meningitis commonly considered when making decisions on antibiotic treatment: elevated CSF white cell count, elevated CSF lactate, elevated CSF protein, decreased CSF glucose, reduced GCS score, turbid CSF, and neck stiffness. A low percentage (<23%) of patients with any 1 of these criteria (except turbid CSF, 38.8%) or a combination of these criteria had bacterial infections treatable by ceftriaxone or doxycycline (Appendix Table 16). Furthermore, only 1 combination of criteria (elevated C-reactive protein, elevated CSF protein, or elevated CSF lactate or any combination of these criteria) could identify all patients infected with bacteria treatable by ceftriaxone (Table 5). However, because only 5% of our patient series did not display this combination of criteria, none of the analyzed clinical and biological results can be reliably used to guide decisions on antibiotic use.

## Discussion

Etiology was confirmed in 42.3% of patients with suspected CNS infection, consistent with regional published data (Appendix Table 1); 16.2% had viral infections, and 16.4% had bacterial infections. We observed no significant differences in the distribution of clinical encephalitis and meningitis syndromes by bacterial or viral etiology; the most common infections in this patient population were JEV (8.8%) and *Cryptococcus* spp. (6.6%).

The results of this study provided evidence for the implementation of pneumococcal immunization in 2011 and JEV immunization in 2013 in Laos (*16*,*17*). Although the main etiology reported among patients with suspected CNS infection was JEV, this finding might be an overestimate; we have noted that the detection of JEV IgM in CSF has low predictive value (*44*). On the other hand, bacterial causes were probably underestimated; 61.9% of patients were known or thought to have received an antibiotic before lumbar puncture, potentially rendering bacteria uncultivable or reducing the bacterial load below the threshold needed for molecular detection.

The mortality rate we report in our study (26.3%) was higher than those reported in similar studies in neighboring countries (≈10%; Appendix Table 1). Ineffective patient management or inappropriate treatment through lack of previous local data might have caused this higher mortality rate. The epidemiology of CNS infection varies by geography; therefore, regional evidence should be used to build regional policies on prevention, diagnosis, and treatment of these infections.

In this study, 17% (119/703) of the patients tested were HIV seropositive. The highest proportion of HIV-seropositive patients was among those with cryptococcal infection (79%; Appendix Table 7). However, only 66% (703/1,065) of patients were tested. More patients need to receive HIV testing in Laos, and more investigations on the relationship between HIV and other infections are needed.

Our study had a number of limitations, including the partial use of stored samples; missing values; a low frequency of computed tomography brain scans and HIV testing; and a lack of magnetic resonance imaging, brain or postmortem examination, and diagnostics for autoimmune and eosinophilic CNS disease (*45*,*46*) and other pathogens (e.g., *Toxoplasma gondii*, *Mycoplasma* spp., and Zika virus). The absence of strict criteria for the inclusion of patients could have resulted in recruitment bias; however, the data reflect real-life medical practice. The proportion of patients who declined lumbar puncture is unknown. Almost all patients (93.6%) had CSF findings outside reference ranges. Although published data on this combined index are few, the proportion of patients with abnormal CSF findings is generally lower in routine practice (e.g., <40% at La Timone Hospital, Marseille; L. Ninove and J. Fromonot, La Timone Hospital, pers. comm., March 2017). This finding suggests a relatively low frequency of lumbar puncture at Mahosot Hospital, reflecting current practice but representing an unknown proportion of all patients admitted with CNS disease. The sample size was too small for a comparison of mortality rates between treated and nontreated patients.

Although CNS infection is a global public health burden, global consensus on the case definition is lacking (Appendix Table 17). In clinical studies, encephalitis and meningitis have been studied separately or together (*11*), and CSF findings might or might not be taken into account (e.g., CSF findings are not part of the WHO criteria). There is confusion regarding the clinical and laboratory definitions of encephalitis and meningitis, so we suggest pairing clinical, laboratory, or clinicolaboratory with these terms to reduce confusion. Further, altered consciousness and altered mental status, shared by encephalitis and meningitis in many definitions, are undefined in the WHO definitions. We used GCS score <15 to define both objectively, but this practice resulted in considerable overlap in clinical definitions: 71.4% of patients had WHO-defined meningitis and 53% WHO-defined meningoencephalitis. When we restricted the definition of meningitis to the presence of fever and neck stiffness, 61.9% of patients fulfilled those criteria; 43.6% had neck stiffness combined with low GCS score, seizures, or both.

Studies on the clinical and etiologic characteristics of patients requiring lumbar puncture in Asia have usually focused on particular pathogens or just meningitis or encephalitis (Appendix Table 1) (*47*). Although needed for treatment trials and pathophysiologic research, our data call into question the validity of defining criteria for patient management differently between encephalitis and meningitis (Figure 4; Appendix Table 13). In Laos, evidence suggests that brain (encephalitis) and meningeal (meningitis) infections have no clear distinguishable clinical manifestations relating to the responsible pathogen and that these classifications should be used with caution in the Asia tropics for guiding patient management.

We found that history of diabetes was independently associated with bacterial CNS infection. Indeed, some evidence suggests that diabetes is a risk factor for bacterial CNS disease (*48*) and poor outcome in tuberculous meningitis (*49*). In univariate analysis, higher blood glucose level was also associated with bacterial infection (p<0.001). Of 237 patients with hyperglycemia at admission (>7.7 mmol/L), 16 (6.8%) had a history of diabetes and 164 (69.2%) did not. Without convalescent glucose and hemoglobin A1c assays, however, we were unable to distinguish hyperglycemia resulting from severe disease or undiagnosed diabetes that might have predisposed to CNS infection. Intensive euglycemia management is difficult; it can lead to hypoglycemia, especially in unconscious patients, and requires skilled dedicated nursing that is not available in hospitals in rural Asia. Whether such intensive therapy would save lives remains uncertain, but the development of an inexpensive computerized algorithm technology for resource-poor settings to facilitate safe euglycemia management (*50*) should be a priority for investigation of efficacy. The burgeoning global prevalence of diabetes calls for research regarding the relationship between hyperglycemia and CNS infections and optimizing their combined management (*48*).

Our data suggest that patient survival could be improved through 2 patient management interventions, the implementation of antibiotic use guidelines and strengthening of high-dependency units. The finding that poor outcomes were associated with a decreased GCS score at admission suggests that high-dependency units (a likely cost-effective intervention) could be used to enhance supportive care for unconscious patients with CNS infection. Creating these units and incorporating them into care could improve outcomes and reduce the burden of intensive care unit treatment for these patients. More investigation is needed on the efficacy and cost-effectiveness of high-dependency units in different contexts.

Ceftriaxone is conventionally used in Laos as a first-line treatment for CNS bacterial infection but lacks efficacy for emerging rickettsial pathogens, for which doxycycline is recommended (*4*). Because delays in antibiotic therapy could result in severe consequences for patients, the decision for administering these drugs is made on the basis of clinical signs and laboratory results at admission. However, in Laos, we found that no variable, even in combination, could permit objective selection of appropriate antibiotics. Therefore, the administration of early first-line empiric treatment with ceftriaxone and doxycycline for all patients with suspected CNS infection might save patient lives in Laos and elsewhere in rural Asia (*4*).

Appendix Additional information on management of central nervous system infections in patients in Vientiane, Laos, January 2003–August 2011.

## References

[1] Tarantola A, Goutard F, Newton P, de Lamballerie X, Lortholary O, Cappelle J, et al. Estimating the burden of Japanese encephalitis virus and other encephalitides in countries of the mekong region. PLoS Negl Trop Dis. 2014;8:e2533. 10.1371/journal.pntd.0002533 24498443 PMC3907313

[2] Glaser CA, Gilliam S, Schnurr D, Forghani B, Honarmand S, Khetsuriani N, et al.; California Encephalitis Project, 1998-2000. In search of encephalitis etiologies: diagnostic challenges in the California Encephalitis Project, 1998-2000. Clin Infect Dis. 2003;36:731–42. 10.1086/367841 12627357

[3] Glaser CA, Honarmand S, Anderson LJ, Schnurr DP, Forghani B, Cossen CK, et al. Beyond viruses: clinical profiles and etiologies associated with encephalitis. Clin Infect Dis. 2006;43:1565–77. 10.1086/509330 17109290

[4] Dittrich S, Rattanavong S, Lee SJ, Panyanivong P, Craig SB, Tulsiani SM, et al. *Orientia*, *rickettsia*, and *leptospira* pathogens as causes of CNS infections in Laos: a prospective study. Lancet Glob Health. 2015;3:e104–12. 10.1016/S2214-109X(14)70289-X 25617190 PMC4547322

[5] World Health Organization. Japanese encephalitis. 2015 Dec 31 [cited 2018 Jun 6]. https://www.who.int/en/news-room/fact-sheets/detail/japanese-encephalitis

[6] Solomon T, Dung NM, Vaughn DW, Kneen R, Thao LT, Raengsakulrach B, et al. Neurological manifestations of dengue infection. Lancet. 2000;355:1053–9. 10.1016/S0140-6736(00)02036-5 10744091

[7] Puccioni-Sohler M, Orsini M, Soares CN. Dengue: a new challenge for neurology. Neurol Int. 2012;4:e15. 10.4081/ni.2012.e15 23355928 PMC3555217

[8] Cam BV, Fonsmark L, Hue NB, Phuong NT, Poulsen A, Heegaard ED. Prospective case-control study of encephalopathy in children with dengue hemorrhagic fever. Am J Trop Med Hyg. 2001;65:848–51. 10.4269/ajtmh.2001.65.848 11791985

[9] Hendarto SK, Hadinegoro SR. Dengue encephalopathy. Acta Paediatr Jpn. 1992;34:350–7. 10.1111/j.1442-200X.1992.tb00971.x 1509881

[10] Ho Dang Trung N, Le Thi Phuong T, Wolbers M, Nguyen Van Minh H, Nguyen Thanh V, Van MP, et al.; VIZIONS CNS Infection Network. Aetiologies of central nervous system infection in Viet Nam: a prospective provincial hospital-based descriptive surveillance study. PLoS One. 2012;7:e37825. 10.1371/journal.pone.0037825 22662232 PMC3360608

[11] Flett KB, Rao S, Dominguez SR, Bernard T, Glode MP. Variability in the diagnosis of encephalitis by pediatric subspecialists: the need for a uniform definition. J Pediatric Infect Dis Soc. 2013;2:267–9. 10.1093/jpids/pis094 26619481

[12] Xie Y, Tan Y, Chongsuvivatwong V, Wu X, Bi F, Hadler SC, et al. A population-based acute meningitis and encephalitis syndromes surveillance in Guangxi, China, May 2007-June 2012. PLoS One. 2015;10:e0144366. 10.1371/journal.pone.0144366 26633824 PMC4669244

[13] Touch S, Hills S, Sokhal B, Samnang C, Sovann L, Khieu V, et al. Epidemiology and burden of disease from Japanese encephalitis in Cambodia: results from two years of sentinel surveillance. Trop Med Int Health. 2009;14:1365–73. 10.1111/j.1365-3156.2009.02380.x 19747185

[14] Olsen SJ, Campbell AP, Supawat K, Liamsuwan S, Chotpitayasunondh T, Laptikulthum S, et al.; Thailand Encephalitis Surveillance Team. Infectious causes of encephalitis and meningoencephalitis in Thailand, 2003-2005. Emerg Infect Dis. 2015;21:280–9. 10.3201/eid2102.140291 25627940 PMC4313633

[15] Dittrich S, Sunyakumthorn P, Rattanavong S, Phetsouvanh R, Panyanivong P, Sengduangphachanh A, et al. Blood-brain barrier function and biomarkers of central nervous system injury in rickettsial versus other neurological infections in Laos. Am J Trop Med Hyg. 2015;93:232–7. 10.4269/ajtmh.15-0119 26055741 PMC4530739

[16] Moore CE, Sengduangphachanh A, Thaojaikong T, Sirisouk J, Foster D, Phetsouvanh R, et al. Enhanced determination of *Streptococcus pneumoniae* serotypes associated with invasive disease in Laos by using a real-time polymerase chain reaction serotyping assay with cerebrospinal fluid. Am J Trop Med Hyg. 2010;83:451–7. 10.4269/ajtmh.2010.10-0225 20810803 PMC2929034

[17] Moore CE, Blacksell SD, Taojaikong T, Jarman RG, Gibbons RV, Lee SJ, et al. A prospective assessment of the accuracy of commercial IgM ELISAs in diagnosis of Japanese encephalitis virus infections in patients with suspected central nervous system infections in Laos. Am J Trop Med Hyg. 2012;87:171–8. 10.4269/ajtmh.2012.11-0729 22764310 PMC3391045

[18] World Health Organization. Recommended standards for surveillance of selected vaccine-preventable diseases. 2003 [cited 2018 Jun 6]. http://www.measlesrubellainitiative.org/wp-content/uploads/2013/06/WHO-surveillance-standard.pdf

[19] Thaipadungpanit J, Chierakul W, Wuthiekanun V, Limmathurotsakul D, Amornchai P, Boonslip S, et al. Diagnostic accuracy of real-time PCR assays targeting 16S rRNA and *lipL32* genes for human leptospirosis in Thailand: a case-control study. PLoS One. 2011;6:e16236. 10.1371/journal.pone.0016236 21283633 PMC3026019

[20] Cole JR Jr, Sulzer CR, Pursell AR. Improved microtechnique for the leptospiral microscopic agglutination test. Appl Microbiol. 1973;25:976–80. 4736794 10.1128/am.25.6.976-980.1973 PMC380950

[21] Jiang J, Chan T-C, Temenak JJ, Dasch GA, Ching W-M, Richards AL. Development of a quantitative real-time polymerase chain reaction assay specific for *Orientia tsutsugamushi.* Am J Trop Med Hyg. 2004;70:351–6. 10.4269/ajtmh.2004.70.351 15100446

[22] Jiang J, Stromdahl EY, Richards AL. Detection of *Rickettsia parkeri* and *Candidatus* Rickettsia andeanae in *Amblyomma maculatum* Gulf Coast ticks collected from humans in the United States. Vector Borne Zoonotic Dis. 2012;12:175–82. 10.1089/vbz.2011.0614 22022815

[23] Henry KM, Jiang J, Rozmajzl PJ, Azad AF, Macaluso KR, Richards AL. Development of quantitative real-time PCR assays to detect *Rickettsia typhi* and *Rickettsia felis*, the causative agents of murine typhus and flea-borne spotted fever. Mol Cell Probes. 2007;21:17–23. 10.1016/j.mcp.2006.06.002 16893625

[24] Phetsouvanh R, Blacksell SD, Jenjaroen K, Day NPJ, Newton PN. Comparison of indirect immunofluorescence assays for diagnosis of scrub typhus and murine typhus using venous blood and finger prick filter paper blood spots. Am J Trop Med Hyg. 2009;80:837–40. 10.4269/ajtmh.2009.80.837 19407134 PMC7610873

[25] Carvalho MG, Tondella ML, McCaustland K, Weidlich L, McGee L, Mayer LW, et al. Evaluation and improvement of real-time PCR assays targeting *lytA*, *ply*, and *psaA* genes for detection of pneumococcal DNA. J Clin Microbiol. 2007;45:2460–6. 10.1128/JCM.02498-06 17537936 PMC1951257

[26] Corless CE, Guiver M, Borrow R, Edwards-Jones V, Fox AJ, Kaczmarski EB. Simultaneous detection of *Neisseria meningitidis*, *Haemophilus influenzae*, and *Streptococcus pneumoniae* in suspected cases of meningitis and septicemia using real-time PCR. J Clin Microbiol. 2001;39:1553–8. 10.1128/JCM.39.4.1553-1558.2001 11283086 PMC87969

[27] Mai NTH, Hoa NT, Nga TVT, Linh D, Chau TTH, Sinh DX, et al. *Streptococcus suis* meningitis in adults in Vietnam. Clin Infect Dis. 2008;46:659–67. 10.1086/527385 19413493

[28] Leparc-Goffart I, Baragatti M, Temmam S, Tuiskunen A, Moureau G, Charrel R, et al. Development and validation of real-time one-step reverse transcription-PCR for the detection and typing of dengue viruses. J Clin Virol. 2009;45:61–6. 10.1016/j.jcv.2009.02.010 19345140

[29] Watkins-Riedel T, Woegerbauer M, Hollemann D, Hufnagl P. Rapid diagnosis of enterovirus infections by real-time PCR on the LightCycler using the TaqMan format. Diagn Microbiol Infect Dis. 2002;42:99–105. 10.1016/S0732-8893(01)00330-3 11858904

[30] Lanciotti RS, Kerst AJ, Nasci RS, Godsey MS, Mitchell CJ, Savage HM, et al. Rapid detection of west nile virus from human clinical specimens, field-collected mosquitoes, and avian samples by a TaqMan reverse transcriptase-PCR assay. J Clin Microbiol. 2000;38:4066–71. 11060069 10.1128/jcm.38.11.4066-4071.2000 PMC87542

[31] van Elden LJ, Nijhuis M, Schipper P, Schuurman R, van Loon AM. Simultaneous detection of influenza viruses A and B using real-time quantitative PCR. J Clin Microbiol. 2001;39:196–200. 10.1128/JCM.39.1.196-200.2001 11136770 PMC87701

[32] Moureau G, Temmam S, Gonzalez JP, Charrel RN, Grard G, de Lamballerie X. A real-time RT-PCR method for the universal detection and identification of flaviviruses. Vector Borne Zoonotic Dis. 2007;7:467–77. 10.1089/vbz.2007.0206 18020965

[33] Moureau G, Ninove L, Izri A, Cook S, De Lamballerie X, Charrel RN. Flavivirus RNA in phlebotomine sandflies. Vector Borne Zoonotic Dis. 2010;10:195–7. 10.1089/vbz.2008.0216 19492949 PMC3496374

[34] Kessler HH, Mühlbauer G, Rinner B, Stelzl E, Berger A, Dörr HW, et al. Detection of Herpes simplex virus DNA by real-time PCR. J Clin Microbiol. 2000;38:2638–42. 10878056 10.1128/jcm.38.7.2638-2642.2000 PMC86985

[35] Bousbia S, Papazian L, Saux P, Forel JM, Auffray J-P, Martin C, et al. Repertoire of intensive care unit pneumonia microbiota. PLoS One. 2012;7:e32486. 10.1371/journal.pone.0032486 22389704 PMC3289664

[36] Griscelli F, Barrois M, Chauvin S, Lastere S, Bellet D, Bourhis JH. Quantification of human cytomegalovirus DNA in bone marrow transplant recipients by real-time PCR. J Clin Microbiol. 2001;39:4362–9. 10.1128/JCM.39.12.4362-4369.2001 11724846 PMC88550

[37] Hummel KB, Lowe L, Bellini WJ, Rota PA. Development of quantitative gene-specific real-time RT-PCR assays for the detection of measles virus in clinical specimens. J Virol Methods. 2006;132:166–73. 10.1016/j.jviromet.2005.10.006 16274752

[38] Uchida K, Shinohara M, Shimada S, Segawa Y, Doi R, Gotoh A, et al. Rapid and sensitive detection of mumps virus RNA directly from clinical samples by real-time PCR. J Med Virol. 2005;75:470–4. 10.1002/jmv.20291 15648065

[39] Phommasone K, Paris DH, Anantatat T, Castonguay-Vanier J, Keomany S, Souvannasing P, et al. Concurrent Infection with murine typhus and scrub typhus in southern Laos—the mixed and the unmixed. PLoS Negl Trop Dis. 2013;7:e2163. 10.1371/journal.pntd.0002163 24009783 PMC3757080

[40] White IR, Royston P, Wood AM. Multiple imputation using chained equations: Issues and guidance for practice. Stat Med. 2011;30:377–99. 10.1002/sim.4067 21225900

[41] Rubin DB. Multiple imputation for nonresponse in surveys. New York: John Wiley and Sons; 1987.

[42] Sejvar JJ, Kohl KS, Gidudu J, Amato A, Bakshi N, Baxter R, et al.; Brighton Collaboration GBS Working Group. Guillain-Barré syndrome and Fisher syndrome: case definitions and guidelines for collection, analysis, and presentation of immunization safety data. Vaccine. 2011;29:599–612. 10.1016/j.vaccine.2010.06.003 20600491

[43] Bennett JE, Dolin R, Blaser MJ. Principles and practice of infectious diseases. 8th ed. Philadelphia: Elsevier Saunders; 2014.

[44] Dubot-Pérès A, Sengvilaipaseuth O, Chanthongthip A, Newton PN, de Lamballerie X. How many patients with anti-JEV IgM in cerebrospinal fluid really have Japanese encephalitis? Lancet Infect Dis. 2015;15:1376–7. 10.1016/S1473-3099(15)00405-3 26607119

[45] Eamsobhana P. Angiostrongyliasis in Thailand: epidemiology and laboratory investigations. Hawaii J Med Public Health. 2013;72(Suppl 2):28–32. 23901379 PMC3689488

[46] Newman MP, Blum S, Wong RCW, Scott JG, Prain K, Wilson RJ, et al. Autoimmune encephalitis. Intern Med J. 2016;46:148–57. 10.1111/imj.12974 26899887

[47] Taylor WR, Nguyen K, Nguyen D, Nguyen H, Horby P, Nguyen HL, et al. The spectrum of central nervous system infections in an adult referral hospital in Hanoi, Vietnam. PLoS One. 2012;7:e42099. 10.1371/journal.pone.0042099 22952590 PMC3431395

[48] Schut ES, Westendorp WF, de Gans J, Kruyt ND, Spanjaard L, Reitsma JB, et al. Hyperglycemia in bacterial meningitis: a prospective cohort study. BMC Infect Dis. 2009;9:57. 10.1186/1471-2334-9-57 19426501 PMC2694198

[49] Erdem H, Ozturk-Engin D, Tireli H, Kilicoglu G, Defres S, Gulsun S, et al. Hamsi scoring in the prediction of unfavorable outcomes from tuberculous meningitis: results of Haydarpasa-II study. J Neurol. 2015;262:890–8. 10.1007/s00415-015-7651-5 25634680

[50] Blaha J, Barteczko-Grajek B, Berezowicz P, Charvat J, Chvojka J, Grau T, et al. Space GlucoseControl system for blood glucose control in intensive care patients—a European multicentre observational study. BMC Anesthesiol. 2016;16:8. 10.1186/s12871-016-0175-4 26801983 PMC4722682

