# Supplementary material for: Management of Central Nervous System Infections, Vientiane, Laos, 2003–2011
Source: Emerg Infect Dis. 2019 May;25(5):898–910. doi: 10.3201/eid2505.180914 (PMC6478220; doi:10.3201/eid2505.180914)
Supplement: Appendix — Additional information on management of central nervous system infections in patients in Vientiane, Laos, January 2003–August 2011. [file 18-0914-Techapp-s1.pdf]

Article DOI: https://doi.org/10.3201/eid2505.180914

Management of Central Nervous System Infections, Vientiane, Laos, 2003–2011

Appendix

Laboratory Assays

Cerebral Spinal Fluid and Blood Parameters

Cerebral spinal fluid (CSF) opening pressure, using sterile spinal manometers (R55990; Rocket Medical plc, Washington, UK), and appearance were recorded. A CSF cell count was performed in an Improved Neubauer counting chamber, and slides (*1*) were prepared for Gram, Indian ink, and Giemsa stains using a cytospin (Shandon; Thermo Fisher Scientific, Waltham, USA). CSF glucose and protein were measured on a HumaStar 600 (HUMAN Diagnostics Worldwide, Wiesbaden, Germany) or Biochemistry Analyzer DS401 (SINNOWA, Nanjing, China) during working time and on Visual/70VB0357 (SECOMAM, Alès, France) during off duty hours, and lactate, using an Accutrend Plus System (Roche, Bâle, Switzerland). At the same time as the lumbar puncture, blood glucose was measured using ACCU-CHEK Advantage meters with Advantage II strips (Roche) from venous or capillary blood. On the same day, blood cultures (Pharmaceutical Factory no. 2, Vientiane, Lao PDR) (*2*), EDTA blood for complete blood count (CBC), and buffy coat and whole blood for serum and clot were drawn. CBCs were performed using HumaCount (5L, 60TS, or 80TS, HUMAN GmbH, Germany). Sera were sent to Bangkok (V-Diagnostic Center Co., Ltd) for additional biochemistry to measure C-reactive protein, creatinine, total bilirubin, alkaline phosphatase, alanine aminotransferase, aspartate aminotransferase on an Olympus AU400 automated analyzer.

CSF Culture

Blood agar and chocolate agar plates and a MacConkey plate for children <1 year of age were inoculated with 1 drop of CSF pellet each. Bacteria grown from blood cultures (*2*) and CSF were identified using standard microbiological methods, including colony morphology, Gram stain, biochemical gallery assays, and APIs (bioMérieux, Lyon, France). Antibiotic disc diffusion

susceptibility testing and Etests were performed according to the contemporary Clinical and Laboratory Standards Institute guidelines (2009). Gram, Auramine, *Ziehl–Neelsen*, and Indian ink stain microscopy were performed on the CSF pellet (*1*). Mahosot Hospital participates in the UK NEQAS scheme for General Bacteriology and Antimicrobial Susceptibility Testing and acid-fast bacilli microscopy.

Blood Culture Bottles

The blood culture bottles contain tryptic hydrolysate casein 1.7%, soy peptone 0.3%, sodium chloride 0.5%, potassium phosphate 0.25%, dextrose 0.25%, and sodium polyanetholsulfonate 0.025% in water for injection.

Cryptococcus spp. Detection

Cryptococcus spp. were detected by Indian ink staining of the CSF pellet for all patients and the *Cryptococcus* Antigen Latex Agglutination Test System (IMMY, Norman, USA) for patients with known or suspected HIV infection. CSF was cultured on Sabouraud agar if the Indian ink and/or cryptococcal antigen test were positive or if the patient was suspected to have cryptococcal meningitis, incubated in air at 30°C. Cultured *Cryptococcus* spp. were extracted for PCR and restriction fragment length polymorphism (RFLP) serotyping using the technique of Enache-Angoulvant et al. (*3*). Before PCR implementation (2008), colonies were identified using either the Crypto Check kit (Iatron Laboratories, Tokyo, Japan) or Canavanine-Glycine-Bromthymol Blue Agar (*4*).

M. tuberculosis Culture and Susceptibility Testing

In total, 200 µL of CSF pellet was inoculated on Lowenstein-Jensen Medium Slants (BBL, catalogue no. 220908; BD, Franklin Lakes, USA) for *M. tuberculosis* culture for 8 weeks. Presumptive mycobacteria were sent to the International Organization for Migration in Bangkok for confirmation (Accuprobe MTB Assay; Gen-Probe Incorporated, San Diego, CA) and phenotypic susceptibility testing (BACTEC MGIT 960 System; BD, Franklin Lakes, USA) and to the Centre d'Infectiologie Christophe Mérieux du Laos, Vientiane, for rifampin and isoniazid resistance identification using GenoType MTBDRplus Assay (Hain Lifescience, Nehren, Germany), according to World Health Organization recommendations (*5*). A few colonies were recovered from positive Lowenstein-Jensen culture with an aseptic inoculation loop and suspended in 300 µL of molecular grade water then incubated 20 minutes at 95°C in a

thermomixer (Eppendorf, Hamburg, Germany). After 5 minutes of centrifugation at 10,000 × g, 5 µL of supernatant was used to perform GenoType MTBDRplus PCR and reverse hybridization, following the manufacturer's instructions (Hain Lifescience, Nehren, Germany).

Leptospiral Culture

Culture of leptospires from blood clot was performed using 3 mL of Ellinghausen, McCullough, Johnson and Harris medium supplemented with 3% rabbit serum and 0.1% agarose in 5-mL sterile, plastic flat-based screw-cap tubes (Sterilin, Barloworld Scientific Ltd., UK). In total, 3 mL of Ellinghausen, McCullough, Johnson and Harris medium was added to the blood clot remaining after centrifugation of ≈5 mL whole blood, and serum was removed using a sterile pipette and left overnight at room temperature. The next morning, the supernatant was transferred into a new 5-mL tube and incubated at Lao room temperature (≈25°C) for 12 weeks. Leptospires were identified by dark-field microscopy at ×200 magnification (*6*).

Leptospiral Microscopic Agglutination Tests

Microscopic agglutination tests were performed for all admission sera and follow-up sera when available following the technique developed by Cole et al. (*7*). Two-fold serial dilutions of serum were prepared using phosphate-buffered saline (PBS). Antigens, *Leptospira* cultures adjusted to 100–200 organisms per high-power field (450×), were mixed with all serum dilutions in microplate wells and incubated at room temperature (25°C–30°C) for 2 hours. The plates were examined under microscope for agglutination. The endpoint in a positive test was the highest dilution in which at least 50% of the leptospires were agglutinated. Patients were regarded as positive if their paired sera demonstrated a 4-fold rise in antibody titer (*8*). Serovars included in the panel were Pomona, Hardjo, Tarassovi, Grippotyphosa, Celledoni, Copenhageni, Australis, Pyrogenes, Canicola, Hebdomadis, Mini, Saxkoebing, Sarmin, Autumnalis, Cynopteri, Ballum, Bataviae, Djasiman, Javanica, Panama, Shernani, Var 10, and Mwalok.

O. tsutsugamushi and *Rickettsia* spp. Culture

In total, 200 µL of buffy coat was mixed with 3 mL of cell culture medium (RPMI with 10% fetal calf serum; GIBCO, Thermo Fisher Scientific); then, two 12.5-cm^2 flasks, one of confluent Vero cells and the other of confluent L929 cells, were inoculated with half of the buffy coat mixture each. The flasks were centrifuge for 30 min at 500 rpm then put for 2 hour in a CO_2 (5%) incubator at 35°C. Then, the culture media were removed and replaced by 5 mL of fresh

media. The day after, half of the culture media was removed and replaced by fresh media. Then, twice a week, culture media were completely replaced by fresh media. Four weeks after inoculation, the cultures were tested by immunofluorescence assay (IFA) to check for *Rickettsia* or *O. tsutsugamushi* growth. A small surface of cell layer was scraped and the recovered cells were washed 1 time with PBS then diluted 1:5 in PBS and loaded onto a slide. The slide was fixed in acetone for 10 minutes at –20°C. After drying, it was washed in PBS for 5 minutes. An antibody solution was prepared in PBS with 1:800 of each antibody (STG, SFG, TG) and 2% skim milk and loaded on the slide. The slide was incubated in a wet chamber at 35°C for 30 minutes. After 3 PBS washings, secondary antibody (1:50 FITC in PBS with 2% skim milk and 0.00125% Evans blue) was loaded on the slide; then, the slide was incubated in a wet chamber at 35°C for 30 minutes. After 3 PBS washings, the slide was read under ultraviolet light. The Evans blue stains the Vero and L929 cells red, and *Rickettsia* or *O. tsutsugamushi* green. In case of positive, IFAs with separate antibodies were performed for identification. In case of culture negative, flasks were incubated 8 additional weeks then rechecked by IFA.

IFA for Antibodies against *Orientia tsutsugamushi* and *Rickettsia typhi*

Acute and follow-up sera were tested by IFA to detect the presence of either IgM or IgG antibodies to *O. tsutsugamushi* (indicating scrub typhus infection) and to *R. typhi* (indicating murine typhus infection). In total, 4 μL of serum was diluted to 1:25 in a microtitration plate with autoclaved PBS plus 3% skim milk powder. These sera were serially diluted 2-fold from 1:25–1:12,800. A 2-μL aliquot of each serum dilution was added to IFA slides coated with antigen from *O. tsutsugamushi* strains (Karp, Kato, and Gilliam serotypes; Australian Rickettsial Reference Laboratory, Geelong, Victoria, Australia) and an *R. typhi* strain (Wilmington; Australian Rickettsial Reference Laboratory) then incubated in a moist chamber at 37°C for 1 hour. Slides were then washed 3 times (5 minutes/wash) with autoclaved PBS. After washing and drying, the slides were treated with specific fluorescein isothiocyanate–conjugated goat anti-human γ chain immunoglobulin (Sigma Aldrich, Munich, Germany), incubated for 30 minutes at 37°C, washed 3 times (5 min/wash) with autoclaved PBS, and mounted in buffered glycerol (90% [v/v] glycerol and 10% PBS). The IFA slides were read with an ECLIPSE E600 microscope (Nikon Co., Tokyo, Japan). The endpoint of each IFA titer was defined as the lowest serum concentration demonstrating definite fluorescence. Each slide contained positive and

negative controls, which were examined before interpreting the sample result (*9*). A positive result was defined as a 4-fold rise in IgM or IgG titer between admission and follow-up sera (*10*).

Viral ELISAs

Dengue virus and *Japanese encephalitis virus* (JEV) ELISA kits (Panbio Inc., Brisbane, Australia, now Alere Inc.) were used to detect *Dengue virus* NS1 (Dengue Early ELISA, E-DEN01P) and IgM against *Dengue virus* and JEV (Japanese Encephalitis/Dengue IgM combo ELISA, E-JED01C) in CSF, admission sera, and follow-up sera, following the manufacturer's instructions. The IgM combo ELISA permitted distinguishing anti-JEV IgM from anti-dengue IgM by testing both in the same sample on the same plate and comparing their results following an algorithm provided by the manufacturer. For CSF, the dilution 1:10 was used (*11*). Detection of anti-JEV IgM in a single sample of serum is considered as laboratory confirmation according to World Health Organization criteria. However, in this study, to be conservative and consistent with interpretation of other test results, a single detection of anti-JEV IgM in serum was not counted as confirming JEV central nervous system (CNS) infection.

Admission and follow-up sera were tested by ELISA for the detection of anti-measles and anti-mumps IgG and IgM using the Measles Enzygnost IgG and IgM kits and Mumps Enzygnost IgG and IgM kits (Dade Behring, Deerfield, IL, USA). If serum was positive, the corresponding CSF, when available, was tested for anti-measles or anti-mumps virus IgM.

Virus Isolation in Cell Culture

A cell culture facility was not available at the beginning of the study, and different cells were made available over time. For patients 357–1,073, supernatant after CSF centrifugation (450 × g for 20 min) was inoculated on Vero cell, and for patients 897–1,073, admission serum was also inoculated on Vero cells. For patients 967–1,073 the BGM cell line was used for CSF and serum inoculation.

In a Biosafety level 3 laboratory, 200 µL of patients' samples were inoculated onto confluent cells in a 12-well plate format. After 1 week at 37°C in a 5% CO_2 incubator, cells were scraped and 0.2 mL was passaged onto a fresh 12-well plate. In case of cytopathic effect, cells were scraped, and 1 mL was passaged onto a fresh 25-cm^2 flask. Isolated viruses were identified by specific real-time PCR after nucleic acid extraction using QIAamp MinElute Virus Spin Kit (QIAGEN, Hilden, Germany).

Molecular Assays

Nucleic Acid Extraction

DNA extraction from 200 µL of pellet after CSF centrifugation (450 × g for 20 min) was performed by using the QIAamp DNA Mini kit (QIAGEN) (*12*) with the modification that lysozyme (5 µL at a concentration of 10 mg/mL) and mutanolysin (5 µL at a concentration of 10 mg/mL) (Sigma Aldrich) were added during a 30-minute lysis step at 37°C, as described by Moore et al. (*13*). DNA was eluted in 80 µL of QIAGEN elution buffer.

EDTA buffy coat samples (200 µL) were extracted with QIAamp DNA Mini kits (QIAGEN), according to the manufacturer's instructions, with the only exception of an extended lysis step from 10 min–1 h at 56°C. DNA was eluted in a final volume of 100 µL.

Cryptoccocus spp. culture isolates were extracted using QIAamp DNA Mini kit (QIAGEN) using the protocol for bacterial cultures, with an additional lysis step, 10 µL of lyticase (10 mg/mL), added to the ATL buffer and incubated at 37°C for 30 minutes, before the addition of proteinase K.

Viral Nucleic Acid Extraction

For viral RNA and DNA, 200 µL of serum on admission and 200 µL of CSF were extracted with EZ1 Virus Mini Kit v2.0, using a BioRobot EZ1 Workstation (QIAGEN), by following the manufacturer's instructions. The elution volume was 90 µL. A fixed amount of RNA and DNA bacteriophages (MS2 and T4, respectively) was added to all samples before extraction to be used as internal controls as previously described (*14*).

PCR Analysis

All sequences of primers and probes are displayed in Appendix Table 18.

Cryptococcus Typing PCR

In total, 5 µL of DNA from *Cryptococcus* culture were submitted to conventional PCR targeting *CAP59* gene as described by Enache-Angoulvant et al. (*3*), in a 50-µL final volume with 6 mmol/L $MgCl_2$, 200 µM of dNTPs, 120 nmol/L of each primer, 1 U of AmpliTaq Gold DNA Polymerase (Applied Biosystems, Thermo Fisher Scientific). The PCR thermal profile was 95°C for 10 min and 35 cycles of 95°C for 30 sec, 58°C for 30 sec, and 72°C for 2 min. In total, 10 µL of PCR product were then submitted to RFLP using *Age1-HF* (0.2 U), *BsmFI* (0.05 U), or

HpaII (0.2 U), enzymes from New England Biolabs (Ipswich, MA, USA), in a final volume of 20 μL with 2 μg of bovine serum albumin incubated 1 hour at 65°C for *BSmFI*, 1 hour at 37°C for *Age1-HF*, and 1 hour at 37°C for *HpaII*. Amplification of *Cryptococcus neoformans* var. *neoformans* is cut only by *HpaII*, *C. neoformans* var. *grubii* only by *BSmFI*, and *Cryptococcus gattii* by *Age1-HF* and *HpaII*. Restriction fragments were checked on a 3% agarose gel. For quality control, *C. neoformans* var. *grubii*, *C. gattii* (the prominent pathogenic cryptococci in southeast Asia), as well as no-template controls were included in every PCR and RFLP run.

Leptospira PCR

The hydrolysis probe real-time quantitative PCR (qPCR) developed by Thaipadungpanit et al. (*15*), targeting *rrs* gene, was used to detect *Leptospira* spp. in buffy coat and CSF. The assay was optimized for use in a RotorGene machine (QIAGEN) using the Platinum Taq DNA Polymerase kit (Invitrogen, Thermo Fisher Scientific) in a final volume of 20 μL with 200 μM of dNTPs, 250 nmol/L of forward primer, 500 nmol/L of reverse primer, 50 nmol/L of probe, 1 U of Taq, and 5 μL of DNA. The qPCR thermal profile was 50°C for 2 min, 95°C for 8 min, and 45 cycles of 95°C for 15 sec and 60°C for 1 min. Positives were confirmed by sequencing.

PCR for *Streptococcus pneumoniae*, *Haemophilus influenzae*, *Neisseria meningitidis*, and *Streptococcus suis*

DNA extracted from CSF was tested for *S. pneumoniae*, *S. suis*, *H. influenzae*, and *N. meningitidis* by using 4 simplex hydrolysis probe qPCRs previously described (*16–18*). The primer and probe conditions were optimized to be used with AmpliTaq Gold DNA Polymerase (Applied Biosystems, Thermo Fisher Scientific) and a RotorGene machine (QIAGEN). The final volume of reaction mixes was 25 μL, containing 200 μM of dNTPs; 1 U of Taq; 5 mmol/L of $MgCl_2$; 300 nmol/L of each primer and 100 nmol/L of probe for *H. influenzae*, 200 nmol/L of each primer and 100 nmol/L of probe for *S. pneumoniae*, 300 nmol/L of each primer and 25 nmol/L of probe for *N. meningitidis*, and 400 nmol/L of each primer and 100 nmol/LM of probe for *S. suis*; and 3 μL of DNA. The thermal cycling program used was 95°C for 10 min followed by 40 cycles of 95°C for 15 sec and 60°C for 60 sec.

S. pneumoniae typing

Positive samples for *S. pneumoniae* were submitted to additional hydrolysis probe qPCRs for serotying as developed by Moore et al. (*13*); 3 μL of DNA was used for each qPCR. In total,

12 primer pairs and locked nucleic acid probes were designed to target the *cps* gene of 18 serotypes and were used in 3 multiplex and a simplex qPCR: serotypes 1, 3, 4, and 5 in multiplex 1; serotypes 6A/B, 7A/F, 9A/L/N/V, and 14 in multiplex 2; serotypes 18B/C, 19F, and 23F in multiplex 3; and serotype 19A in the simplex qPCR. All PCRs were optimized for the Corbett Rotor-Gene 6000 series (QIAGEN) in 25-µL final reaction volumes, with 5.5 mmol/L $MgCl_2$; 200 µM of dNTPs; 1 U AmpliTaq Gold DNA polymerase (Thermo Fisher Scientific); 240 nmol/L of each primer for multiplexes 1 and 2 and 300 nmol/L of each primer for multiplex 3 and serotype 19A; and 40 nmol/L of probe for serotypes 3 and 7A/F, 80 nmol/L of probe for other serotypes of multiplexes 1 and 2, 50 nmol/L of probe for serotype 19A, and 100 nmol/L of probe for other serotypes of multiplex 3 and 19A. The thermal cycling program used was 95°C for 10 min and 45 cycles of 95°C for 15 sec and 60°C for 1 min.

S. pneumonia isolates, when available, were sent to Murdoch Children Research Institute. Serotyping was performed by latex agglutination using a combination of commercial and in-house typing reagents (*19*), and results were confirmed using the Quellung reaction.

H. influenzae typing

Positive CSF or isolates, when available, were sent to Haemophilus Reference Laboratory in the United Kingdom (Respiratory and Vaccine Preventable Bacteria Reference Unit, Public Health England, Colindale) for *H. influenzae* typing by hydrolysis probe qPCR based on Maaroufi et al. (*20*).

This consisted of a triplex hydrolysis probe qPCR for *ompP2* (detection of all *H. influenzae*), *bexA* (to detect the capsule operon in any capsulated strains), and *H. influenzae* specific target (based on the *H. influenzae* type b [Hib] specific region of the capsule operon) using 12.5 µL of TaqMan universal master mix (Applied Biosystems, Thermo Fisher Scientific) and 1 µL of DNA. The oligonucleotide concentrations used were 900 nmol/L for *ompP2* reverse primer and Hib forward and reverse primers, 300 nmol/L for *ompP2* forward primer and bexA forward and reverse primers, 50 nmol/L for *ompP2* probe, 500 nmol/L for *bexA* probe, and 250 nmol/L for Hib probe. The cycling parameters were 50°C for 2 min and 95°C for 10 min, followed by 40 cycles of 95°C for 15 sec and 58°C for 1 min.

N. meningitidis typing

DNA from positive samples for *N. meningitidis* were sent to Meningococcal Reference Unit, Health Protection Agency, Manchester, UK, for typing by hydrolysis probe qPCR assay based on Meningococcal Reference Unit and Corless et al. methods (*17*,*21*). Modification has been made as improvement of *CtrA* System and the use of freeze-dried Taqman Quadruplex assay (*22*). The quadruplex contains primers against *N. meningitides* capsule transporter (*ctrA*), serogroup B sialyltransferase (*siaD*$_B$), *S. pneumoniae* pneumolysin (ply), and an internal control (*Cucurbita cv. Kurokawa amakuri* hydroxypyruvate reductase). The assay was prepared by Applied Biosystems (Thermo Fisher Scientific) in a lyophilized format, with primer and probe sequences provided by the MRU; the components of the master mix have not been disclosed by the company. Lyophilized reagents were rehydrated with 20 µL of molecular-grade water, and then, 5 µL of DNA was added. Amplification and detection was done on TaqMan 7500 (Applied Biosystems, Thermo Fisher Scientific) using fast cycling conditions (2 min at 95°C, 45 cycles of 95°C for 3 sec and 60°C for 30 sec).

Orientia tsutsugamushi PCR

This hydrolysis probe qPCR was based on that described by Jiang et al. (*23*) targeting the *47-kD* gene. In total, 1 µL of DNA extract from EDTA buffy coat and 5 µL for CSF was used in a 25-µL reaction with the Platinum Quantitative PCR SuperMix-UDG (Invitrogen) kit and 100 nmol/L of each primer and 200 nmol/L of probe. The thermal cycling program was 50°C for 2 min, 95°C for 2 min, and 45 cycles of 95°C for 15 sec and 60°C for 60 sec. All positive qPCRs were confirmed by sequencing (Macrogen Inc) or conventional PCR targeting 56 kDa as previously described (*24*).

Rickettsia genus and *Rickettsia typhi* PCR

This assay is a hydrolysis probe qPCR targeting the 17-kDa gene of *Rickettsia* spp. (*23*,*25*) using 1 µL of DNA extracted from the EDTA buffy coat and 5 µL for CSF, in a 25-µL reaction volume. The Platinum Quantitative PCR SuperMix-UDG kit (Invitrogen, Thermo Fisher Scientific) was used in a final volume of 25 µL, with 400 nmol/L of each primer and probe. The thermal cycling program was 50°C for 2 min, 95°C for 2 min, and 45 cycles of 95°C for 15 sec and 60°C for 30 sec.

Rickettsia genus–positive samples were confirmed as *Rickettsia typhi* using a confirmatory hydrolysis probe qPCR, targeting *ompB* gene, as described by Henry et al. (*26*). In total, 1 µL of buffy coat DNA and 5 µL of CSF DNA was used in a 25-µL reaction volume, with the Platinum Quantitative PCR SuperMix-UDG (Invitrogen, Thermo Fisher Scientific) and 400 nM of each primer and probe. The thermal cycling program was 50°C for 2 min, 95°C for 2 min, and 45 cycles of 95°C for 15 sec and 60°C for 30 sec. Repeatedly ompB-positive samples were processed for sequencing, following a conventional PCR targeting the *17-kDa* gene, to identify the *Rickettsia* species. Conventional PCR was performed using Platinum Taq DNA polymerase (Invitrogen, Thermo Fisher Scientific), 300 nmol/L of each primer, forward 1 and reverse, 0.2 mmol/L of dNTPs, 2 mmol/L of $MgCl_2$, 1 U of Taq, and 1 µL of DNA in a final volume of 25 µL. The thermal cycling program was 94°C for 1 min and 34 cycles of 94°C for 30 sec, 55°C for 30 sec, and 68°C for 2 min, ending with 72°C for 7 min. A nested PCR is performed using the same conditions as the first PCR, with the same reveres primer and forward 2 primer on 1 µL of the first PCR product. The PCR product of the nested PCR was sent to Macrogen Inc. (Seoul, South Korea) for purification and sequencing. Sequencing results were identified using NCBI nucleotide BLAST.

Viral PCR

Protocols for virus detection were transferred from Virology Laboratory at La Timone Hospital, Marseille, France, where they are used for routine diagnosis, to the microbiology laboratory of Mahosot Hospital.

Real-time PCRs for the detection of herpes simplex virus (HSV) 1 and 2 (*27*), human cytomegalovirus (HCMV) (*28*), varicella zoster virus (VZV) (*29*), *West Nile virus* (WNV) (*30*), *Tick-borne encephalitis virus* (TBEV) (*31*), *Enterovirus* (EV) (*32*), *Dengue virus* (*33*), Henipavirus (in house system), panflavivirus (*34*,*35*), measles virus (*36*), mumps virus (*37*), and influenza viruses A and B (*38*) were performed on CSF and admission serum for all patients when available.

The HSV1/2 system permits to detect HSV1 and HSV2 viruses. Samples positive by HSV1/2 PCR were submitted to 2 specific hydrolysis probe qPCRs for the detection of HSV1 and HSV2 (*39*). Detection of *Dengue virus* was done using a pan-dengue hydrolysis probe qPCR system designed to detect the 4 dengue serotypes. Positive samples were then submitted to the 4

hydrolysis probe qPCRs specific for the 4 serotypes. The hydrolysis probe qPCR used for the detection of EV is a pan-EV system that permits detection of all enteroviruses. Typing of EV-positive samples was performed following techniques developed by Nix et al. (*40*), see below, directly on patient sample extract or after inoculation on cell culture, when possible.

The primers and probe for detection of *Henipavirus* were designed using alignment of all Hendra and Nipah virus sequences available in GenBank.

PCR conditions were adapted to a standard 2-step protocol using TaqMan Reverse Transcription Reagents kit (Roche) for RNA viruses, followed by hydrolysis probe qPCR using Eurogentec Mastermix for probe assay (Eurogentec, Liège, Belgium) for HSV1/2, HSV1, HSV2, VZV, HCMV, EV, *Dengue virus*, *Dengue virus* 1, *Dengue virus* 2, *Dengue virus* 3, *Dengue virus* 4, WNV, TBEV, measles virus, mumps virus, influenza viruses A and B (until September 2009), and *Henipahvirus* detection. For RNA viruses, 10 µL of viral nucleic acid extract was submitted to random reverse transcription (RT) using Transcription Reagents kit (Roche) and hexamer primers following the manufacturer's instructions in a final volume of 50 µL. Simplex qPCR was then performed on 10 µL of DNA (RT product for RNA viruses and extract for DNA viruses) using 25 µL of qPCR MasterMix (Eurogentec, Liège, Belgium), 200 nmol/L of each primer, and 80 nmol/L of probe in a final volume of 50 µL. qPCRs were performed using Mx3000P QPCR System (Agilent Technologies, Santa Clara, CA USA) with standard thermal cycling (50°C for 2 min, 95°C for 10 min, and 45 cycles of 95°C for 15 sec and 60°C for 1 min). WNV and TBEV primers and probes were used in a duplex qPCR following the same protocol. Any samples positive with a cycle quantification (C_q) <40 were repeated for confirmation.

Internal controls (MS2 and T4, RNA and DNA bacteriophages, respectively) were added to all specimens and systematically tested by hydrolysis probe qPCR (*14*). T4 and MS2 qPCRs were performed on 3 µL of DNA (RT product for MS2, nucleic acid extract for T4) in a final volume of 15 µL. In case of no detection of internal control, a new sample was extracted. In case of inhibition of the PCR, the extract sample was diluted 1:10 in AVE buffer (QIAGEN), and all qPCR reactions were repeated from this dilution.

Duplex hydrolysis probe qPCR was performed for the detection of influenza viruses A and B until September 2009 following the protocol above. *Influenzavirus* A qPCR was shown to not detect pandemic H1N1/09 (*41*), so primers alone were used in a SYBR Green RT-qPCR.

Since September 2009, influenza virus A and B primers were used to perform a duplex SYBR Green RT-qPCR using QuantiTect SYBR Green RT-PCR kit (QIAGEN) on 5 µL of viral nucleic acid with 560 nmol/L of each primer in a final volume of 25 µL. The thermal cycling program was 50°C for 30 min; 95°C for 15 min; and 45 cycles of 94°C for 15 sec, 60°C for 30 sec, and 72°C for 45 sec, ending with a melting curve from 60°C to 95°C. A positive sample has a peak around 79°C for *Influenzavirus B* and 80°C for *Influenzavirus A*.

A panflavivirus SYBR Green real-time RT-PCR that detects all viruses belonging to the genus *Flavivirus* (family *Flaviviridae*) was performed using QuantiTect SYBR Green RT-PCR kit (QIAGEN) on 5 µL of viral nucleic acid with 550 nmol/L of each primer (forward 1 and reverse) in a final volume of 25 µL. The thermal cycling program was 50°C for 30 min; 95°C for 15 min; and 45 cycles of 94°C for 15 sec, 50°C for 30 sec, and 72°C for 45 sec, ending with a melting curve from 60°C–95°C. A positive sample shows a peak around 80°C. Amplicons (270 bp in the NS5 gene) were sequenced (Macrogen Inc.) and the corresponding sequences were BLASTed on the NCBI Web site (blastn) for identification. All negative primary panflavivirus PCRs underwent a heminested PCR using 3 µL of the primary PCR product, the same reverse primer, the forward 2 primer, and the same amplification protocol as in the primary PCR. Amplicons (≈162 bp) were sent for sequencing to Macrogen Inc. Then, the sequences were BLASTed (blastn, NCBI website) for identification.

Enterovirus typing

The typing of EV was performed using the protocol from the French reference center for Enterovirus based on Nix et al. (*40*). When available, clinical samples EV-positive by RT-qPCR were inoculated on MRC5, BGM, and MA104 cells in 12-well plates. In cases of cytopathic effect, cell supernatant was collected, extracted using EZ1 Virus Mini Kit v2.0 (QIAGEN), and submitted to pan-EV hydrolysis probe RT-qPCR using SuperScript III Platinum One-Step qRT-PCR kit (Invitrogen) with 200 nmol/L of each primer, 100 nmol/L of probe, and 5 µL of extract in 25 µL final volume. The thermal cycling program was 50°C for 15 min, 95°C for 2 min, and 45 cycles of 95°C for 15 sec and 60°C for 45 sec. The extracts from EV-positive cultures were submitted for RT-PCR using the Access RT-PCR system (Promega) with 1 µmol/L of each forward primer and reverse primer 1 and 5 µL of extract in a final volume of 50 µL, following the manufacturer's instructions, with 42°C as the annealing temperature. The thermal cycling

program was 45°C for 45 min; 94°C for 2 min; and 40 cycles of 94°C for 30 sec, 42°C for 1 min, and 68°C for 2 min, ending with 68°C for 7 min.

For patients, whose EV could not be isolated by cell culture, 5 µL of extract underwent RT using 100 U of SuperScript III Reverse Trancriptase (Invitrogen), 10 mmol/L dithiothreitol, 0.1 mmol/L dNTP, 200 nmol/L of each RT primer (1–4), and 20 U RNaseOUT Recombinant Ribonuclease (Invitrogen) in a 10-µL final volume. The RT thermal cycling program was 22°C for 10 min, 50°C for 50 min, and 95°C for 5 min. Primary PCR was performed on RT products using 2.5 U of AmpliTaq DNA Polymerase (Applied Biosystems), 1 µmol/L of forward primers and reverse primer 1, and 0.2 mmol/L of dNTP in a 50-µL final volume. The thermal cycling program used was 95°C for 5 min and 40 cycles of 95°C for 30 sec, 42°C for 50 sec, and 60°C for 50 sec.

The primary PCR with primers 1 produce a 990-bp amplicon. In case of negative primary PCR, a nested PCR was performed with 5 µL of the primary PCR product using 2.5 U of FastStart Taq DNA Polymerase (Roche), 800 nmol/L of each primer 2, and 0.2 mmol/L dNTP in a 50-µL final volume. The thermal cycling program was 95°C for 5 min and 40 cycles of 95°C for 30 sec, 60°C for 50 sec, and 72°C for 30 sec.

Nested PCR with primers 2 produce a 375-bp amplicon. Amplicons from primary or nested PCR were sent for sequencing to Macrogen Inc. Then, the sequences were BLASTed (blastn, NCBI website) for identification.

qPCR Interpretation

For quality control, positive and nontemplate controls were included in each run. A PCR was classified as positive if an amplification curve with a C_q value $\leq$40 was observed from the same sample in 2 separate PCR runs.

References

1. Cheeseborough M. District laboratory practice in tropical countries. 2nd ed. Vol. 2. Cambridge: Cambridge University Press; 2006.

2. Phetsouvanh R, Phongmany S, Soukaloun D, Rasachak B, Soukhaseum V, Soukhaseum S, et al. Causes of community-acquired bacteremia and patterns of antimicrobial resistance in Vientiane,

Laos. Am J Trop Med Hyg. 2006;75:978–85. PubMed http://dx.doi.org/10.4269/ajtmh.2006.75.978

3. Enache-Angoulvant A, Chandenier J, Symoens F, Lacube P, Bolognini J, Douchet C, et al. Molecular identification of *Cryptococcus neoformans* serotypes. J Clin Microbiol. 2007;45:1261–5. PubMed http://dx.doi.org/10.1128/JCM.01839-06

4. Kwon-Chung KJ, Polacheck I, Bennett JE. Improved diagnostic medium for separation of *Cryptococcus neoformans* var. *neoformans* (serotypes A and D) and *Cryptococcus neoformans* var. *gattii* (serotypes B and C). J Clin Microbiol. 1982;15:535–7. PubMed

5. World Health Organization. Molecular line probe assays for rapid screening of patients at risk of multidrug-resistant tuberculosis (MDR-TB). Policy statement. 2008 Jun 27 [cited 2018 Jun 6]. https://www.who.int/tb/features_archive/policy_statement.pdf

6. Wuthiekanun V, Chierakul W, Limmathurotsakul D, Smythe LD, Symonds ML, Dohnt MF, et al. Optimization of culture of *Leptospira* from humans with leptospirosis. J Clin Microbiol. 2007;45:1363–5. PubMed http://dx.doi.org/10.1128/JCM.02430-06

7. Cole JR Jr, Sulzer CR, Pursell AR. Improved microtechnique for the leptospiral microscopic agglutination test. Appl Microbiol. 1973;25:976–80. PubMed

8. Syhavong B, Rasachack B, Smythe L, Rolain J-M, Roque-Afonso A-M, Jenjaroen K, et al. The infective causes of hepatitis and jaundice amongst hospitalised patients in Vientiane, Laos. Trans R Soc Trop Med Hyg. 2010;104:475–83. PubMed http://dx.doi.org/10.1016/j.trstmh.2010.03.002

9. Phetsouvanh R, Blacksell SD, Jenjaroen K, Day NPJ, Newton PN. Comparison of indirect immunofluorescence assays for diagnosis of scrub typhus and murine typhus using venous blood and finger prick filter paper blood spots. Am J Trop Med Hyg. 2009;80:837–40. PubMed http://dx.doi.org/10.4269/ajtmh.2009.80.837

10. Coleman RE, Sangkasuwan V, Suwanabun N, Eamsila C, Mungviriya S, Devine P, et al. Comparative evaluation of selected diagnostic assays for the detection of IgG and IgM antibody to *Orientia tsutsugamushi* in Thailand. Am J Trop Med Hyg. 2002;67:497–503. PubMed http://dx.doi.org/10.4269/ajtmh.2002.67.497

11. Moore CE, Blacksell SD, Taojaikong T, Jarman RG, Gibbons RV, Lee SJ, et al. A prospective assessment of the accuracy of commercial IgM ELISAs in diagnosis of Japanese encephalitis virus infections in patients with suspected central nervous system infections in Laos. Am J Trop Med Hyg. 2012;87:171–8. PubMed http://dx.doi.org/10.4269/ajtmh.2012.11-0729

12. Ikejima H, Haranaga S, Takemura H, Kamo T, Takahashi Y, Friedman H, et al. PCR-based method for isolation and detection of *Chlamydia pneumoniae* DNA in cerebrospinal fluids. Clin Diagn Lab Immunol. 2001;8:499–502. PubMed

13. Moore CE, Sengduangphachanh A, Thaojaikong T, Sirisouk J, Foster D, Phetsouvanh R, et al. Enhanced determination of *Streptococcus pneumoniae* serotypes associated with invasive disease in Laos by using a real-time polymerase chain reaction serotyping assay with cerebrospinal fluid. Am J Trop Med Hyg. 2010;83:451–7. PubMed http://dx.doi.org/10.4269/ajtmh.2010.10-0225

14. Ninove L, Nougairede A, Gazin C, Thirion L, Delogu I, Zandotti C, et al. RNA and DNA bacteriophages as molecular diagnosis controls in clinical virology: a comprehensive study of more than 45,000 routine PCR tests. PLoS One. 2011;6:e16142. PubMed http://dx.doi.org/10.1371/journal.pone.0016142

15. Thaipadungpanit J, Chierakul W, Wuthiekanun V, Limmathurotsakul D, Amornchai P, Boonslip S, et al. Diagnostic accuracy of real-time PCR assays targeting 16S rRNA and *lipL32* genes for human leptospirosis in Thailand: a case-control study. PLoS One. 2011;6:e16236. PubMed http://dx.doi.org/10.1371/journal.pone.0016236

16. Carvalho MG, Tondella ML, McCaustland K, Weidlich L, McGee L, Mayer LW, et al. Evaluation and improvement of real-time PCR assays targeting *lytA*, *ply*, and *psaA* genes for detection of pneumococcal DNA. J Clin Microbiol. 2007;45:2460–6. PubMed http://dx.doi.org/10.1128/JCM.02498-06

17. Corless CE, Guiver M, Borrow R, Edwards-Jones V, Fox AJ, Kaczmarski EB. Simultaneous detection of *Neisseria meningitidis*, *Haemophilus influenzae*, and *Streptococcus pneumoniae* in suspected cases of meningitis and septicemia using real-time PCR. J Clin Microbiol. 2001;39:1553–8. PubMed http://dx.doi.org/10.1128/JCM.39.4.1553-1558.2001

18. Mai NTH, Hoa NT, Nga TVT, Linh LD, Chau TTH, Sinh DX, et al. *Streptococcus suis* meningitis in adults in Vietnam. Clin Infect Dis. 2008;46:659–67. PubMed http://dx.doi.org/10.1086/527385

19. Ortika BD, Habib M, Dunne EM, Porter BD, Satzke C. Production of latex agglutination reagents for pneumococcal serotyping. BMC Res Notes. 2013;6:49. PubMed http://dx.doi.org/10.1186/1756-0500-6-49

20. Maaroufi Y, De Bruyne J-M, Heymans C, Crokaert F. Real-time PCR for determining capsular serotypes of *Haemophilus influenzae*. J Clin Microbiol. 2007;45:2305–8. PubMed http://dx.doi.org/10.1128/JCM.00102-07

21. Gray SJ, Trotter CL, Ramsay ME, Guiver M, Fox AJ, Borrow R, et al.; Meningococcal Reference Unit. Epidemiology of meningococcal disease in England and Wales 1993/94 to 2003/04: contribution and experiences of the Meningococcal Reference Unit. J Med Microbiol. 2006;55:887–96. PubMed http://dx.doi.org/10.1099/jmm.0.46288-0

22. McHugh MP, Gray SJ, Kaczmarski EB, Guiver M. Reduced turnaround time and improved diagnosis of invasive serogroup B *Neisseria meningitidis* and *Streptococcus pneumoniae* infections using a lyophilized quadruplex quantitative PCR. J Med Microbiol. 2015;64:1321–8. PubMed http://dx.doi.org/10.1099/jmm.0.000154

23. Jiang J, Chan T-C, Temenak JJ, Dasch GA, Ching W-M, Richards AL. Development of a quantitative real-time polymerase chain reaction assay specific for *Orientia tsutsugamushi*. Am J Trop Med Hyg. 2004;70:351–6. PubMed http://dx.doi.org/10.4269/ajtmh.2004.70.351

24. Horinouchi H, Murai K, Okayama A, Nagatomo Y, Tachibana N, Tsubouchi H. Genotypic identification of *Rickettsia tsutsugamushi* by restriction fragment length polymorphism analysis of DNA amplified by the polymerase chain reaction. Am J Trop Med Hyg. 1996;54:647–51. PubMed http://dx.doi.org/10.4269/ajtmh.1996.54.647

25. Jiang J, Stromdahl EY, Richards AL. Detection of *Rickettsia parkeri* and *Candidatus* Rickettsia andeanae in *Amblyomma maculatum* Gulf Coast ticks collected from humans in the United States. Vector Borne Zoonotic Dis. 2012;12:175–82. PubMed http://dx.doi.org/10.1089/vbz.2011.0614

26. Henry KM, Jiang J, Rozmajzl PJ, Azad AF, Macaluso KR, Richards AL. Development of quantitative real-time PCR assays to detect *Rickettsia typhi* and *Rickettsia felis*, the causative agents of murine typhus and flea-borne spotted fever. Mol Cell Probes. 2007;21:17–23. PubMed http://dx.doi.org/10.1016/j.mcp.2006.06.002

27. Kessler HH, Mühlbauer G, Rinner B, Stelzl E, Berger A, Dörr HW, et al. Detection of herpes simplex virus DNA by real-time PCR. J Clin Microbiol. 2000;38:2638–42. PubMed

28. Griscelli F, Barrois M, Chauvin S, Lastere S, Bellet D, Bourhis JH. Quantification of human cytomegalovirus DNA in bone marrow transplant recipients by real-time PCR. J Clin Microbiol. 2001;39:4362–9. PubMed http://dx.doi.org/10.1128/JCM.39.12.4362-4369.2001

29. Bousbia S, Papazian L, Saux P, Forel JM, Auffray J-P, Martin C, et al. Repertoire of intensive care unit pneumonia microbiota. PLoS One. 2012;7:e32486. PubMed http://dx.doi.org/10.1371/journal.pone.0032486

30. Lanciotti RS, Kerst AJ, Nasci RS, Godsey MS, Mitchell CJ, Savage HM, et al. Rapid detection of West Nile virus from human clinical specimens, field-collected mosquitoes, and avian samples by a TaqMan reverse transcriptase-PCR assay. J Clin Microbiol. 2000;38:4066–71. PubMed

31. Kaptein SJF, De Burghgraeve T, Froeyen M, Pastorino B, Alen MMF, Mondotte JA, et al. A derivate of the antibiotic doxorubicin is a selective inhibitor of dengue and yellow fever virus replication in vitro. Antimicrob Agents Chemother. 2010;54:5269–80. PubMed http://dx.doi.org/10.1128/AAC.00686-10

32. Watkins-Riedel T, Woegerbauer M, Hollemann D, Hufnagl P. Rapid diagnosis of enterovirus infections by real-time PCR on the LightCycler using the TaqMan format. Diagn Microbiol Infect Dis. 2002;42:99–105. PubMed http://dx.doi.org/10.1016/S0732-8893(01)00330-3

33. Leparc-Goffart I, Baragatti M, Temmam S, Tuiskunen A, Moureau G, Charrel R, et al. Development and validation of real-time one-step reverse transcription-PCR for the detection and typing of dengue viruses. J Clin Virol. 2009;45:61–6. PubMed http://dx.doi.org/10.1016/j.jcv.2009.02.010

34. Moureau G, Temmam S, Gonzalez JP, Charrel RN, Grard G, de Lamballerie X. A real-time RT-PCR method for the universal detection and identification of flaviviruses. Vector Borne Zoonotic Dis. 2007;7:467–77. PubMed http://dx.doi.org/10.1089/vbz.2007.0206

35. Moureau G, Ninove L, Izri A, Cook S, De Lamballerie X, Charrel RN. Flavivirus RNA in phlebotomine sandflies. Vector Borne Zoonotic Dis. 2010;10:195–7. PubMed http://dx.doi.org/10.1089/vbz.2008.0216

36. Hummel KB, Lowe L, Bellini WJ, Rota PA. Development of quantitative gene-specific real-time RT-PCR assays for the detection of measles virus in clinical specimens. J Virol Methods. 2006;132:166–73. PubMed http://dx.doi.org/10.1016/j.jviromet.2005.10.006

37. Uchida K, Shinohara M, Shimada S, Segawa Y, Doi R, Gotoh A, et al. Rapid and sensitive detection of mumps virus RNA directly from clinical samples by real-time PCR. J Med Virol. 2005;75:470–4. PubMed http://dx.doi.org/10.1002/jmv.20291

38. van Elden LJ, Nijhuis M, Schipper P, Schuurman R, van Loon AM. Simultaneous detection of influenza viruses A and B using real-time quantitative PCR. J Clin Microbiol. 2001;39:196–200. PubMed http://dx.doi.org/10.1128/JCM.39.1.196-200.2001

39. Weidmann M, Meyer-König U, Hufert FT. Rapid detection of herpes simplex virus and varicella-zoster virus infections by real-time PCR. J Clin Microbiol. 2003;41:1565–8. PubMed http://dx.doi.org/10.1128/JCM.41.4.1565-1568.2003

40. Nix WA, Oberste MS, Pallansch MA. Sensitive, seminested PCR amplification of VP1 sequences for direct identification of all enterovirus serotypes from original clinical specimens. J Clin Microbiol. 2006;44:2698–704. PubMed http://dx.doi.org/10.1128/JCM.00542-06

41. Zane RS, Daneshi A, Jenkins HA. Positional envelope as a response parameter in caloric testing. Acta Otolaryngol. 1991;111:639–45. PubMed http://dx.doi.org/10.3109/00016489109138394

42. Olsen SJ, Campbell AP, Supawat K, Liamsuwan S, Chotpitayasunondh T, Laptikulthum S, et al.; Thailand Encephalitis Surveillance Team. Infectious causes of encephalitis and meningoencephalitis in Thailand, 2003–2005. Emerg Infect Dis. 2015;21:280–9. PubMed http://dx.doi.org/10.3201/eid2102.140291

43. Ai J, Xie Z, Liu G, Chen Z, Yang Y, Li Y, et al. Etiology and prognosis of acute viral encephalitis and meningitis in Chinese children: a multicentre prospective study. BMC Infect Dis. 2017;17:494. PubMed http://dx.doi.org/10.1186/s12879-017-2572-9

44. Xie Y, Tan Y, Chongsuvivatwong V, Wu X, Bi F, Hadler SC, et al. A population-based acute meningitis and encephalitis syndromes surveillance in Guangxi, China, May 2007-June 2012. PLoS One. 2015;10:e0144366. PubMed http://dx.doi.org/10.1371/journal.pone.0144366

45. Tan V, Thai H, Phu NH, Nghia HDT, Chuong LV, Sinh DX, et al. Viral aetiology of central nervous system infections in adults admitted to a tertiary referral hospital in southern Vietnam over 12 years. PLoS Negl Trop Dis. 2014;8:e3127. PubMed http://dx.doi.org/10.1371/journal.pntd.0003127

46. Ho Dang Trung N, Le Thi Phuong T, Wolbers M, Nguyen Van Minh H, Nguyen Thanh V, Van MP, et al.; VIZIONS CNS Infection Network. Aetiologies of central nervous system infection in Viet Nam: a prospective provincial hospital-based descriptive surveillance study. PLoS One. 2012;7:e37825. PubMed http://dx.doi.org/10.1371/journal.pone.0037825

47. Taylor WR, Nguyen K, Nguyen D, Nguyen H, Horby P, Nguyen HL, et al. The spectrum of central nervous system infections in an adult referral hospital in Hanoi, Vietnam. PLoS One. 2012;7:e42099. PubMed http://dx.doi.org/10.1371/journal.pone.0042099

48. Wertheim HFL, Nguyen HN, Taylor W, Lien TTM, Ngo HT, Nguyen TQ, et al. *Streptococcus suis*, an important cause of adult bacterial meningitis in northern Vietnam. PLoS One. 2009;4:e5973. PubMed http://dx.doi.org/10.1371/journal.pone.0005973

49. Turner P, Suy K, Tan LV, Sar P, Miliya T, Hong NTT, et al. The aetiologies of central nervous system infections in hospitalised Cambodian children. BMC Infect Dis. 2017;17:806. PubMed http://dx.doi.org/10.1186/s12879-017-2915-6

50. Horwood PF, Duong V, Laurent D, Mey C, Sothy H, Santy K, et al. Aetiology of acute meningoencephalitis in Cambodian children, 2010–2013. Emerg Microbes Infect. 2017;6:e35. PubMed http://dx.doi.org/10.1038/emi.2017.15

51. Touch S, Hills S, Sokhal B, Samnang C, Sovann L, Khieu V, et al. Epidemiology and burden of disease from Japanese encephalitis in Cambodia: results from two years of sentinel surveillance. Trop Med Int Health. 2009;14:1365–73. PubMed http://dx.doi.org/10.1111/j.1365-3156.2009.02380.x

52. Srey VH, Sadones H, Ong S, Mam M, Yim C, Sor S, et al. Etiology of encephalitis syndrome among hospitalized children and adults in Takeo, Cambodia, 1999-2000. Am J Trop Med Hyg. 2002;66:200–7. PubMed http://dx.doi.org/10.4269/ajtmh.2002.66.200

53. Han S-H, Choi H-Y, Kim J-M, Park K-R, Youn YC, Shin H-W. Etiology of aseptic meningitis and clinical characteristics in immune-competent adults. J Med Virol. 2016;88:175–9. PubMed http://dx.doi.org/10.1002/jmv.24316

54. Phommasone K, Paris DH, Anantatat T, Castonguay-Vanier J, Keomany S, Souvannasing P, et al. Concurrent Infection with murine typhus and scrub typhus in southern Laos—the mixed and the unmixed. PLoS Negl Trop Dis. 2013;7:e2163. PubMed http://dx.doi.org/10.1371/journal.pntd.0002163

55. Ming DKY, Rattanavong S, Bharucha T, Sengvilaipaseuth O, Dubot-Pérès A, Newton PN, et al. *Angiostrongylus cantonensis* DNA in cerebrospinal fluid of persons with eosinophilic meningitis, Laos. Emerg Infect Dis. 2017;23:2112–3. PubMed http://dx.doi.org/10.3201/eid2312.171107

56. World Health Organization. Recommended standards for surveillance of selected vaccine-preventable diseases. 2003 May [cited 2018 Jun 6]. http://www.measlesrubellainitiative.org/wp-content/uploads/2013/06/WHO-surveillance-standard.pdf

57. Polage CR, Cohen SH. State-of-the-art microbiologic testing for community-acquired meningitis and encephalitis. J Clin Microbiol. 2016;54:1197–202. PubMed http://dx.doi.org/10.1128/JCM.00289-16

58. Venkatesan A, Tunkel AR, Bloch KC, Lauring AS, Sejvar J, Bitnun A, et al.; International Encephalitis Consortium. Case definitions, diagnostic algorithms, and priorities in encephalitis:

consensus statement of the international encephalitis consortium. Clin Infect Dis. 2013;57:1114–28. PubMed http://dx.doi.org/10.1093/cid/cit458

59. Tarantola A, Goutard F, Newton P, de Lamballerie X, Lortholary O, Cappelle J, et al. Estimating the burden of Japanese encephalitis virus and other encephalitides in countries of the mekong region. PLoS Negl Trop Dis. 2014;8:e2533. PubMed http://dx.doi.org/10.1371/journal.pntd.0002533

60. Glaser CA, Gilliam S, Schnurr D, Forghani B, Honarmand S, Khetsuriani N, et al. In search of encephalitis etiologies: diagnostic challenges in the California Encephalitis Project, 1998—2000. Clin Infect Dis. 2003;36:731–42. PubMed http://dx.doi.org/10.1086/367841

61. Glaser CA, Honarmand S, Anderson LJ, Schnurr DP, Forghani B, Cossen CK, et al. Beyond viruses: clinical profiles and etiologies associated with encephalitis. Clin Infect Dis. 2006;43:1565–77. PubMed http://dx.doi.org/10.1086/509330

62. Kolski H, Ford-Jones EL, Richardson S, Petric M, Nelson S, Jamieson F, et al. Etiology of acute childhood encephalitis at The Hospital for Sick Children, Toronto, 1994–1995. Clin Infect Dis. 1998;26:398–409. PubMed http://dx.doi.org/10.1086/516301

63. Kupila L, Vuorinen T, Vainionpää R, Hukkanen V, Marttila RJ, Kotilainen P. Etiology of aseptic meningitis and encephalitis in an adult population. Neurology. 2006;66:75–80. PubMed http://dx.doi.org/10.1212/01.wnl.0000191407.81333.00

64. Mailles A, Stahl J-P; Steering Committee and Investigators Group. Infectious encephalitis in France in 2007: a national prospective study. Clin Infect Dis. 2009;49:1838–47. PubMed http://dx.doi.org/10.1086/648419

65. Granerod J, Ambrose HE, Davies NW, Clewley JP, Walsh AL, Morgan D, et al.; UK Health Protection Agency Aetiology of Encephalitis Study Group. Causes of encephalitis and differences in their clinical presentations in England: a multicentre, population-based prospective study. Lancet Infect Dis. 2010;10:835–44. PubMed http://dx.doi.org/10.1016/S1473-3099(10)70222-X

Appendix Table 1. Etiologies of central nervous system infections in published prospective studies conducted in South-East Asia*

Study	Location	Study design	Clinical syndrome†	No. cases	Patients with confirmed diagnosis, no. (%)	Main etiologies, ≥2%, (%)	Mortality, no. (%)
Olsen et al. 2015 (*42*)	Thailand	Prospective study in 7 hospitals in Thailand, 2003–2005	Acute encephalitis syndrome	149	54 (36)	JEV (14), EV (4), O. tsu (4), Crypto (2), H. inf (2), S. pneu (2), EBV (2), M. pneu (2), Spot fev (2)	15 (10)
Ai et al. 2017 (*43*)	China	Multicenter prospective study in 5 hospitals, Beijing, Shandong, Shanxi, Gansu and Jiangsu province, from June 2009 to October 2012	Viral encephalitis and viral meningitis	546	259 (47.4)	EV (15.4), HSV1 (6.6), Mu (4), VZV (2.6)	2 (0.4)
Xie et al. 2015 (*44*)	China	Prospective study in 12 hospitals in China, 2007–2012	Acute meningitis and encephalitis	2,382	538 (<50)	EV (19), JEV (6), Mu (14), Bact (4), Me (3), HSV (3), Crypto (3)	75 (3)
Tan et al. 2014 (*45*)	Vietnam	Prospective study at Hospital for Tropical Diseases in Ho Chi Minh City, 1996–2008	CNS infections of viral origin suspected by physician, HIV negative, no evidence of purulent bacterial, eosinophilic, cryptococcal, or tuberculous meningitis by CSF cell count, culture, or microscopy	291	93 (32)	JEV (12), DENV (6.5), HSV (6.5), EV (3)	28 (10)
Ho Dang Trung et al. 2012 (*46*)	Vietnam	Prospective study in 13 hospitals, 2007 2010	Viral encephalitis and meningitis, bacterial meningitis	1,241 ‡	640 (52)	JEV (12), *S. suis* (12), S. pneu (6), EV (5), TB (4), H. inf (3), DENV (3), HSV (3),	115 (9)
Taylor et al. 2012 (*47*)	Vietnam	Prospective study from May 2007 to December 2008 at the National Hospital for Tropical Diseases (NHTD) in Hanoi	CNS infection upon judgment of admitting doctor	352	95 (27)	*S. suis* (14), HSV (3), TB (3), N. men (2)	21 (8)
Wertheim et al. 2009 (*48*)	Vietnam	Prospective study in adults at National Institute of Infectious and Tropical Diseases, Hanoi, January 2007 to December 2007	Suspected meningitis	562	68 (12)	*S. suis* (9), Crypto (2)	
Turner et al. 2017 (*49*)	Cambodia	Prospective study from September 2014 to October 2015 at Angkor Hospital associated Satellite Clinic (SC) at Sot Nikom District referral Hospital in Siem Reap for Children	Suspected CNS infection	284	55 (19.4)	EV (7.4), JEV (6.0), S. pneu (2.5).	(2.5)
Horwood et al. 2017 (*50*)	Cambodia	Prospective study from July 2010 to December 2013 at Kantha Bopha and Jayavarman VII, children hospitals in Phnom Penh and Siem Reap respectively	Acute meningoencephalitis	1160	406 (35)	JEV (24), O. tsu (5), DENV (5), EV (4), CHIKV (2), S. pneu (2)	
Touch et al. 2009 (*51*)	Cambodia	JEV sentinel surveillance in children in 6 hospitals, 2006 2008	Meningoencephalitis	586	110 (19)	JEV (19)	6 (10)
Srey et al. 2002 (*52*)	Cambodia	Prospective study in Takeo Provincial Hospital, October 1999 September 2000	Encephalitis syndrome	99	42 (42)	JEV (16), Crypto (7), TB (5), DENV (5), H. inf (3), Strep (2)	

Study	Location	Study design	Clinical syndrome†	No. cases	Patients with confirmed diagnosis, no. (%)	Main etiologies, ≥2%, (%)	Mortality, no. (%)
Han et al. 2016 (*53*)	Korea	Retrospective study in hospitalized adults, March 2008 to Feb 2013	Aseptic meningitis	177	96 (54)	EV (38), VZV (14)	

*In September 2016 we reviewed articles published in English in the Medline database in the past 15 years using the terms "encephalitis," "meningitis," "CNS syndrome" "CNS infection" "central nervous system syndrome" "central nervous system infection," with adding the terms "asia," or "south-east asia." Bact, bacteria; CHIKV, *Chikungunya virus*; Crypto, *Cryptococcus*; DENV, *Dengue virus*; EBV, Epstein-Barr virus; EV, *Enterovirus*, H. inf, *H. influenzae*; JEV, *Japanese encephalitis virus*; List, *Listeria monocytogenes*; Me, measles virus; M. pneu, *M. pneumoniae*; Mu, mumps *virus*; N. men, *N. meningitidis*; O. tsu, *O. tsutsugamushi*; S. pneu, *S. pneumoniae*; Spot fev, Spotted fever; TB, *M. tuberculosis*; TBE, *Tick-borne encephalitis virus*; Strep, *Streptococcus*; VZV, varicella zoster virus.
†Criteria for the definition of clinical syndromes are presented in Appendix Table 17, the article with no clear criteria for clinical syndromes definition are not in the Appendix Table 17.
‡Contrary to the other studies, after the inclusion of 1,645 patients with CNS presentation, 404 patients were excluded for unsuspected CNS infection.

Appendix Table 2. Demographic, clinical, blood and CSF parameters data at admission of all patients recruited in the study, with confirmed etiology, viral or bacterial infections*

	Age group			Etiology			
Characteristic or parameter	All, n = 1,065	<15 y, n = 358	≥15 y, n = 707	Confirmed, n = 450	None confirmed, n = 615	Viral, n = 172	Bacterial, n = 175
Demographic							
Male sex	666 (62.5)	207 (57.8)	459 (64.9)	288 (64.0)	378 (61.5)	111 (64.5)	117 (66.9)
Age, y, median (IQR)	23 (8–38)	3 (0.41–8)	32 (24–47)	23 (10–38)	24 (6–40)	16 (7–28)	23.0 (9–45)
Age group							
<1 mo	23 (2.2)	23 (6.4)	NA	4 (0.9)	19 (3.1)	2 (1.2)	2 (1.1)
1 mo–<1 y	112 (10.5)	112 (31.3)	NA	35 (7.8)	77 (12.5)	9 (5.2)	21 (12.0)
1–<5 y	73 (6.9)	73 (20.4)	NA	27 (6.0)	46 (7.5)	21 (12.2)	6 (3.4)
5–<15 y	150 (14.1)	150 (41.9)	NA	72 (16.0)	78 (12.7)	45 (26.2)	25 (14.3)
≥15 y	707 (66.4)	NA	707 (100)	312 (69.3)	395 (64.2)	95 (55.2)	121 (69.1)
Distance from hospital, n = 1,061, km, median (IQR)	25 (7–82)	29 (9–84)	20 (6–80)	28 (8–78)	23 (7–88)	39 (8–133)	27 (9–56)
Population density per km^2,† n = 1,051, median (IQR)	411 (92–1,949)	282 (73–1,567)	451 (100–2,027)	408 (92–1,686)	411 (91–2,027)	433 (70–1,821)	334 (92–1,285)
Occupation, n = 603							
Farmer	NA	NA	107 (17.7)	54 (20.2)	53 (15.8)	14 (17.7)	27 (27.3)
Work indoors	NA	NA	80 (13.3)	32 (12.0)	48 (14.3)	10 (12.7)	10 (10.1)
Work outdoors	NA	NA	151 (25.0)	71 (26.6)	80 (23.8)	16 (20.3)	23 (23.2)
Student	NA	NA	75 (12.4)	39 (14.6)	36 (10.7)	20 (25.3)	14 (14.1)
Other	NA	NA	190 (31.5)	71 (26.6)	119 (35.4)	18 (24.1)	25 (25.3)
History							
HIV seropositive, n = 703	119 (16.9)	1 (0.4)	118 (24.8)	75 (27.1)	44 (10.3)	8 (8.0)	6 (6.2)
Diabetic, n = 850	24 (2.8)	0	24 (4.2)	12 (3.5)	12 (2.4)	1 (0.8)	10 (7.5)
Tuberculosis, n = 734	35 (4.8)	1 (0.4)	34 (7.0)	18 (6.2)	17 (3.8)	3 (2.7)	2 (1.9)
Antibiotic use before lumbar puncture,‡ n = 953	590 (61.9)	238 (71.9)	352 (56.6)	252 (64.0)	338 (60.5)	109 (69.9)	100 (62.5)
Steroid use before LP, n = 854	58 (6.8)	26 (9.3)	32 (5.6)	21 (6.2)	37 (7.2)	9 (6.9)	7 (5.3)
Alcohol excess,§ n = 591	NA		249 (42.1)	106 (40.8)	143 (43.2)	29 (36.7)	44 (43.1)
Pet at home (dog cat), n = 585	523 (89.4)	172 (89.1)	351 (89.5)	218 (89.0)	305 (89.7)	81 (91.0)	90 (88.2)
Poultry at home, n = 539	481 (89.2)	174 (89.2)	307 (89.2)	203 (88.7)	278 (89.7)	86 (89.6)	81 (88.0)
Pigs at home, n = 416	346 (83.2)	102 (81.0)	244 (84.1)	163 (84.5)	183 (82.1)	70 (86.4)	54 (81.8)
Signs and symptoms							
Days of fever at admission, n = 1,058, median (IQR)	4 (2–8)	4 (2–6)	5 (2–10)	5 (3–10)	4 (1–7)	5 (3–7)	5 (3–8)
Fever, n = 1,059	962 (90.8)	340 (95.2)	622 (88.6)	425 (94.9)	537 (87.9)	162 (95.3)	171 (97.7)

	Age group			Etiology			
Characteristic or parameter	All, n = 1,065	<15 y, n = 358	≥15 y, n = 707	Confirmed, n = 450	None confirmed, n = 615	Viral, n = 172	Bacterial, n = 175
Headache,⁋ n = 893	787 (88.1)	155 (83.3)	632 (89.4)	369 (92.5)	418 (84.6)	139 (90.9)	135 (91.2)
Hearing loss,⁋ n = 893	51 (5.7)	10 (5.4)	41 (5.8)	20 (5.0)	31 (6.3)	8 (5.2)	7 (4.7)
Dysuria,⁋ n = 891	28 (3.1)	4 (2.2)	24 (3.4)	10 (2.5)	18 (3.7)	3 (2.0)	3 (2.0)
Visual loss,⁋ n = 885	66 (7.5)	14 (7.7)	52 (7.4)	23 (5.8)	43 (8.8)	11 (7.2)	5 (3.4)
Diplopia,⁋ n = 889	36 (4.1)	4 (2.2)	32 (4.5)	15 (3.4)	21 (4.3)	6 (4.0)	6 (4.1)
Photophobia, n = 850	52 (5.8)	14 (7.4)	38 (5.4)	23 (5.8)	29 (5.9)	7 (4.6)	10 (6.8)
Focal neurologic signs, n = 939	22# (2.3)	5 (1.6)	17 (2.7)	8 (2.1)	14 (2.5)	1 (0.7)	6 (4.1)
Neck stiffness, n = 1,064	683 (64.2)	245 (68.4)	438 (62.0)	316 (70.2)	367 (59.8)	130 (75.6)	128 (73.1)
Confusion, n = 1,060	608 (57.4)	232 (65.5)	376 (53.3)	254 (56.7)	354 (57.8)	114 (66.3)	103 (59.5)
Drowsiness, n = 1,059	611 (57.7)	234 (66.1)	377 (53.5)	268 (60.1)	343 (56.0)	111 (64.9)	110 (63.6)
Convulsions, n = 1,063	319 (30.0)	233 (65.3)	86 (12.2)	119 (26.5)	200 (32.6)	65 (37.8)	44 (25.3)
GCS score, n = 1,010, median (IQR)	14 (11–15)	14 (10–15)	15 (11–15)	15 (11–15)	14 (10–15)	13 (10–15)	14 (11–15)
GCS score <15,** n = 1,047	551 (52.6)	220 (63.4)	331 (47.3)	225 (50.5)	326 (54.2)	101 (59.4)	94 (54.0)
Arthralgia,⁋ n = 893	140 (15.7)	16 (8.6)	124 (17.5)	59 (14.8)	81 (16.4)	20 (13.1)	27 (18.3)
Myalgia,⁋ n = 893	419 (46.9)	55 (29.6)	364 (51.5)	186 (46.6)	233 (47.2)	72 (47.1)	75 (50.7)
Rash, n = 1,058	151 (14.3)	30 (8.5)	121 (17.2)	76 (17.0)	75 (12.3)	20 (11.7)	19 (10.9)
Vomiting or diarrhea, n = 1,064	575 (54.0)	236 (66.1)	339 (48.0)	257 (57.2)	318 (51.7)	101 (58.7)	101 (58.1)
Cough or shortness of breath, n = 1,064	338 (31.8)	135 (37.8)	203 (28.7)	142 (31.6)	196 (31.9)	47 (27.3)	50 (28.7)
Cough, n = 1,064	260 (24.4)	97 (27.2)	163 (23.1)	115 (25.6)	145 (23.6)	35 (20.4)	39 (22.4)
Shortness of breath, n = 1,064	155 (14.6)	75 (21.0)	80 (11.3)	54 (12.0)	101 (16.4)	20 (11.6)	23 (13.2)
Respiratory rate, n = 1,035, breaths/min, median (IQR)	22 (20–30)	32.5 (25.5–42)	21 (20–23)	22 (20–28)	22 (20–30)	24 (20–32)	23 (20–28)
WHO clinical CNS infection,†† n = 1,040	771 (74.1)	313 (90.7)	458 (65.9)	341 (77.0)	430 (72.0)	143 (85.1)	140 (80.9)
WHO encephalitis,†† n = 1,040	580 (55.8)	266 (77.1)	314 (45.2)	238 (53.7)	342 (57.3)	107 (63.7)	102 (59.0)
WHO meningitis,†† n = 1,040	742 (71.4)	290 (84.1)	452 (65.0)	335 (75.6)	407 (68.2)	140 (83.3)	138 (79.8)
WHO meningoencephalitis,†† n = 1,040	551 (53.0)	243 (70.4)	308 (44.3)	232 (52.4)	319 (53.4)	104 (61.9)	100 (57.8)
Fever + no neck stiffness + GCS score <15, seizures, or both, n = 1,040	127 (12.2)	78 (22.6)	49 (7.1)	37 (8.4)	90 (15.1)	17 (10.1)	16 (9.3)
Fever + neck stiffness + GCS score of 15 + no seizures, n = 1,040	191 (18.4)	47 (13.6)	144 (20.7)	103 (23.3)	88 (14.7)	36 (21.4)	38 (22.0)
Fever + neck stiffness + GCS score <15, seizures, or both, n = 1,040	453 (43.6)	188 (54.5)	265 (38.2)	201 (45.4)	252 (42.2)	90 (53.6)	86 (49.7)
Fever + neck stiffness, n = 1,040	644 (61.9)	235 (68.1)	409 (58.9)	304 (68.6)	340 (57.0)	126 (75.0)	124 (71.7)
Fever + GCS score <15, seizures, or both, n = 1,040	580 (55.8)	266 (77.1)	314 (45.2)	238 (53.7)	342 (57.3)	107 (63.7)	102 (59.0)
Peripheral blood analysis							
Total leukocyte count, n = 952, 10^3 cells/mm^3, median (IQR)	10.7 (7.6–14.5)	12 (8.4–16.9)	10.2 (7.2–13.8)	10.8 (7.3–15)	10.7 (7.9–14.2)	11.6 (8.6–14.5)	11.9 (8.2–16.4)
Elevated leukocyte count,‡‡ n = 952	449 (47.2)	150 (47.9)	299 (46.8)	198 (49.0)	251 (45.8)	84 (53.9)	84 (53.5)
Low white blow cell count,‡‡ n = 952	45 (4.7)	22 (7.0)	23 (3.6)	22 (5.5)	23 (4.2)	6 (3.9)	7 (4.5)
Hematocrit, n = 948, %, median (IQR)	38 (33–42)	36 (31–39)	39 (34–43)	38 (33–42)	38 (33–42)	39 (35–43.5)	37 (31.5–41)
Anemia,‡‡ n = 948	355 (37.5)	112 (35.7)	243 (38.3)	160 (39.8)	195 (35.7)	44 (28.2)	68 (43.9)
Platelets, n = 649, 10^3 count/mm^3, median (IQR)	218.1 (186–290)	230 (191–370.5)	210 (180–260)	220 (190–289)	210 (180–294)	220 (200–299)	220 (180–270)

	Age group			Etiology			
Characteristic or parameter	All, n = 1,065	<15 y, n = 358	≥15 y, n = 707	Confirmed, n = 450	None confirmed, n = 615	Viral, n = 172	Bacterial, n = 175
Thrombocytopenia,‡‡ n = 649	55 (8.5)	16 (6.8)	39 (9.4)	22 (7.8)	33 (9.0)	4 (3.5)	12 (10.6)
CRP, n = 868, mg/L, median (IQR)	20.2 (3.6–70.4)	9 (1.9–46.7)	24.5 (5.4–83.6)	25.4 (6.0–85.4)	14.2 (2.5–61.0)	19.2 (4.7–57.2)	64.4 (15.2–154.7)
Elevated CRP,‡‡ n = 868	547 (63.0)	145 (51.6)	402 (68.5)	265 (69.2)	282 (58.1)	98 (64.9)	114 (79.7)
Creatinine, n = 781, µmol/L, median (IQR)	79.6 (61.9–106.1)	53.0 (44.2–70.7)	88.4 (70.7–114.9)	79.6 (61.9–106.1)	79.6 (53.0–106.1)	70.7 (53.0–88.4)	79.6 (61.9–106.1)
Total bilirubin, n = 855, µmol/L, median (IQR)	5.3 (3.4–9.4)	5.1 (3.4–10.3)	5.5 (3.6–8.9)	5.8 (3.6–10.3)	5.1 (3.4–8.6)	5.1 (3.4–8.7)	6.8 (4.8–12.0)
ALP, n = 741, IU/L, median (IQR)	94 (66–156)	149 (101–217)	80 (61–126)	93 (66–145)	97 (66–161)	105 (74–144.5)	92.5 (69.5–161)
ALT, n = 831, IU/L, median (IQR)	17 (11–29)	16 (10–26)	17 (11–30)	17 (11–27)	17 (11–30)	14 (10–23)	18 (11–38)
AST, n = 843, IU/L, median (IQR)	46 (29–80)	47 (30–88)	45 (28–77)	45 (28–78)	46 (30–81)	44.5 (28–68)	48.5 (27–100)
Elevated serum sodium,§§ n = 807	225 (27.9)	45 (17.8)	180 (32.5)	82 (22.8)	143 (31.9)	40 (28.6)	26 (19.4)
Low serum sodium,§§ n = 807	63 (7.8)	31 (12.3)	32 (5.8)	31 (8.6)	32 (7.1)	8 (5.7)	16 (11.9)
Hyperglycemia,¶¶ n = 991	237 (23.9)	81 (25.8)	156 (23.0)	105 (24.5)	132 (23.5)	40 (24.0)	53 (32.3)
Severe hyperglycemia,¶¶ n = 991	72 (7.3)	26 (8.3)	46 (6.8)	35 (8.2)	37 (6.6)	12 (7.2)	22 (13.4)
CSF							
Turbid, n = 999	145 (14.5)	40 (12.2)	105 (15.7)	80 (18.4)	65 (11.5)	21 (12.4)	38 (23.2)
Hemorrhagic, n = 999	126 (12.6)	36 (11.0)	90 (13.4)	47 (10.8)	79 (14.0)	22 (13.0)	19 (11.6)
Xanthochromia, n = 999	44 (4.4)	7 (2.1)	37 (5.5)	20 (4.6)	24 (4.3)	5 (3.0)	11 (6.7)
Opening pressure, n = 977, H_2O cm, median (IQR)	20 (14–30)	19.8 (14–27)	20 (14–32)	21 (15.5–31)	18.5 (13.5–30)	20 (15–26.5)	20 (15.5–31.0)
Elevated opening pressure,‡‡ n = 977	334 (34.2)	86 (27.6)	248 (37.3)	155 (36.4)	179 (32.5)	42 (24.9)	60 (37.3)
Red cell count, n = 886, cells/mm^3, median (IQR)	0 (0–5)	0 (0–10)	0 (0–5)	0 (0–0)	0 (0–10)	0 (0–0)	0 (0–10)
Elevated red cell count,‡‡ n = 886	234 (26.4)	77 (27.2)	157 (26.0)	95 (24.0)	139 (28.4)	39 (24.5)	43 (28.7)
Total white cell count, n = 975, cells/mm^3, median (IQR)	40 (5–215)	35 (10–150)	40 (5–245)	65 (10–300)	20 (5–130)	82.5 (25–275)	115 (20–415)
Elevated white cell count,‡‡ n = 975	729 (74.8)	237 (74.8)	492 (74.8)	341 (80.2)	388 (70.6)	141 (84.9)	129 (80.1)
Lymphocytes, n = 890, %, median (IQR)	24.6 (0–64)	28 (0–63)	23.8 (0–64)	24 (0–61)	25 (0–66.7)	33.3 (2–71)	15.1 (0–40)
Elevated lymphocyte count,‡‡ n = 890	467 (52.5)	149 (51.2)	318 (53.1)	234 (59.5)	233 (46.9)	106 (68.4)	91 (62.3)
Neutrophils, n = 890, %, median (IQR)	50 (0–83)	50 (0–85)	49 (0–82.1)	56 (13–89)	41 (0–78)	48.4 (19–83)	70 (14.1–91)
Elevated neutrophil count,‡‡ n = 889	644 (72.4)	213 (73.5)	431 (72.0)	309 (78.8)	335 (67.4)	130 (83.9)	116 (80.0)
CSF eosinophilia, n = 1,001	46 (4.6)	7 (2.1)	39 (5.8)	11 (2.5)	35 (6.2)	9 (5.3)	2 (1.2)
Protein, n = 955, g/L, median (IQR)	0.56 (0.3–1.14)	0.48 (0.28–0.97)	0.64 (0.32–1.26)	0.69 (0.33–1.28)	0.52 (0.28–1.08)	0.65 (0.34–1.2)	0.8 (0.3–1.6)
Elevated protein,‡‡ n = 955	601 (62.9)	177 (57.3)	424 (65.6)	281 (66.9)	320 (59.8)	112 (66.3)	108 (69.7)
Glucose, n = 957, mmol/L, median (IQR)	3.56 (2.39–4.89)	3.89 (2.61–5.06)	3.44 (2.31–4.78)	3.33 (2.22–4.67)	3.83 (2.5–5.06)	3.56 (2.5–4.56)	3.4 (2.2–4.8)
Decreased glucose,‡‡ n = 957	280 (29.3)	58 (18.8)	222 (34.3)	138 (32.8)	142 (26.5)	45 (26.6)	51 (32.9)
Decreased CSF:venous glucose ratio,‡‡ n = 929	540 (58.1)	159 (54.8)	381 (59.6)	253 (61.7)	287 (55.3)	97 (58.8)	97 (64.2)
Lactate, n = 969, mmol/L, median (IQR)	2.7 (1.9–4.6)	2.8 (2–4.8)	2.7 (1.9–4.5)	3.1 (2–5.2)	2.6 (1.8–4.3)	2.3 (1.8–3.4)	4 (2.4–7.4)
Elevated lactate,‡‡ n = 985	650 (66.0)	217 (67.8)	433 (65.1)	298 (69.8)	352 (63.1)	93 (56.0)	132 (80.5)
Treatment after lumbar puncture							

Characteristic or parameter	Age group			Etiology			
	All, n = 1,065	<15 y, n = 358	≥15 y, n = 707	Confirmed, n = 450	None confirmed, n = 615	Viral, n = 172	Bacterial, n = 175
Antibiotic, n = 1,019	934 (91.7)	336 (96.6)	598 (89.1)	421 (95.9)	513 (88.5)	163 (97.0)	166 (96.5)
Steroid, n = 951	224 (23.6)	110 (33.4)	114 (18.3)	83 (20.4)	141 (25.9)	38 (24.2)	35 (21.1)
Outcome							
Hospitalization, n = 846, d, median (IQR)	9 (5–14)	8 (5–13)	10 (5–15.5)	11 (6–17)	8 (5–13)	10 (6–14)	11 (7–17)
Mortality,## n = 893	235 (26.3)	70 (22.5)	165 (28.4)	94 (25.0)	141 (27.3)	23 (15.7)	43 (27.9)
In hospital death, n = 893	124 (13.9)	40 (12.9)	84 (14.4)	53 (14.1)	71 (13.7)	12 (8.2)	24 (15.6)
Moribund, n = 893	111 (12.4)	30 (9.7)	81 (13.9)	41 (10.9)	70 (13.5)	11 (7.5)	19 (12.3)
Delay between admission and lumbar puncture, n = 1,022, d, median (IQR)	1 (0–3)	1 (0–1)	1 (0–3)	1 (0–2)	1 (0–3)	0 (0–2)	1 (0–2)

*Values are no. (%) except where indicated otherwise. Bacterial patients are those with confirmed bacterial infection, including patients with single bacterial infection (170) or with bacterial co-infection (5). Viral patients are those with confirmed viral infection, including patients with singe viral infection (169) or viral co-infection (3). ALP, alkaline phosphatase; ALT, alanine aminotransferase; AST, aspartate aminotransferase; CRP, C-reactive protein; CSF, cerebrospinal fluid; GCS, Glasgow coma scale; IQR, interquartile range; LP, lumbar puncture; NA, not applicable; TB, *M. tuberculosis*.

†Population density of the village of residence: Population densities per village were from population census 2005, recovered from Lao DECIDE info Web site (platform of Government of Lao PDR, www.decide.la). Occupation: work indoors = teacher, government official, business, factory worker, accountant; work outdoors = driver, building worker, merchant, carpenter, soldier, mechanic; other: housewife, no job, monk, retired, singer, health worker. History or physical examination were taken into account for: rash, confusion, neck stiffness, photophobia, fever (history of fever or >37.5°C during physical examination).

‡Antibiotics used before LP were: Ceftriaxone (47%), Ampicilin (17.5%), Gentamycin (11.5%), Doxycycline (8.0%), Amoxicillin (6.6%), Cefotaxime (5.9%), Penicillin (5.6%), Chloramphenicol (3.4%), Co-trimoxazole (3.1%), Ofloxacin (2.7%), Erythromicin (2.2%), Cloxacillin (1.7%), Metronidazole (1.4%), Co-amoxiclav (1.2%), Ceftazidime (0.5%), Anti tuberculosis (0.8%), Quinine (0.5%), Cefalexin (0.3%), Tetracycline (0.2%).

§Data collected for children (<15 years old) were excluded for analysis.

¶Considered as not reliable, the data were excluded from analysis for children <3 y old.

#Of these patients, 7 had hemiplegia, 11 had limb weakness, and 1 had paraplegia; 13 patients had admission or discharge diagnoses of Guillain-Barre syndrome. Retrospective evaluation of the likelihood of this diagnosis by using the Brighton system suggested that 4 patients met level 3 criteria for Guillain-Barre syndrome diagnostic certainty (Sejvar et al. 2011).

**Including confused and disoriented.

††WHO clinical CNS infection = fever with either GCS score <15, neck stiffness (history or examination), or history of seizure, patients with missing data for one of those criteria were not counted. WHO encephalitis = fever with either GCS score <15 or history of seizure. WHO meningitis = fever with GCS score <15 and/or neck stiffness. WHO meningoencephalitis = meeting both WHO encephalitis and WHO meningitis criteria.

‡‡Elevated and low parameters = above or below normal ranges (Appendix Table 3), anemia: hematocrit below normal range. In elevated CSF white cells count, were not taken into account the cases that could not be counted because of high turbidity. Eosinophilia = CSF eosinophils >10%.

§§Elevated serum sodium: higher than 150 mmol/L, low serum sodium: lower than <130 mmol/L. Five patients (0.6%) had serum sodium <115 mmol/L.

¶¶Hyperglycemia = blood glucose higher than 7.7 mmol/L, severe hyperglycemia: blood glucose higher than 11.1 mmol/L.

##Mortality includes patients who died at hospital and the ones who were taken to die at home = moribund.

Appendix Table 3. Reference values for normal ranges of CSF and blood parameters*

Parameter per demographic	Reference range	References
Blood parameters		
Total white cell count in blood, × 10^3 cells/µL		
M		Mayo Medical Laboratories (http://www.mayomedicallaboratories.com/test-catalog/Clinical+and+Interpretive/9109) (2015)
Birth	9.0–30.0	
1–7 d	9.4–34.0	
8–14 d	5.0–21.0	
15 d–1 mo	5.0–20.0	
2–5 mo	5.0–15.0	
6 mo–2 y	6.0–11.0	
2 y	5.0–12.0	
3–5 y	4.0–12.0	
6–11 y	3.4–9.5	
12–15 y	3.6–9	
Adults	3.5–10.5	
F		
Birth	9.0–30.0	
1–7 d	9.4–34.0	
8–14 d	5.0–21.0	
15 d–1 mo	5.0–20.0	
2–5 mo	5.0–15.0	
6 mo–2 y	6.0–11.0	
2 y	5.0–12.0	
3–5 y	4.0–12.0	
6–11 y	3.4–10.8	
12–15 y	4.1–8.9	
Adults	3.5–10.5	
Hemoglobin, g/dL		
M		Mayo Medical Laboratories (http://www.mayomedicallaboratories.com/test-catalog/Clinical+and+Interpretive/9109) (2015)
Birth–7 d	13.5–22.0	
8–14 d	12.5–21.0	
15 d–1 mo	10.0–20.0	
2–5 mo	10.0–14.0	
6 mo–2 y	10.5–13.5	
2 y	11.0–14.0	
3–5 y	11.0–14.5	
6–11 y	12.0–14.0	
12–15 y	12.8–16.0	
Adults	13.5–17.5	
F		
Birth–7 d	13.5–22.0	
8–14 d	12.5–21.0	
15 d–1 mo	10.0–20.0	
2–5 mo	10.0–14.0	
6 mo–2 y	10.5–13.5	
2 y	11.0–14.0	
3–5 y	11.8–14.7	
6–11 y	12.0–14.5	
12–15 y	12.2–14.8	
Adults	12.0–15.5	
Platelets, × 10^3/mm^3		
Birth–5 mo	150–350	Mayo Medical Laboratories (http://www.mayomedicallaboratories.com/test-catalog/Clinical+and+Interpretive/9109) (2015)
≥6 mo	150–450	
CRP, mg/L	<8	Mayo Medical Laboratories. (http://www.mayomedicallaboratories.com/test-catalog/Clinical+and+Interpretive/9109) (2016)
Cerebral spinal fluid		
Opening pressure, cm H_2O		
Birth–1 mo	<8	UK Standards for Microbiology Investigations. Issued by the Standards Unit, Microbiology Services, PHE. Bacteriology | B 27 | Issue no: 6 | Issue date: 24.02.15. No information for children between 1–3 mo., have been included in the 3 mo.–11 y old group, the neonate group being a very specific group
1 mo–11 y	12–28	
≥12 y	12–25	
Red cell count, cells/mm^3	0	
White cell count, cells/mm^3		
Birth–1 mo	0–30	UK Standards for Microbiology Investigations. Issued by the Standards Unit, Microbiology Services, PHE. Bacteriology | B 27 | Issue no: 6 | Issue date: 24.02.15.
1–3 mo	0–9	
3 mo–11 y	0–6	
≥12 y	0–5	
Lymphocyte count, cell/mm^3		
Birth–1 mo	<20	

Parameter per demographic	Reference range	References
>1 mo	≤5	The Royal Children's Hospital Melbourne (http://www.rch.org.au/clinicalguide/guideline_index/CSF_Interpretation/) (2015)
Neutrophil count, cells/mm^3	0	The Royal Children's Hospital Melbourne (http://www.rch.org.au/clinicalguide/guideline_index/CSF_Interpretation/) (2015)
Protein, g/L		
Birth–1 mo	<1	UK Standards for Microbiology Investigations. Issued by the Standards Unit, Microbiology Services, PHE. Bacteriology | B 27 | Issue no: 6 | Issue date: 24.02.15.
1–3 mo	0–0.09	
3 mo–11 y	0.05–0.4	
≥12 y	0.2–0.4	
Glucose, mmol/L		
Birth–1 mo	1.9–6.6	UK Standards for Microbiology Investigations. Issued by the Standards Unit, Microbiology Services, PHE. Bacteriology | B 27 | Issue no: 6 | Issue date: 24.02.15.
1 mo–11 y	2.2–4.4	
≥12 y	2.8–4.4	
CSF:venus glucose ratio		
Birth–1 mo	0.75–0.8	UK Standards for Microbiology Investigations. Issued by the Standards Unit, Microbiology Services, PHE. Bacteriology | B 27 | Issue no: 6 | Issue date: 24.02.15.No information for children between 1m–3m, have been included in the 3m–11 y old group, the neonate group being a very specific group
1 mo–11 y	≥0.6	
≥12 y	≥0.6	
Lactate, mmol/L	1.1–2.2	UK Standards for Microbiology Investigations. Issued by the Standards Unit, Microbiology Services, PHE. Bacteriology | B 27 | Issue no: 6 | Issue date: 24.02.15.

*CRP, C-reactive protein; CSF, cerebrospinal fluid; M, male; F, female.

Appendix Table 4. Pathogens detected in the 37 patients with confirmed co-infection*

Tissue	No. patients	Fist pathogen	Test	Second pathogen	Test	Third pathogen	Test
CSF							
Direct detection	1	HCMV	CSF PCR	*Streptococcus pneumoniae*	CSF PCR		
	1	*Dengue virus*	NS1 in CSF	*Rickettsia typhi*	CSF PCR		
	11	HCMV	CSF PCR	*Cryptococcus* sp.	CSF culture (1 Ag in CSF)		
	2	*Mycobacterium tuberculosis*	CSF culture	*Cryptococcus* sp.	2 CSF culture, 1 Ag in CSF		
	1	*R. typhi*	CSF PCR	HCMV	CSF PCR	VZV	CSF PCR
	2	*Haemophilus influenzae* type b	CSF PCR	HCMV	CSF PCR		
	1	*Cryptococcus* sp.	CSF indian ink	*R. typhi*	CSF PCR		
	1	VZV	CSF PCR	*Cryptococcus* sp.	CSF culture		
	1	*Rickettsia felis*	CSF PCR	HCMV	CSF PCR	*Cryptococcus* sp.	CSF culture
	2	*M. tuberculosis*	CSF culture	HCMV	CSF PCR		
	1	*Dengue virus*	NS1 in CSF	*S. pneumoniae*	CSF culture		
	1	HSV1/2	CSF PCR	*Cryptococcus* sp.	CSF culture		
	1	*Leptospira* sp.	CSF PCR	*M. tuberculosis*	CSF culture		
	1	HSV1/2	CSF PCR	*Cryptococcus* sp.	CSF culture	HCMV	CSF PCR
	1	HSV1/2	CSF PCR	HCMV	CSF PCR		
	1	*Streptococcus suis*	CSF culture	*R. typhi*	CSF PCR		
Indirect detection	1	JEV	IgM in CSF	Measles virus	IgM in CSF		
Blood							
Direct detection	1	*Dengue virus*	NS1 in serum	*Burkholderia pseudomallei*	Blood culture		
	1	*Dengue virus*	Serum PCR	*R. typhi*	Buffy coat PCR		

Tissue	No. patients	Fist pathogen	Test	Second pathogen	Test	Third pathogen	Test
	1	*Escherichia coli*	Blood culture	*Edwardsiella tarda*	Blood culture	*Leptospira* spp.	Buffy coat PCR
Indirect detection	2	*Orientia tsutsugamushi*	4× rise antibody	*Leptospira* spp.	4× rise antibody		
	1	*Dengue virus*	IgM seroconversion	Mumps virus	IgG seroconversion		
	1	*Dengue virus*	IgM seroconversion	*R. typhi*	4× rise antibody		

*Confirmed etiology was determined according to positive results by tests presented in Table 3, consisting of direct detection of the pathogen in CSF or serum or IgM detection in CSF, or antibody seroconversion between admission and follow-up serum. Based on Phommasone et al. (*54*), when >1 pathogen was detected in1 patient, the confirmed etiology was determined by giving the priority to direct detection over indirect detection and to CSF over blood. Confirmed co-infection was defined when >1 pathogens were detected in the same site (CSF or blood), both by direct tests, or both by indirect tests. Ag, antigen; CSF, cerebrospinal fluid; HCMV, human cytomegalovirus; HSV, herpes simplex virus; JEV, *Japanese encephalitis virus*; NS1, nonstructural protein 1; VZV, varicella zoster virus.

Appendix Table 5. List of pathogens detected in patients as single confirmed etiology*

Pathogen	No. patients	Sample site and diagnostic test: Cerebrospinal fluid	Sample site and diagnostic test: Blood
Japanese encephalitis virus, n = 94	81	IgM	
	4	PCR	
	1	Culture	
	8		IgM seroconversion
Cryptococcus gattii,† n = 9	9	Culture	
Cryptococcus neoformans, n = 42	42	Culture	
Cryptococcus spp., n = 19	4	Culture	
	4	India ink	
	11	Antigen LA‡	
Orientia tsutsugamushi, n = 31	21	PCR	
	1		Culture
	9		PCR
Dengue virus, n = 27	8	PCR	
	1	Nonstructural protein 1	
	5	IgM	
	4		PCR
	4		NS1
	5		IgM seroconversion
Leptospira spp., n = 25	5	PCR	
	1		Culture
	5		PCR
	14		4-fold antibody rise
Rickettsia typhi, n = 22	12	PCR	
	1		Culture
	2		PCR
	7		4-fold antigen rise
Rickettsia spp., n = 2	2		PCR
Streptococcus pneumoniae,§ n = 22	9	Culture	
	13	PCR	
Mycobacterium tuberculosis, n = 20	19	Culture	
	1	Ziehl-Neelson stain	
HSV, n = 15	8	HSV1 PCR	
	4	HSV2 PCR	
	3	HSV1/2 PCR	
Human cytomegalovirus, n = 12	12	PCR	
Enterovirus, n = 10	9	PCR	
	1		PCR
Varicella zoster virus, n = 6	6	PCR	

Pathogen	No. patients	Sample site and diagnostic test	
		Cerebrospinal fluid	Blood
Mumps virus, n = 5	2	PCR	
	3		IgG seroconversion
Plasmodium falciparum, n = 4	4		smear
Escherichia coli, n = 7	1	Culture	
	6		Culture
Streptococcus agalactiae, n = 4	2	Culture	
	2		Culture
Neisseria meningitides,⁋ n = 4	4	PCR	
Salmonella group D	1	Culture	
Salmonella group B or C	1	Culture	
Salmonella Typhi	5		Culture
Streptococcus suis, n = 4	3	Culture	
	1	PCR	
Klebsiella pneumoniae, n = 3	2	Culture	
	1		Culture
Haemophilus influenzae type b, n = 7	2	Culture	
	5	PCR	
Burkholderia pseudomallei, n = 5	5		Culture
Staphylococcus aureus, n = 6	1	Culture	
	5		Culture
Morganella morganii, n = 1	1	Culture	

*HSV, herpes simplex virus.
†1/6 *Cryptococcus gattii*, 31/33 *Cryptococcus neoformans*, 9/13 *Cryptococcus* spp. were from HIV-positive patients.
‡*Cryptococcus* Antigen Latex Agglutination Test System.
§*S. pneumoniae* serotypes: 1 (3 patients), 14 (2 patients), 18C (1 patient), 19A (1 patient), 19F (2 patients), 23B (1 patient), 23F (1 patient), 4 (1 patient), 5 (2 patients), 6 (1 patient), 6C (1 patient).
⁋*N. meningitides:* one serogroup B and 3 of undetermined serogroup.

Appendix Table 6. Susceptibility testing of bacteria cultured from CSF and/or blood using antibiotic disc diffusion and E tests*

Patient no.	Organism	Susceptible	Intermediate	Resistant to
42	Group B *Streptococcus*	Chloramphenicol, erythromycin, ofloxacin, penicillin		Trimsulpha
512	Group B *Streptococcus*	Chloramphenicol, erythromycin, ofloxacin, penicillin, vancomycin		
942	Group B *Streptococcus*	Chloramphenicol, erythromycin, ofloxacin, penicillin, vancomycin		
151	*Streptococcus pneumoniae*	Chloramphenicol	Erythromycin	Trimsulpha
233	*S. pneumoniae*	Ceftriaxone, penicillin, vancomycin	Erythromycin, ofloxacin	Chloramphenicol, trimsulpha
259	*S. pneumoniae*	Ceftriaxone, penicillin		Ofloxacin, trimsulpha
350	*S. pneumoniae*	Ceftriaxone, chloramphenicol, erythromycin, ofloxacin, vancomycin	Trimsulpha	Tetracycline, penicillin
374	*S. pneumoniae*	Erythromycin, penicillin	Ofloxacin	Chloramphenicol, trimsulpha
466	*S. pneumoniae*	Ceftriaxone, chloramphenicol, erythromycin, ofloxacin, penicillin, trimsulpha, vancomycin		
600	*S. pneumoniae*	Ceftriaxone, chloramphenicol, erythromycin, ofloxacin, penicillin, trimsulpha, vancomycin		
711	*S. pneumoniae*	Chloramphenicol, erythromycin, ofloxacin, vancomycin		Penicillin, trimsulpha
715	*S. pneumoniae*	Chloramphenicol, erythromycin, ofloxacin, trimsulpha, vancomycin		Penicillin
724	*S. pneumoniae*	Chloramphenicol, erythromycin, ofloxacin, penicillin, trimsulpha, vancomycin		

Patient no.	Organism	Susceptible	Intermediate	Resistant to
742	*S. pneumoniae*	Chloramphenicol, erythromycin, ofloxacin, penicillin, trimsulpha, vancomycin		
869	*S. pneumoniae*	Chloramphenicol, erythromycin, ofloxacin, penicillin, trimsulpha, vancomycin		
315	*Streptococcus suis*	Ceftriaxone, chloramphenicol, ofloxacin, penicillin, trimsulpha, vancomycin		Erythromycin, tetracycline
504	*S. suis*	Chloramphenicol, penicillin, vancomycin		Erythromycin
1,004	*S. suis*	Chloramphenicol, ofloxacin, penicillin, vancomycin		Erythromycin
1,055	*S. suis*	Ceftriaxone, chloramphenicol, ofloxacin, vancomycin		Erythromycin, tetracycline
65	*Staphylococcus aureus*	Cephalothin, erythromycin, gentamicin, methicillin, oxacillin, trimsulpha, cefoxitin		Penicillin, tetracycline
182	*S. aureus*	Cefoxitin, chloramphenicol, erythromycin, gentamicin, methicillin, oxacillin, tetracycline, trimsulpha		Penicillin
237	*S. aureus*	Cefoxitin, chloramphenicol, gentamicin, methicillin, oxacillin, penicillin, tetracycline, trimsulpha, vancomycin	Erythromycin	
190	*S. aureus*	Cefoxitin, erythromycin, gentamicin, methicillin, oxacillin, trimsulpha, tetracycline, vancomycin		Chloramphenicol, penicillin
757	*S. aureus*	Cefoxitin, gentamicin, oxacillin, trimsulpha, vancomycin		Erythromycin, penicillin, tetracycline
52	*S. aureus*	Cephalothin, cefoxitin, chloramphenicol, erythromycin, gentamicin, oxacillin, trimsulpha		Penicillin, tetracycline
81	*Burkholderia pseudomallei*	Augmentin, ceftazidime, chloramphenicol, ciprofloxacin, doxycycline, imipenem, oxacillin, trimsulpha		
810	*B. pseudomallei*	Augmentin, ceftazidime, chloramphenicol, ciprofloxacin, doxycycline, imipenem, trimsulpha		
941	*B. pseudomallei*	Augmentin, ceftazidime, ciprofloxacin, doxycycline, imipenem, trimsulpha		
993	*B. pseudomallei*	Augmentin, ceftazidime, ciprofloxacin, chloramphenicol, doxycycline, imipenem		Trimsulpha
1,032	*B. pseudomallei*	Augmentin, ceftazidime, ciprofloxacin, chloramphenicol, doxycycline, imipenem		Trimsulpha
1,065	*B. pseudomallei*	Augmentin, ceftazidime, chloramphenicol, ciprofloxacin, doxycycline, imipenem, trimsulpha		
257	*Salmonella* sp. group B or C	Ampicillin, ceftriaxone, chloramphenicol, trimsulpha	Nalidixic acid	
314	*Salmonella* group D	Ampicillin, ceftriaxone, chloramphenicol, nalidixic acid, ofloxacin, trimsulpha		
336	*Salmonella* Typhi	Missing data		
352	*S.* Typhi	Ampicillin, azithromycin, ceftriaxone, chloramphenicol, ciprofloxacin, nalidixic acid, ofloxacin, trimsulpha,		
592	*S.* Typhi	Ampicillin, azithromycin, ceftriaxone, chloramphenicol, nalidixic acid, ofloxacin, trimsulpha		
740	*S.* Typhi	Ampicillin, azithromycin, ceftriaxone, chloramphenicol, nalidixic acid, ofloxacin, trimsulpha		
340	*Klebsiella pneumoniae*	Augmentin, cephalothin, chloramphenicol, ceftriaxone, gentamicin, trimsulpha		Ampicillin
915	*K. pneumoniae*	Augmentin, chloramphenicol, gentamicin, imipenem		Ampicillin, cephalothin, ceftazidime, ceftriaxone, trimsulpha
1,041	*K. pneumoniae*	Augmentin, cephalothin, ceftriaxone, chloramphenicol, gentamicin, trimsulpha		Ampicillin
498	*Escherichia coli*	Augmentin, ceftriaxone, chloramphenicol, gentamicin	Cephalothin	Ampicillin, trimsulpha
593	*E. coli*	Augmentin, cephalothin, ceftriaxone, chloramphenicol, gentamicin		Ampicillin, trimsulpha
606	*E. coli*	Augmentin, cephalothin, ceftriaxone, chloramphenicol, gentamicin		Ampicillin, trimsulpha
623	*E. coli*	Ceftriaxone, chloramphenicol, gentamicin	Augmentin, cephalothin	Ampicillin, trimsulpha
733	*E. coli*	Ceftriaxone, chloramphenicol, gentamicin, trimsulpha	Augmentin	Ampicillin, cephalothin
891	*E. coli*	Ampicillin, augmentin, cephalothin, ceftriaxone, chloramphenicol, gentamicin		
934	*E. coli*	Ceftriaxone, chloramphenicol, gentamicin		Ampicillin, augmentin, cephalothin, trimsulpha
606	*Edwardsiella tarda*	Ampicillin, augmentin, cephalothin, ceftriaxone, chloramphenicol, gentamicin, ofloxacin, trimsulpha		

Patient no.	Organism	Susceptible	Intermediate	Resistant to
138	*Haemophilus influenzae*	Ceftriaxone, trimsulpha	Ampicillin	Chloramphenicol
722	*H. influenzae*	Ampicillin, ceftriaxone, chloramphenicol		
861	*H. influenzae*	Ceftriaxone		Ampicillin, chloramphenicol
851	*Morganella morganii*	Ceftriaxone, chloramphenicol, gentamicin, trimsulpha		Ampicillin, augmentin, cephalothin

**S. pneumoniae* with a penicillin MIC >0.06 or a ceftriaxone MIC >0.5 have been classified as resistant, according to Clinical and Laboratory Standards Institute guidelines. trimsulpha, trimethoprim/sulfamethoxazole.

Appendix Table 7. Demographic, clinical, blood, and CSF parameters data at admission of patients with confirmed etiology, for main etiologies (>20 patients)*

Characteristic or parameter	JEV, n = 94	*Dengue virus*, n = 27	*O. tsutsugamushi*, n = 31	*Leptospira* spp., n = 25	*Rickettsia* spp., n = 24	*S. pneumoniae*, n = 22	TB,† n = 20	*Cryptococcus* spp., n = 70
Demographic								
Male	55 (58.5)	22 (81.5)	22 (71.0)	17 (68.0)	17 (70.8)	13 (59.1)	14 (70.0)	40 (57.1)
Age, y, median (IQR)	13 (8–20)	20 (6–30)	16 (8–30)	25 (12–39)	31.5 (15–51)	17 (0.5–28)	35 (20–53)	33 (27–41)
<1 mo	0	1 (3.7)	0	0	0	0	0	0
1 mo–<1 y	0	2 (7.4)	2 (6.5)	2 (8.0)	2 (8.3)	7 (31.8)	0	0
1–<5 y	13 (13.8)	3 (11.1)	0	1 (4.0)	1 (4.2)	0	0	0
5–<15 y	37 (39.4)	2 (7.4)	12 (38.7)	4 (16.0)	3 (12.5)	3 (13.6)	0	0
≥15 y	44 (46.8)	19 (70.4)	17 (54.8)	18 (72.0)	18 (75.0)	12 (54.6)	20 (100)	70 (100)
Distance from hospital, km, median (IQR)	75 (15–155)	12 (4–54)	19 (9–46)	36 (13–154)	28 (7–58)	23 (8–50)	16 (6–124)	13 (6–53)
Population density per km^2,‡ median (IQR)	163 (31–1,371)	1,346 (173–2,510)	295 (109–1,228)	326 (63–741)	262 (98–767)	403 (101–1,963)	421 (156–1,982)	563 (173–1,686)
Occupation,§ n = 78								
Farmer	7 (17.5)	1 (8.3)	3 (21.4)	6 (37.5)	2 (13.3)	3 (30.0)	5 (33.3)	10 (15.9)
Work indoors	4 (10.0)	3 (25.0)	1 (7.1)	1 (6.3)	2 (13.3)	0	2 (13.3)	8 (12.7)
Work outdoors	3 (7.5)	4 (33.3)	3 (21.4)	3 (18.8)	5 (33.3)	3 (30.0)	4 (26.7)	20 (31.8)
Student	15 (37.5)	3 (25.0)	5 (35.7)	1 (6.3)	1 (6.7)	1 (10.0)	2 (13.3)	3 (4.8)
Other	11 (27.5)	1 (8.3)	2 (14.3)	5 (31.3)	5 (31.3)	3 (30.0)	2 (13.3)	22 (34.9)
History								
HIV seropositive	0	1 (5.6)	1 (5.6)	0	0	0	1 (12.5)	41 (78.9)
Diabetic	0	0	0	1 (5.9)	2 (11.8)	0	1 (7.1)	1 (1.7)
Tuberculosis	0	1 (4.8)	1 (4.6)	0	0	0	1 (10.0)	6 (12.8)
Antibiotic use before LP	70 (80.5)	18 (75.0)	24 (85.7)	15 (65.2)	11 (52.4)	13 (65.0)	11 (61.1)	28 (50.9)
Steroid use before LP	4 (5.8)	1 (4.8)	1 (3.9)	0	0	1 (7.1)	3 (20.0)	3 (5.8)
Alcohol excess¶	10 (25.6)	8 (47.1)	4 (36.4)	4 (26.7)	10 (58.8)	7 (70.0)	5 (29.4)	25 (43.1)
Pet at home (dog cat)	50 (100)	13 (81.3)	17 (85.0)	13 (92.9)	11 (100)	12 (85.7)	9 (90.0)	31 (86.1)
Poultry at home	56 (100)	12 (80.0)	12 (80.0)	13 (100)	11 (91.7)	15 (88.2)	7 (100)	26 (86.7)
Pigs at home	44 (95.7)	7 (70.0)	6 (66.7)	12 (100)	7 (87.5)	6 (66.7)	6 (100)	25 (80.7)
Signs and symptoms								
Days of fever at admission, median (IQR)	5 (3–7)	4.5 (3–7)	6.5 (4–8)	4 (3–6)	4 (2.5–7)	2 (1–4)	10 (6–14)	7 (1–21)
Fever	92 (97.9)	24 (92.3)	31 (100)	25 (100)	24 (100)	22 (100)	19 (95.0)	60 (85.7)
Headache#	82 (91.1)	20 (87.0)	25 (89.3)	22 (95.7)	21 (95.5)	13 (86.7)	19 (95.0)	67 (95.7)
Neck stiffness	82 (87.2)	18 (66.7)	23 (74.2)	17 (68.0)	17 (70.8)	18 (81.8)	17 (85.0)	38 (54.3)
Confusion	74 (78.7)	18 (66.7)	11 (37.9)	12 (48.0)	16 (66.7)	17 (77.3)	15 (75.0)	24 (34.3)
Drowsiness	72 (76.6)	14 (51.9)	19 (65.5)	14 (56.0)	17 (70.8)	14 (63.6)	14 (70.0)	32 (46.4)
Convulsions	40 (42.6)	9 (33.3)	7 (22.6)	5 (20.0)	4 (16.7)	10 (47.6)	2 (10.0)	2 (2.9)
GCS score, median (IQR)	13 (9.5–15)	13 (10–15)	15 (14–15)	15 (10–15)	14 (11–15)	11 (10–14)	11.5 (9–14)	15 (14–15)
GCS score <15**	68 (72.3)	17 (63.0)	10 (32.3)	12 (48.0)	14 (58.3)	17 (77.3)	15 (75.0)	19 (27.5)
Arthralgia#	7 (7.8)	5 (21.7)	4 (14.3)	2 (8.7)	3 (13.6)	3 (20.0)	4 (20.0)	9 (12.9)
Myalgia#	44 (48.9)	13 (56.5)	15 (53.6)	11 (47.8)	10 (45.5)	6 (40.0)	9 (45.0)	28 (40.0)
Rash	8 (8.5)	5 (18.5)	7 (23.3)	2 (8.0)	1 (4.2)	1 (4.6)	2 (10.0)	24 (34.8)
Vomiting or diarrhea	56 (59.6)	16 (59.3)	20 (66.7)	15 (60.0)	11 (45.8)	11 (50.0)	10 (50.0)	36 (51.4)
Cough	20 (21.3)	7 (25.9)	5 (16.7)	4 (16.0)	5 (20.8)	4 (18.2)	6 (30.0)	29 (41.4)
Shortness of breath	11 (11.7)	2 (7.4)	2 (6.7)	4 (16.0)	2 (8.3)	6 (27.3)	1 (5.0)	8 (11.4)

Characteristic or parameter	JEV, n = 94	*Dengue virus*, n = 27	*O. tsutsugamushi*, n = 31	*Leptospira* spp., n = 25	*Rickettsia* spp., n = 24	*S. pneumoniae*, n = 22	TB,† n = 20	*Cryptococcus* spp., n = 70
Cough or shortness of breath	26 (27.7)	8 (29.6)	5 (16.7)	7 (28.0)	6 (25.0)	6 (27.3)	6 (30.0)	31 (44.3)
Respiratory rate, breaths/min, median (IQR)	24 (21–32)	22 (20–32)	23 (20–27)	23 (20–26)	22 (20–26)	23.5 (20–40)	22 (20–23)	20 (20–22)
WHO clinical CNS infection††	89 (94.7)	21 (80.8)	26 (83.9)	19 (76.0)	18 (75.0)	21 (100)	17 (85.0)	36 (52.2)
WHO encephalitis††	74 (78.7)	16 (61.5)	13 (41.9)	13 (52.0)	15 (62.5)	18 (85.7)	15 (75.0)	16 (23.2)
WHO meningitis††	88 (93.6)	21 (80.8)	25 (80.7)	19 (76.0)	18 (75.0)	21 (100)	17 (85.0)	36 (52.2)
WHO meningoencephalitis††	73 (77.7)	16 (61.5)	12 (38.7)	13 (52.0)	15 (62.5)	18 (85.7)	15 (75.0)	16 (23.2)
Fever + no neck stiffness + GCS score <15 and/or seizures	7 (7.5)	5 (19.2)	3 (9.7)	2 (8.0)	1 (4.2)	4 (19.1)	1 (5.0)	1 (1.5)
Fever + neck stiffness + GCS score of 15 + no seizures	15 (16.0)	5 (19.2)	13 (41.9)	6 (24.0)	3 (12.5)	3 (14.3)	2 (10.0)	20 (29.0)
Fever + neck stiffness + GCS score <15 and/or seizures	67 (71.3)	11 (42.3)	10 (32.3)	11 (44.0)	14 (58.3)	14 (66.7)	14 (70.0)	15 (21.7)
Fever + neck stiffness	82 (87.2)	16 (61.5)	23 (74.2)	17 (68.0)	17 (70.8)	17 (81.0)	16 (80.0)	35 (50.7)
Fever + GCS score <15 and/or seizures	74 (78.7)	16 (61.5)	13 (41.9)	13 (52.0)	15 (62.5)	18 (85.7)	15 (75.0)	16 (23.2)
Peripheral blood analysis								
Total leukocyte count, 10^3 cells/mm^3, median (IQR)	12.3 (8.8–16.2)	9.8 (6.9–13)	12.1 (9.4–14.0)	11.3 (8.5–16)	8.8 (6.6–13.2)	15 (9.2–18.0)	11.5 (7.1–14.4)	8 (5.4–12.3)
Elevated white cell count‡‡	55 (64.7)	8 (32.0)	18 (69.2)	10 (45.5)	9 (37.5)	11 (57.9)	10 (55.6)	21 (33.9)
Low white cell count‡‡	2 (2.4)	3 (12.0)	1 (3.9)	1 (4.6)	0	2 (10.5)	0	8 (12.9)
Hematocrit (%), median (IQR)	38.1 (35.1–43)	39.3 (36.1–43)	38.4 (35.6–40.8)	38.5 (33–41)	35.9 (31–42)	36 (30–41)	36.7 (33–43)	38 (32–42)
Anemia‡‡	23 (27.1)	6 (24.0)	10 (38.5)	8 (36.4)	13 (54.2)	8 (42.1)	8 (47.1)	31 (50.0)
Platelet, 10^3 count/mm^3, median (IQR)	218 (190–265)	270 (200–346)	210 (180–229)	220 (180–260)	220 (190–280)	297 (190–389)	271 (204–368.5)	230 (200–319)
Thrombocytopenia‡‡	2 (2.9)	1 (5.6)	3 (13.6)	2 (11.1)	1 (5.3)	0	2 (16.7)	2 (5.4)
CRP, mg/L, median (IQR)	27.6 (6.1–66.7)	8.6 (2.5–33.8)	43.4 (21.1–118.6)	98.4 (39.4–156.8)	15.4 (5.1–87.7)	153.3 (38.2–205)	5.9 (2.4–96.4)	21.2 (5.4–44.1)
Elevated CRP‡‡	58 (69.9)	12 (50.0)	23 (95.8)	15 (83.3)	10 (52.6)	19 (100)	9 (47.4)	42 (65.6)
Creatinine, µmol/L, median (IQR)	70.7 (53.0–88.4)	70.7 (61.9–141.4)	70.7 (53.0–97.2)	88.4 (70.7–123.8)	79.6 (70.7–106.1)	70.7 (44.2–106.1)	88.4 (70.7–114.9)	79.6 (61.9–1,061)
Total bilirubin, µmol/L, median (IQR)	5.1 (3.4–8.6)	5.1 (3.4–10.3)	6.8 (5.0–10.3)	7.5 (5.1–11.3)	6.8 (5.0–11.1)	8.6 (4.1–12.0)	5.1 (4.3–10.3)	5.5 (3.4–7.5)
ALP, IU/L, median (IQR)	115 (76–144)	119 (86–145)	112 (81–249)	101 (78–182)	83 (75–141)	81 (70–94)	72 (60–116)	76 (59–110)
ALT, IU/L, median (IQR)	14 (10–23)	18.5 (11–25)	30 (18–70)	15 (11–23)	16 (9–36)	19 (10–26)	13 (8–24)	18 (12–28)
AST, IU/L, median (IQR)	49 (30–74.5)	44 (32–59)	72 (36–175)	34 (23–79)	34 (26–85)	62 (35–100)	28 (20–55)	42 (29–65)
Hyperglycemia§§	27 (28.7)	7 (28.0)	5 (19.2)	7 (29.2)	6 (25.0)	10 (47.6)	6 (30.0)	8 (11.9)
Severe hyperglycemia§§	10 (10.6)	1 (4.0)	2 (7.7)	2 (8.3)	1 (4.2)	4 (19.1)	1 (5.0)	1 (1.5)
CSF								
Turbid	8 (8.7)	3 (11.1)	4 (16.0)	3 (12.5)	0	13 (61.9)	1 (5.0)	13 (19.1)
Hemorrhagic	3 (3.3)	6 (22.2)	2 (8.0)	2 (8.3)	3 (13.6)	2 (9.5)	2 (10.0)	4 (5.9)
Xanthochromia	0	2 (7.4)	2 (8.0)	0	1 (4.6)	0	2 (10.0)	1 (1.5)
Opening pressure, H_2O cm, median (IQR)	20 (15.5–24.5)	19 (15–27)	21 (18–29)	20 (17–27)	17.5 (13.5–25.5)	24 (12–35)	30.5 (19–40.5)	29 (18–40)
Elevated opening pressure,‡‡	14 (15.1)	9 (33.3)	8 (30.8)	8 (34.8)	6 (25.0)	7 (36.8)	12 (60.0)	39 (59.1)

Characteristic or parameter	JEV, n = 94	*Dengue virus*, n = 27	*O. tsutsugamushi*, n = 31	*Leptospira* spp., n = 25	*Rickettsia* spp., n = 24	*S. pneumoniae*, n = 22	TB,† n = 20	*Cryptococcus* spp., n = 70
Red cell count, cells/ mm^3, median (IQR)	0 (0–0)	0 (0–0)	0 (0–5)	0 (0–0)	0 (0–5)	0 (0–160)	0 (0–0)	0 (0–0)
Elevated red cell count‡‡	16 (17.8)	5 (21.7)	6 (27.3)	5 (21.7)	4 (25.0)	9 (42.9)	4 (20.0)	12 (19.1)
Total white cell count (cells/mm^3), median (IQR)	82.5 (30–275)	30 (0–155)	107.5 (50–230)	60 (5–357.5)	10 (0–85)	400 (167.5–1,140)	155 (55–440)	20 (7.5–75)
Elevated white cell count‡‡	85 (90.4)	14 (56.0)	21 (80.8)	17 (70.8)	13 (59.1)	20 (100)	18 (90.0)	51 (75.0)
Lymphocytes, %, median (IQR)	47.7 (11–71)	3 (0–50)	20 (0–36)	22 (0–59.5)	10.5 (0–50)	11.5 (2–30)	28 (6.5–73.5)	27.5 (0–52.4)
Elevated lymphocyte count‡‡	63 (73.3)	9 (37.5)	13 (68.4)	14 (58.3)	6 (30.0)	15 (83.3)	16 (80.0)	28 (42.4)
Neutrophils, %, median (IQR)	48.7 (23–82)	32 (0–71.5)	70 (20–95)	49 (0.5–78)	48.5 (0–78)	88.5 (70–98)	66.5 (11.6–82.5)	50 (0–84)
Elevated neutrophil count‡‡	76 (88.4)	14 (58.3)	17 (89.5)	18 (75.0)	12 (60.0)	18 (100)	16 (80.0)	49 (74.2)
CSF eosinophilia¶¶	2 (2.2)	3 (11.1)	0	1 (4.2)	1 (4.2)	0	0	0
Protein, g/L, median (IQR)	0.62 (0.34–0.98)	0.72 (0.37–1.4)	0.7 (0.4–1.5)	0.3 (0.3–0.9)	0.7 (0.3–1.3)	1.6 (0.6–5.5)	1.1 (0.4–2.3)	0.51 (0.31–0.9)
Elevated protein‡‡	61 (64.9)	17 (65.4)	16 (72.7)	11 (52.4)	11 (52.4)	19 (86.4)	16 (80.0)	40 (61.5)
Glucose, mmol/L, median (IQR)	3.7 (2.8–4.6)	3.7 (2.7–5.5)	3.8 (2.9–5.3)	4.2 (3.6–5)	3.3 (2.7–4.7)	2.5 (1.8–4.2)	2.2 (1.5–3.3)	2.7 (1.8–4.2)
Decreased glucose‡‡	19 (20.2)	7 (26.9)	4 (18.2)	3 (14.3)	6 (28.6)	11 (50.0)	13 (65.0)	34 (51.5)
Decreased CSF:venus glucose ratio‡‡	51 (54.3)	13 (54.2)	12 (57.1)	8 (38.1)	12 (57.1)	17 (81.0)	18 (90.0)	41 (64.1)
Lactate, mmol/L, median (IQR)	2.1 (1.6–3)	2.8 (1.8–5.2)	3 (2.5–3.9)	2.8 (2.0–5.0)	2.5 (1.7–5)	11.6 (4.9–19.0)	6.9 (5.4–7.6)	3.1 (1.9–4.7)
Elevated lactate‡‡	43 (47.8)	15 (55.6)	21 (80.8)	17 (70.8)	17 (70.8)	17 (85.0)	20 (100)	48 (71.6)
Treatment post LP								
Antibiotic	92 (98.9)	26 (96.3)	29 (100)	25 (100)	21 (87.5)	21 (95.5)	19 (95.0)	61 (91.0)
Steroid	21 (23.6)	6 (24.0)	4 (14.8)	3 (12.0)	2 (8.7)	6 (27.3)	8 (42.1)	7 (12.3)
Outcome								
Days of hospitalization, median (IQR)	10 (8–14)	10 (6–15)	8 (5–12)	9.5 (5–16)	9 (3–13)	13 (10–17)	11 (8–26)	18 (5–26.5)
Mortality and discharge moribund	11 (12.9)	5 (20.0)	3 (12.0)	3 (13.6)	6 (26.1)	8 (36.4)	10 (58.8)	20 (37.7)
Delay between admission and lumbar puncture, d, median (IQR)	0 (0–1)	0 (0–1)	1 (0–3)	0.5 (0–2)	1 (0–5)	1 (0–1)	0 (0–5)	2 (0–5)

Characteristic or parameter	JEV, n = 94	*Dengue virus*, n = 27	*O. tsutsugamushi*, n = 31	*Leptospira* spp., n = 25	*Rickettsia* spp., n = 24	*S. pneumoniae*, n = 22	TB,† n = 20	*Cryptococcus* spp., n = 70

*Values are no. (%), except where stated otherwise. History or physical examination were taken into account for rash, confusion, neck stiffness, fever (history of fever or >37.5°C during physical examination). Described in the table are the patients with single confirmed etiology, for etiology detected in >20 patients. A complete list of single confirmed etiologies is provided in Appendix Table 5. Confirmed etiology was determined according to positive results by the tests presented in Table 3, consisting in direct detection of the pathogen in CSF or blood, IgM detection in CSF, antibody seroconversion or 4-fold rise in antibody titter between admission and follow-up serum. When >1 pathogens were detected in a same patient, the confirmed etiology was determined by giving the priority to direct detection over indirect detection then to CSF over blood. Confirmed co-infection was defined when > one pathogens were detected by the same kind of test in the same matrix. List of confirmed co-infections in supplemental data (Appendix Table 4). The other etiologies confirmed in <20 patients were cytomegalovirus in 12 patients, herpes simplex virus in 15, *Enterovirus* in 10, varicella zoster virus in 6, mumps virus in 5, *Plasmodium falciparum* in 4, and other bacteria in 48 patients (the list of bacteria is provided in Appendix Table 5). Among 35 patients with CSF eosinophils >10%, 4 were found positive for *Angiostrongylus cantonensis* by PCR (*55*). Among 662 patients tested for syphilis by the SD. Bioline RDT (Cat No. 06FK10) on serum then confirmed by VDRL and TPHA on serum and CSF, 2 patients could be classified as possible neurosyphilis, as per the UK and European guidelines (TPHA positive in CSF). Other bacterial antibiotic susceptibility data are given in Appendix Table 6. Typing information for *Cryptococcus* spp. is presented in Appendix Table 5. ALP, alkaline phosphatase; ALT, alanine transaminase; AST, aspartate transaminase; CNS, central nervous system; CRP, C-reactive protein; CSF, cerebrospinal fluid; GCS, Glasgow coma scale; IQR, interquartile range; JEV, *Japanese encephalitis virus*; LP, lumbar puncture; TB, *M. tuberculosis*; WHO, world health organization.
†Nine *Mycobacterium tuberculosis* were sensitive to isoniazid (0.1 µg/mL, 0.4 µg/mL for one), rifampin (1.0 µg/mL), streptomycin (1.0 µg/mL), ethambutol (5.0 µg/mL), and pyrazinamide (100.0 µg/mL). Two were sensitive to rifampin (1.0 µg/mL), ethambutol (5.0 µg/mL), and pyrazinamide (100.0 µg/mL) and resistant to isoniazid (0.4 µg/mL) and streptomycin (1.0 µg/mL). Three were sensitive to isoniazid (0.1 µg/mL), rifampin (1.0 µg/mL), ethambutol (5.0 µg/mL), and pyrazinamide (100.0 µg/mL) and resistant to streptomycin (1.0 µg/mL). For 1 patient only the test for isoniazid and rifampin could be performed, *M. tuberculosis* was sensitive for both. Susceptibility testing could not be performed for 4 patients.
‡Population density of the village of residence: Population densities per village were from population census 2005, recovered from Lao DECIDE info Web site (platform of Government of Lao PDR, www.decide.la).
§Occupation classification: work indoors = teacher, government official, business, factory worker, accountant; work outdoors = driver, building worker, merchant, carpenter, soldier, mechanic; other = housewife, no job, monk, retired, singer, health worker.
¶Data collected for children (<15 y old) were excluded for analysis.
#Considered as not reliable, the data were excluded from analysis for children <3 years old.
**Including confused and disoriented.
††WHO clinical CNS infection= fever with either GCS score <15, neck stiffness (history or examination), or history of seizure, patients with missing data for 1 of those criteria were not counted. WHO encephalitis = fever with GCS score <15 or history of seizure or both. WHO meningitis = fever with GCS score <15 or neck stiffness or both. WHO meningoencephalitis = meeting both WHO encephalitis and WHO meningitis criteria.
‡‡Elevated and decreased parameters = above or below normal ranges (Appendix Table 3), anemia: hematocrit below normal range. In elevated CSF white cell count, were not taken into account the cases that could not be counted because of high turbidity.
§§Hyperglycemia: blood glucose higher than 7.7 mmol/L, severe hyperglycemia: blood glucose higher than 11.1 mmol/L.
¶¶CSF eosinophils >10%.

Appendix Table 8. Comparison of etiology distribution according to age*

Etiologic agent	Proportion of group with etiology, no. (%) Children	Adult	p value	All patients	Proportion of total with etiology, no. (%) Children	Adult	Age, y, median (IQR)
Overall	n = 358	n = 707		n = 1,065	358 (33.6)	707 (66.4)	23 (8–38)
Confirmed etiology	138 (38.6)	312 (44.1)	0.086	450 (42.3)	138/450 (30.7)	312 (69.3)	23 (10–38)
Co-infection	8 (2.2)	29 (4.1)	0.109	37 (3.5)	8 (21.6)	29 (78.4)	29 (22–33)
Orientia tsutsugamushi	14 (3.9)	17 (2.4)	0.168	31 (2.9)	14 (45.2)	17 (54.8)	16 (8–30)
Leptospira sp.	7 (2.0)	18 (2.5)	0.610	25 (2.3)	7 (28.0)	18 (72.0)	25 (12–39)
Rickettsia sp.	6 (1.7)	18 (2.5)	0.404	24 (2.3)	6 (25.0)	18 (75.0)	31.5 (15–51)
Stretococcus pneumoniae	10 (2.8)	12 (1.7)	0.234	22 (2.1)	10 (45.5)	12 (54.5)	17 (0.5–28)
Mycobacterium tuberculosis	0	20 (2.8)	**0.001**	20 (1.9)	0	20 (100)	35 (20–53)
Other bacteria	16 (1.5)	32 (4.5)	**0.012**	48 (4.5)	16 (33.3)	32 (66.7)	23.5 (2.7–45)
Japanese encephalitis virus	50 (14.0)	44 (6.2)	**<0.001**	94 (8.8)	50 (53.2)	44 (46.8)	13 (8–20)
Dengue virus	8 (2.2)	19 (2.7)	0.624	27 (2.5)	8 (29.6)	19 (70.4)	20 (6–30)
Herpes simplex 1 and 2	3 (0.8)	12 (1.7)	0.237	15 (1.4)	3 (20.0)	12 (80.0)	32 (20–54)
Human cytomegalovirus	5 (1.4)	7 (1.0)	0.560	12 (1.1)	5 (41.7)	7 (58.3)	24 (0.3–37)
Enterovirus	8 (2.2)	2 (0.3)	**0.002**	10 (0.9)	8 (80.0)	2 (20.0)	4.5 (1–11)
Varicella zoster virus	0	6 (0.8)	0.090	6 (0.6)	0	6 (100)	35 (23–38)
Mumps virus	2 (0.6)	3 (0.4)	0.651	5 (0.5)	2 (40.0)	3 (60.0)	29 (14–53)
Plasmodium falciparum	1 (0.3)	3 (0.4)	0.799	4 (0.4)	1 (25.0)	3 (75.0)	17 (10.5–31.5)
Cryptococcus spp.	0	70 (9.9)	**<0.001**	70 (6.6)	0	70 (100)	33 (27–41)

*Children were patients <15 years of age, and adults were patients ≥15 years of age.

Appendix Table 9. Characteristics of patients with confirmed bacterial etiology in comparison with patients with no confirmed bacterial etiology, using univariate analysis*

Characteristic	Patients with bacterial etiology, n = 175	Patients with no bacterial etiology, n = 875	p value, χ^2	p value, Fisher
Demographic				
Male, n = 1,050	117 (66.9)	540 (61.7)	0.199	
Age, n = 1,050, y, median (IQR)	23.0 (9–45)	24 (8–38)	0.291	
Age group, n = 1,050				0.220
<1 mo	2 (1.1)	21 (2.4)		
1 mo–< 1 y	21 (12.0)	86 (9.8)		
1–<5 y	6 (3.4)	67 (7.7)		
5–<15 y	25 (14.3)	124 (14.2)		
≥15 y	121 (69.1)	577 (65.9)		
Distance from hospital, n = 1,046, km, median (IQR)	27 (9–56)	25 (7–92)	0.974	
Population density per km^2,† n = 1,036, median (IQR)	334 (92–1285)	422 (91–2011)	0.463	
Occupation,‡ n = 594			0.064	
Farmer	27 (27.3)	78 (15.8)		
Work indoors	10 (10.1)	67 (13.5)		
Work outdoors	23 (23.2)	125 (25.3)		
Student	14 (14.1)	61 (12.3)		
Other	25 (25.3)	164 (33.1)		
History				
HIV seropositive, n = 692	6 (6.2)	107 (18.0)	**0.004**	
Diabetic, n = 840	10 (7.5)	14 (2.0)	**<0.001**	
Tuberculosis, n = 723	2 (1.9)	31 (5.0)	0.143	
Antibiotic before LP, n = 940	100 (62.5)	478 (61.3)	0.773	
Steroid use before LP, n = 845	7 (5.3)	50 (7.0)	0.472	
Alcohol excess,§ n = 584	44 (43.1)	202 (41.9)	0.819	
Pet (dog or cat) at home, n = 576	90 (88.2)	424 (89.5)	0.719	
Poultry at home, n = 533	81 (88.0)	394 (89.3)	0.716	
Pigs at home, n = 409	54 (81.8)	285 (83.1)	0.802	
Signs and symptoms				
Days of fever at admission, n = 1,043, median (IQR)	5 (3–8)	4 (1–7)	**0.004**	
Fever, n = 1,044	171 (97.7)	776 (89.3)	**<0.001**	
Headache,¶ n = 883	135 (91.2)	642 (87.4)	0.186	
Neck stiffness, n = 1,049	128 (73.1)	546 (62.5)	**0.007**	
Confusion, n = 1,045	103 (59.5)	498 (57.1)	0.555	

Characteristic	Patients with bacterial etiology, n = 175	Patients with no bacterial etiology, n = 875	p value, χ^2	p value, Fisher
Drowsiness, n = 1,044	110 (63.6)	492 (56.5)	0.084	
Convulsions, n = 1,048	44 (25.3)	269 (30.8)	0.148	
GCS score, n = 997, median (IQR)	14 (11–15)	14 (11–15)	0.800	
GCS score <15,# n = 1,032	94 (54.0)	450 (52.5)	0.704	
Arthralgia,¶ n = 883	27 (18.3)	112 (15.2)	0.360	
Myalgia,¶ n = 883	75 (50.7)	340 (46.3)	0.326	
Rash, n = 1,043	19 (10.9)	126 (14.5)	0.213	
Vomiting or diarrhea, n = 1,049	101 (58.1)	466 (53.3)	0.247	
Cough, n = 1,049	39 (22.4)	216 (24.7)	0.523	
Shortness of breath, n = 1,049	23 (13.2)	130 (14.9)	0.576	
Cough or shortness of breath, n = 1,049	50 (28.7)	281 (32.1)	0.381	
Respiration rate, n = 1,020, breaths/min, median (IQR)	23 (20–28)	22 (20–30)	0.089	
WHO clinical CNS infection,** n = 1,025	140 (80.9)	621 (72.9)	**0.028**	
WHO encephalitis,** n = 1,025	102 (59.0)	470 (55.2)	0.359	
WHO meningitis,** n = 1,025	138 (79.8)	594 (69.7)	**0.008**	
WHO meningoencephalitis,** n = 1,025	100 (57.8)	443 (52.0)	0.163	
Fever + no neck stiffness + GCS score <15 and/or seizures, n = 1,025	16 (9.3)	110 (12.9)	0.181	
Fever + neck stiffness + GCS score of 15 + no seizures, n = 1,025	38 (22.0)	151 (17.7)	0.190	
Fever + neck stiffness + GCS score <15 and/or seizures, n = 1,025	86 (49.7)	360 (42.3)	0.071	
Fever + neck stiffness, n = 1,025	124 (71.7)	511 (60.0)	**0.004**	
Fever + GCS score<15 and/or seizures, n = 1,025	102 (59.0)	470 (55.2)	0.359	
Peripheral blood analysis				
Total leukocyte count, n = 938, × 10^3 cells/mm^3, median (IQR)	11.9 (8.2–16.4)	10.6 (7.5–14.2)	**0.034**	
Elevated leukocyte count,†† n = 938	84 (53.5)	360 (46.1)	0.090	
Low leukocyte count,†† n = 938	7 (4.5)	38 (4.9)	0.828	
Hematocrit, n = 934, %, median (IQR)	37 (31.5–41)	38 (33–42)	**0.049**	
Anemia,†† n = 934	68 (43.9)	279 (35.8)	**0.058**	
Platelets, n = 640, × 10^3 cells/mm^3, median (IQR)	220 (180–270)	218 (189–296)	0.604	
Thrombocytopenia,†† n = 640	12 (10.6)	41 (7.8)	0.320	
CRP, n = 856, mg/L, median (IQR)	64.4 (15.2–154.7)	16 (3.1–57.1)	**<0.001**	
Elevated CRP,†† n = 856	114 (79.7)	430 (60.3)	**<0.001**	
Creatinine, n = 770, μmol/L, median (IQR)	79.6 (61.9–106.1)	79.6 (53.0–106.1)	0.143	
Total bilirubin, n = 843, μmol/L, median (IQR)	6.8 (4.8–12.0)	5.1 (3.4–8.6)	**<0.001**	
ALP, n = 730, IU/L, median (IQR)	92.5 (69.5–161)	96 (66–156)	0.840	
ALT, n = 819, IU/L, median (IQR)	18 (11–38)	16 (11–28)	0.101	
AST, n = 831, IU/L, median (IQR)	48.5 (27–100)	46 (30–76)	0.303	
Blood glucose, n = 977, mmol/L, median (IQR)	5.5 (6.8–8.5)	5.2 (6.2–7.5)	**<0.001**	
Hyperglycemia,‡‡ n = 977	53 (32.3)	182 (22.4)	**0.007**	
Severe hyperglycemia,‡‡ n = 991	22 (13.4)	50 (6.2)	**0.001**	
CSF				
Turbid, n = 984	38 (23.2)	103 (12.6)	**<0.001**	
Hemorrhagic, n = 984	19 (11.6)	106 (12.9)	0.638	
Xanthochromia, n = 984	11 (6.7)	32 (3.9)	0.109	
Opening pressure, n = 962, H_2O cm, median (IQR)	20 (15.5–31.0)	20 (14–30)	0.219	
Elevated opening pressure,†† n = 962	60 (37.3)	269 (33.6)	0.369	
Red cell count, n = 873, cells/mm^3, median (IQR)	0 (0–10)	0 (0–5)	0.713	
Elevated red cells,†† n = 873	43 (28.7)	190 (26.3)	0.547	
Total white cell count, n = 961, cells/mm^3, median (IQR)	115 (20–415)	30 (5–155)	**<0.001**	
Elevated white cell count,†† n = 961	129 (80.1)	590 (73.8)	0.089	
Lymphocytes, n = 877, %, median (IQR)	15.1 (0–40)	25 (0–67)	0.074	
Elevated lymphocyte count,†† n = 877	91 (62.3)	371 (50.8)	**0.008**	
Neutrophils, n = 877, %, median (IQR)	70 (14.1–91)	45 (0–79)	**<0.001**	
Elevated neutrophil count,†† n = 876	116 (80.0)	518 (70.9)	**0.025**	
CSF eosinophilia,§§ n = 986	2 (1.2)	44 (5.4)		**0.023**
Protein, n = 941, g/L, median (IQR)	0.8 (0.3–1.6)	0.5 (0.3–1.1)	**<0.001**	
Elevated protein,†† n = 941	108 (69.7)	483 (61.5)	**0.053**	
Glucose, n = 943, mmol/L, median (IQR)	3.4 (2.2–4.8)	3.6 (2.4–4.9)	0.600	
Decreased glucose,†† n = 943	51 (32.9)	226 (28.7)	0.291	
Decreased CSF:venus glucose ratio,†† n = 916	97 (64.2)	435 (56.9)	0.093	
Lactate, n = 954, mmol/L, median (IQR)	4 (2.4–7.4)	2.6 (1.8–4.2)	**<0.001**	
Elevated lactate,†† n = 970	132 (80.5)	505 (62.7)	**<0.001**	
Treatment post LP				
Antibiotic, n = 1,004	166 (96.5)	754 (90.6)	**0.011**	
Steroid, n = 938	35 (21.1)	187 (24.2)	0.388	
Outcome				
Days of hospitalization, n = 837, median (IQR)	11 (7–17)	9 (5–14)	**0.028**	
Mortality and discharge moribund, n = 881	43 (27.9)	186 (25.6)	0.548	
Delays between admission and LP, n = 1,007, d, median (IQR)	1 (0–2)	1 (0–3)	0.230	

Characteristic	Patients with bacterial etiology, n = 175	Patients with no bacterial etiology, n = 875	p value, χ^2	p value, Fisher

*Values are no. (%) unless indicated otherwise. Bold values are statistically significant (p<0.05). Univariate analyses were performed to compare patients with confirmed bacterial infection (175, including patients with bacterial co-infection) to other patients (875, excluding patients with co-infection involving bacteria and virus or *Cryptococcus*). History or physical examination were taken into account for rash, confusion, neck stiffness, fever (history of fever or >37.5°C during physical examination). ALP, alkaline phosphatase; ALT, alanine transaminase; AST, aspartate transaminase; CRP, C-reactive protein; CNS, central nervous system; CSF, cerebrospinal fluid; GCS, Glasgow coma scale; IQR, interquartile range; LP, lumbar puncture; TB, *Mycobacterium tuberculosis*; WHO, World Health Organization.
†Population density of the village of residence: Population densities per village were from population census 2005, recovered from Lao DECIDE info Web site (platform of Government of Lao PDR, www.decide.la).
‡Occupation: work indoors = teacher, government official, business, factory worker, accountant; work outdoors = driver, building worker, merchant, carpenter, soldier, mechanic; other: housewife, no job, monk, retired, singer, health worker.
§Data collected for children (<15 years old) were excluded for analysis.
¶Considered as not reliable, the data were excluded from analysis for children <3 years old.
#Including confused and disoriented.
**WHO clinical CNS infection = fever with either GCS score <15, neck stiffness (history or examination), or history of seizure, patients with missing data for 1 of those criteria were not counted. WHO encephalitis = fever with GCS score <15 or history of seizure or both. WHO meningitis = fever with GCS score <15 or neck stiffness or both. WHO meningoencephalitis = meeting both WHO encephalitis and WHO meningitis criteria.
††Elevated and low parameters = above or below normal ranges (Appendix Table 3), anemia: hematocrit below normal range. In elevated CSF white cell count, were not taken into account the cases that could not be counted because of high turbidity.
‡‡Hyperglycemia: blood glucose higher than 7.7 mmol/L, severe hyperglycemia: blood glucose higher than 11.1 mmol/L.
§§Eosinophilia: CSF eosinophils >10%.

Appendix Table 10. Estimation of the risk factors associated with bacterial infection, using multivariate logistic regression models*

	% Missing	Complete case analysis, n = 532†			MICE, n = 1,043‡		
Factor	values	aOR	p value	95% CI	aOR	p value	95% CI
Diabetes§	20	4.26†	0.005†	1.54–11.79†	**3.09‡**	**0.015‡**	**1.24–7.68‡**
Total bilirubin§	19.7	0.98	0.849	0.84–1.16	0.99	0.944	0.85–1.16
C-reactive protein§	18.5	1.06†¶	0.001†	1.03–1.10†¶	**1.08‡¶**	**<0.001‡**	**1.05–1.11‡¶**
CSF protein§	10.4	0.95	0.504	0.80–1.11	1.00	0.943	0.91–1.09
CSF lactate§	9.1	3.88†¶	<0.001†	2.29–6.57†¶	**3.51‡¶**	**<0.001‡**	**2.30–5.35‡¶**
CSF white cell count§	8.5	1.00	0.675	1.00–1.00	1.00	0.821	1.00–1.00
Turbid CSF§	6.3	0.54	0.190	0.22–1.36	0.90	0.699	0.52–1.56
Fever	0.6	3.72†	0.039†	1.07–12.95†	**3.87‡**	**0.011‡**	**1.36–11.06‡**
Neck stiffness	0.1	1.08	0.793	0.62–1.88	1.21	0.341	0.81–1.81

*The factors that showed p<0.01 in univariate analysis were submitted to multivariate analysis. Some factors were excluded (e.g., HIV seropositivity), since the choice for patient testing was biased. Clinical meningitis was correlated with neck stiffness, neutrophils was correlated with white cell count, and hyperglycemia was correlated with diabetes (a model was run replacing diabetes with hyperglycemia or blood glucose, which turned out to be not significant). aOR, adjusted odds ratio; CSF, cerebrospinal fluid; MICE, multiple imputation by chained equation.
†Complete case analysis was repeated with only significant factors (p<0.05) identified by stepwise approach (n = 607).
‡Final model with imputed values with only significant variables included (n = 1,043).
§Variables with imputed values. Other variables included in the imputation model: bacterial infection (outcome), sex, age, fever, and neck stiffness.
¶The aOR for a 10-U increase in C-reactive protein or CSF lactate.

Appendix Table 11. Characteristics of patients with confirmed viral etiology in comparison with patients with no confirmed viral etiology, using univariate analysis*

Characteristic	Patients with viral etiology, n = 172	Patients with no viral etiology, n = 867	p value, χ^2	p value, Fisher
Demographic				
Male, n = 1,039	111 (64.5)	539 (62.2)	0.558	
Age, n = 1,039, y, median (IQR)	16 (7–28)	25 (8–41)	**<0.001**	
Age group, n = 1,039			**<0.001**	
<1 mo old	2 (1.2)	21 (2.4)		
1 mo–< 1 y old	9 (5.2)	98 (11.3)		
1–< 5 y old	21 (12.2)	52 (6.0)		
5–<15 y old	45 (26.2)	104 (12.0)		
≥15 y old	95 (55.2)	592 (68.3)		
Distance from hospital, n = 1,035, km, median (IQR)	39 (8–133)	23 (7–76)	**0.021**	
Population density,† n = 1,025, per km^2, median (IQR)	433 (70–1,821)	403 (94–1,949)	0.378	
Occupation,‡ n = 583, adults only			**0.012**	
Farmer	14 (17.7)	91 (18.1)		
Work indoors	10 (12.7)	67 (13.3)		
Work outdoors	16 (20.3)	125 (24.8)		
Student	20 (25.3)	54 (10.7)		
Other	18 (24.1)	167 (33.1)		
History				
HIV seropositive, n = 681	8 (8.0)	94 (16.2)	**0.034**	
Diabetic, n = 834	1 (0.8)	23 (3.3)		0.155
History of TB, n = 717	3 (2.7)	26 (4.3)		0.603
Antibiotic use before LP, n = 935, (%)	109 (69.9)	469 (60.2)	**0.023**	
Steroid use before LP, n = 836	9 (6.9)	48 (6.8)	0.959	
Alcohol excess,§ n = 574	29 (36.7)	214 (43.2)	0.276	
Pet (dog or cat) at home, n = 585	81 (91.0)	428 (88.8)	0.537	
Poultry at home, n = 539	86 (89.6)	389 (89.2)	0.917	
Pigs at home, n = 404	70 (86.4)	264 (81.7)	0.319	
Signs and symptoms				
Days of fever at admission, n = 1,032, median (IQR)	5 (3–7)	4 (1–8)	0.285	
Fever, n = 1,033	162 (95.3)	775 (89.8)	**0.024**	
Headache,¶ n = 872	139 (90.9)	627 (87.2)	0.210	
Neck stiffness, n = 1,034	130 (75.6)	538 (62.1)	**0.001**	
Confusion, n = 1,034	114 (66.3)	483 (56.0)	**0.013**	
Drowsiness, n = 1,033	111 (64.9)	488 (56.6)	**0.045**	
Convulsions, n = 1,037	65 (37.8)	247 (28.6)	**0.016**	
GCS score, n = 986, median (IQR)	13 (10–15)	14 (11–15)	0.103	
GCS score <15,# n = 1,021	101 (59.4)	441 (51.8)	0.070	
Arthralgia,¶ n = 872	20 (13.1)	119 (16.6)	0.286	
Myalgia,¶ n = 872	72 (47.1)	341 (47.4)	0.934	
Rash, n = 1,032	20 (11.7)	120 (13.9)	0.434	
Vomiting or diarrhea, n = 1,038	101 (58.7)	460 (53.1)	0.178	
Cough or shortness of breath, n = 1,038	47 (27.3)	280 (32.3)	0.197	
Cough, n = 1,038	35 (20.4)	216 (24.9)	0.199	
Shortness of breath, n = 1,038	20 (11.6)	132 (15.2)	0.221	
Respiratory rate, n = 1,009, breaths/min, median (IQR)	24 (20–32)	22 (20–28)	**0.025**	
WHO clinical CNS infection,** n = 1,014	143 (85.1)	611 (72.2)	**<0.001**	
WHO encephalitis,** n = 1,014	107 (63.7)	462 (54.6)	**0.030**	
WHO meningitis,** n = 1,014	140 (83.3)	586 (69.3)	**<0.001**	
WHO meningoencephalitis,** n = 1,014	104 (61.9)	437 (51.7)	**0.015**	
Fever + no neck stiffness + GCS score <15 and/or seizures, n = 1,014	17 (10.1)	107 (12.7)	0.361	
Fever + neck stiffness + GCS score of 15 + no seizures, n = 1,014	36 (21.4)	149 (17.6)	0.242	
Fever + neck stiffness + GCS score <15 and/or seizures, n = 1,014	90 (53.6)	355 (42.0)	**0.006**	
Fever + neck stiffness, n = 1,014	126 (75.0)	504 (59.6)	**<0.001**	
Fever + GCS score <15 and/or seizures, n = 1,014	107 (63.7)	462 (54.6)	**0.030**	
Peripheral blood analysis				
Total leukocyte count, n = 930, 10^3cells/mm^3, median (IQR)	11.6 (8.6–14.5)	10.7 (7.4–14.6)	0.296	
Elevated white cell count,†† n = 930	84 (53.9)	359 (46.4)	0.089	
Low white cell count,†† n = 930	6 (3.9)	38 (4.9)	0.568	
Hematocrit, n = 926, %, median (IQR)	39 (35–43.5)	38 (32.7–42)	**0.003**	
Anemia, †† n = 926	44 (28.2)	296 (38.4)	**0.016**	
Platelet, n = 635, 10^3count/mm^3, median (IQR)	220 (200–299)	217 (180–290)	0.107	
Thrombocytopenia, †† n = 635	4 (3.5)	47 (9.1)	**0.045**	
CRP, n = 846, mg/L, median (IQR)	19.2 (4.7–57.2)	21.6 (3.5–79)	0.543	
Elevated CRP, †† n = 846	98 (64.9)	439 (63.2)	0.688	
Creatinine, n = 759, μmol/L, median (IQR)	70.7 (53.0–88.4)	79.6 (61.9–106.1)	**0.031**	
Total bilirubin, n = 834, μmol/L, median (IQR)	5.1 (3.4–8.7)	5.3 (3.4–9.6)	0.084	
ALP, n = 721, IU/L, median (IQR)	105 (74–144.5)	92 (66–160)	0.730	
ALT, n = 810, IU/L, median (IQR)	14 (10–23)	17 (11–31)	**0.028**	

Characteristic	Patients with viral etiology, n = 172	Patients with no viral etiology, n = 867	p value, χ^2	p value, Fisher
AST, n = 822, IU/L, median (IQR)	44.5 (28–68)	46 (29–82.5)	0.196	
Hyperglycemia,‡‡ n = 967	40 (24.0)	193 (24.1)	0.962	
Severe hyperglycemia,‡‡ n = 967	12 (7.2)	60 (7.5)	0.888	
CSF				
Turbid, n = 973	21 (12.4)	117 (14.6)	0.471	
Hemorrhagic, n = 973	22 (13.0)	103 (12.8)	0.942	
Xanthochromia, n = 973	5 (3.0)	37 (4.6)	0.339	
Opening pressure, n = 953, H_2O cm, median (IQR)	20 (15–26.5)	20 (14–31)	0.534	
Elevated opening pressure,†† n = 953	42 (24.9)	280 (35.7)	**0.007**	
Red cell count, n = 864, cells/mm^3, median (IQR)	0 (0–5)	0 (0–5)	0.571	
Elevated red cell count,†† n = 864	39 (24.5)	194 (27.5)	0.443	
Total white cell count, n = 951, cells/mm^3, median (IQR)	82.5 (25–275)	30 (5–190)	**<0.001**	
Elevated white cell count,†† n = 951	141 (84.9)	574 (73.1)	**0.001**	
Lymphocytes, n = 867, %, median (IQR)	33.3 (2–71)	22 (0–58.5)	**0.006**	
Elevated lymphocyte count,†† n = 867	106 (68.4)	354 (49.7)	**<0.001**	
Neutrophils, n = 867, %, median (IQR)	48.4 (19–83)	50 (0–83)	0.264	
Elevated neutrophil count,†† n = 866	130 (83.9)	503 (70.8)	**0.001**	
CSF eosinophilia,§§ n = 976	9 (5.3)	37 (4.6)	0.680	
Protein, n = 931, g/L, median (IQR)	0.65 (0.34–1.2)	0.55 (0.3–1.18)	0.400	
Elevated protein,†† n = 931	112 (66.3)	475 (62.3)	0.337	
Glucose, n = 933, mmol/L, median (IQR)	3.56 (2.5–4.56)	3.56 (2.33–5)	0.527	
Decreased glucose,†† n = 933	45 (26.6)	228 (29.8)	0.406	
Decreased CSF:venus glucose ratio,†† n = 906	97 (58.8)	429 (57.9)	0.833	
Lactate, n = 945, mmol/L, median (IQR)	2.3 (1.8–3.4)	2.8 (1.9–4.9)	**0.001**	
Elevated lactate,†† n = 985	93 (56.0)	538 (67.7)	**0.004**	
Treatment post LP				
Treatment antibiotic, n = 993	163 (97.0)	746 (90.4)	**0.005**	
Treatment steroid, n = 930	38 (24.2)	183 (23.7)	0.887	
Outcome				
Days of hospitalization, n = 833, median (IQR)	10 (6–14)	9 (5–14)	0.425	
Mortality and discharged moribund, n = 878	23 (15.7)	207 (28.3)	**0.001**	
Delay between admission and LP, n = 996, d, median (IQR)	0 (0–2)	1 (0–3)	**<0.001**	

*Values are no. (%) unless indicated otherwise. Bold values are statistically significant (p<0.05). Univariate analyses were performed to compare patients with confirmed viral infection (172, including patients with viral co-infection) to other patients (867, excluding patients with co-infection involving virus and bacteria or *Cryptococcus*). History or physical examination were taken into account for rash, confusion, neck stiffness, fever (history of fever or >37.5°C during physical examination). ALP, alkaline phosphatase; ALT, alanine transaminase; AST, aspartate transaminase; CNS, central nervous system; CRP, C-reactive protein; CSF, cerebrospinal fluid; GCS, Glasgow coma scale; IQR, interquartile range; LP, lumbar puncture; TB, *Mycobacterium tuberculosis*; WHO, World Health Organization.

†Population density of the village of residence: Population densities per village were from population census 2005, recovered from Lao DECIDE info website (platform of Government of Lao PDR, www.decide.la).

‡Occupation: work indoors = teacher, government official, business, factory worker, accountant; work outdoors = driver, building worker, merchant, carpenter, soldier, mechanic; other: housewife, no job, monk, retired, singer, health worker.

§Data collected for children (<15 years old) were excluded for analysis.

¶Considered as not reliable, the data were excluded from analysis for children <3 y old.

#Including confused and disoriented.

**WHO clinical CNS infection: fever with either GCS score<15, neck stiffness (history or examination), or history of seizure, patients with missing data for 1 of those criteria were not counted. WHO encephalitis = fever with GCS score <15 or history of seizure or both. WHO meningitis = fever with GCS score <15 or neck stiffness or both. WHO meningoencephalitis = meeting both WHO encephalitis and WHO meningitis criteria.

††Elevated and low parameters = above or below normal ranges (Appendix Table 3), anemia: hematocrit below normal range. In elevated CSF white cell count, were not taken into account the cases that could not be counted because of high turbidity.

‡‡Hyperglycemia: blood glucose higher than 7.7 mmol/L, severe hyperglycemia: blood glucose higher than 11.1 mmol/L.

§§Eosinophilia: CSF eosinophils >10%.

Appendix Table 12. Estimation of the risk factors associated with viral infection, using multivariate logistic regression models*

Factor	% Missing values	Complete case analysis, n = 777†			MICE, n = 1,035‡		
		aOR	p value	95% CI	aOR	p value	95% CI
Hematocrit§	10.9	1.36†¶	0.023†	1.04–1.78†¶	**1.43‡¶**	**0.007‡**	**1.10–1.85‡¶**
CSF lactate§	9.0	0.29†¶	0.001†	0.14–0.61†¶	**0.25‡¶**	**<0.001‡**	**0.12–0.51‡¶**
CSF white cell count§	8.5	1.00	0.203	1.00–1.00	1.00	0.208	1.00–1.00
Elevated CSF opening pressure§	8.3	0.72	0.145	0.46–1.12	0.68	0.058	0.45–1.01
Days between admission and LP	0.3	0.87†	0.004†	0.79–0.96†	**0.89‡**	**0.005‡**	**0.82–0.97‡**
Neck stiffness	0.1	1.92†	0.003†	1.25–2.93†	**1.93‡**	**0.001‡**	**1.31–2.84‡**
Age	0	0.84†¶	0.002†	0.76–0.94†¶	**0.82‡¶**	**<0.001‡**	**0.74–0.91‡¶**

*The factors that showed p<0.01 in univariate analysis were submitted to multivariate analysis. Some factors were excluded: clinical meningitis, meningoencephalitis and clinical CNS infection that are correlated with neck stiffness, neutrophils and lymphocytes that are correlated with white cell count. aOR, adjusted odds ratio; CSF, cerebrospinal fluid; LP, lumbar puncture; MICE, multiple imputation by chained equation.
†Complete case analysis was repeated with only significant factors (p<0.05) identified by stepwise approach (n = 839).
‡Final model with imputed values with only significant variables included (n = 1,035).
§Variables with imputed values. Other variables included in the imputation model: viral infection (outcome), sex, age, neck stiffness, days between admission and LP.
¶aOR for a 10-U increase in hematocrit, CSF lactate or age.

Appendix Table 13. Distribution of patients with confirmed etiology according to clinical presentations compatible with CNS infection*

Etiology	All, n = 1,065	Fever + no neck stiffness + GCS score <15 and/or seizures, n = 127	Fever + neck stiffness + GCS score of 15 + no seizures, n = 191	Fever + neck stiffness + GCS score <15 and/or seizures, n = 453	No CNS infection,† n = 269	GCS score <15, n = 551	Neck stiffness, n = 683	Seizures, n = 319	Fever, n = 962
Confirmed etiology	450 (42.3)	37 (29.1)	103 (53.9)	201 (44.4)	102 (37.9)	225 (40.8)	316 (46.3)	119 (37.3)	425 (44.2)
Co-infection	37 (3.5)	4 (3.1)	11 (5.8)	11 (2.4)	11 (4.1)	13 (2.4)	23 (3.4)	9 (2.8)	36 (3.7)
Bacterial (including bacterial co-infections)	175 (16.4)	16 (12.6)	38 (19.9)	86 (20.0)	33 (12.3)	94 (17.1)	128 (18.7)	44 (13.8)	171 (17.8)
Mycobacterium tuberculosis	20 (1.9)	1 (0.8)	2 (1.0)	14 (3.1)	3 (1.1)	15 (2.7)	17 (2.5)	2 (0.6)	19 (2.0)
Streptococcus pneumoniae	22 (2.1)	4 (3.1)	3 (1.6)	14 (3.1)	0	17 (3.1)	18 (2.6)	10 (3.1)	22 (2.3)
Leptospira spp.	25 (2.3)	2 (1.6)	6 (3.1)	11 (2.4)	6 (2.2)	12 (2.2)	17 (2.5)	5 (1.6)	25 (2.6)
Rickettsia spp.	24 (2.3)	1 (0.8)	3 (1.0)	14 (3.1)	6 (2.2)	14 (2.5)	17 (2.5)	4 (1.3)	24 (2.5)
Orientia tsutsugamushi	31 (2.9)	3 (2.4)	13 (6.8)	10 (2.2)	5 (1.9)	10 (1.8)	23 (3.4)	7 (2.2)	31 (3.2)
Other bacteria	48 (4.5)	4 (3.1)	9 (4.7)	21 (4.6)	13 (4.8)	23 (4.2)	32 (4.7)	15 (4.7)	45 (4.7)
Cryptococcus spp.	70 (6.6)	1 (0.8)	20 (10.5)	15 (3.3)	33 (12.3)	19 (3.4)	38 (5.6)	2 (0.6)	60 (6.2)
Viral (including viral co-infections)	172 (16.2)	17 (13.4)	36 (18.8)	90 (19.9)	25 (9.3)	101 (18.3)	130 (19.0)	65 (20.4)	162 (16.8)
JEV	94 (8.8)	7 (4.7)	15 (7.9)	67 (14.8)	5 (1.9)	68 (12.3)	82 (12.0)	40 (12.5)	92 (9.6)
Dengue virus	27 (2.5)	5 (3.9)	5 (2.6)	11 (2.4)	5 (1.9)	17 (3.1)	18 (2.6)	9 (1.6)	24 (2.5)
HCMV	12 (1.1)	1 (0.8)	2 (1.0)	4 (0.9)	4 (1.5)	6 (1.1)	6 (0.9)	5 (1.6)	10 (1.0)
HSV1/2	15 (1.4)	3 (2.4)	3 (1.6)	4 (0.9)	4 (1.5)	7 (1.3)	8 (1.2)	7 (2.2)	13 (1.4)
Enterovirus	10 (0.9)	0	5 (2.6)	3 (0.7)	2 (0.7)	2 (0.4)	8 (1.2)	2 (0.6)	10 (1.0)
VZV	6 (0.6)	0	2 (1.0)	0	3 (1.1)	0	2 (0.3)	0	6 (0.6)
Mumps	5 (0.5)	1 (0.8)	2 (1.0)	0	2 (0.7)	0	3 (0.4)	1 (0.3)	4 (0.4)
Malaria	4	0	2 (1.0)	2 (0.4)	0	2 (0.4)	4 (0.6)	1 (25)	4 (100)

*In the table are reported number of patients (percentage). Syndromic classification was done only for patients with data available for all criteria: fever (history of fever or >37.5°C during physical examination), neck stiffness (history or examination), GCS score and history of seizure = 1,040 patients. Among the 25 patients with missing data, 1 was confirmed for *S. pneumoniae*, 1 for *Streptococcus agalactiae*, 1 for *Cryptococcus* spp.1 for *Dengue virus*, 1 for HCMV, 1 for HSV1/2, 1 for VZV. Fever = history of fever or documented fever (>37.5°C), neck stiffness = history or at examination, Seizures = history of seizures, GCS score <15 = GCS score total <15 and when GCS score total is missing = confused or disoriented. CNS, central nervous system; GCS, Glasgow coma scale; HCMV, human cytomegalovirus; HSV, herpes simplex virus; JEV, *Japanese encephalitis virus*; VZV, varicella zoster virus.
†No CNS infection = patients who don't meet criteria for World Health Organization clinical CNS infection (fever with either GCS score<15, neck stiffness, or history of seizures).

Appendix Table 14. Characteristics of patients who died or were discharged moribund in comparison with patients who were discharged alive and well*

Characteristic	Patients who died/discharged moribund, n = 235	Patients discharged alive and well, n = 658	p value, χ^2	p value, Fisher
Demographic				
Patient number, n = 893	235 (26.3)	658 (73.7)		
Male, n = 893	147 (62.6)	407 (61.9)	0.850	
Age, n = 893, y, median (IQR)	28 (9–45)	21 (7–36)	**0.007**	
Age group, n = 893			0.364	
<1 mo old	4 (1.7)	14 (2.1)		
1 mo–<1 y old	24 (10.2)	71 (10.8)		
1–<5 y old	16 (6.8)	53 (8.1)		
5–<15 y old	26 (11.1)	103 (15.7)		
≥15 y	165 (70.2)	417 (63.4)		
Distance from hospital, n = 889, km, median (IQR)	27.3 (7.2–99.7)	23.8 (6.7–80.2)	0.307	
Population density,† n = 879, per km^2, median (IQR)	444.1 (92.9–1,652.7)	403.1 (91.5–1,949.4)	**0.004**	
Occupation, for adults,‡ n = 504			**0.002**	
Farmer	26 (18.31)	68 (18.8)		
Work indoors	18 (12.7)	47 (13.0)		
Work outdoors	38 (26.8)	86 (23.8)		
Student	6 (4.2)	58 (16.0)		
Other	54 (38.0)	103 (28.5)		
History				
HIV seropositive, n = 583	18 (12.8)	53 (12.0)	0.806	
Diabetic, n = 727	10 (5.2)	11 (2.1)	**0.026**	
History of tuberculosis, n = 635	9 (5.6)	13 (2.7)	0.088	
Antibiotic use before LP, n = 811, %	131 (60.9)	361 (60.6)	0.926	
Steroid use before LP, n = 725	21 (11.2)	33 (6.1)	**0.022**	
Alcohol excess,§ n = 482	65 (46.8)	152 (44.3)	0.625	
Pet (dog or cat) at home, n = 493	120 (90.9)	319 (88.4)	0.423	
Poultry at home, n = 462	103 (87.3)	305 (88.7)	0.688	
Pigs at home, n = 348	75 (86.2)	212 (81.2)	0.290	
Signs and symptoms				
Days of fever at admission, n = 891, median (IQR)	4 (2–8)	4 (2–7)	0.971	
Fever, n = 891	220 (93.6)	591 (90.1)	0.105	
Headache,¶ n = 746	164 (82.8)	497 (90.7)	**0.003**	
Neck stiffness, n = 892	163 (69.4)	432 (65.8)	0.314	
Confusion, n = 890	179 (76.2)	356 (54.2)	**<0.001**	
Drowsiness, n = 889	141 (60.5)	384 (58.5)	0.598	
Convulsions, n = 891	79 (33.6)	199 (30.3)	0.352	
GCS score, n = 847, median (IQR)	11 (8–15)	15 (12–15)	**<0.001**	
GCS score <15,# n = 882	170 (73.6)	319 (49.0)	**<0.001**	
Arthralgia,¶ n = 746	25 (12.6)	87 (15.9)	0.273	
Myalgia,¶ n = 746	84 (42.4)	272 (49.6)	0.082	
Rash, n = 889	40 (17.1)	80 (12.2)	0.062	
Vomiting or diarrhea, n = 892	115 (48.9)	376 (57.2)	**0.028**	
Cough or shortness of breath, n = 892	83 (35.3)	197 (30.0)	0.130	
Cough, n = 892	63 (26.8)	151 (23.0)	0.239	
Shortness of breath, n = 892	54 (23.0)	80 (12.2)	**<0.001**	
Respiration rate, n = 872, breaths/min, median (IQR)	22.5 (20–30)	22 (20–30)	0.204	
WHO clinical CNS infection,** n = 878	200 (86.6)	470 (72.6)	**<0.001**	
WHO encephalitis,** n = 878	170 (73.6)	342 (52.9)	**<0.001**	
WHO meningitis,** n = 878	196 (84.9)	452 (69.9)	**<0.001**	
WHO meningoencephalitis,** n = 878	166 (71.9)	324 (50.1)	**<0.001**	
Fever + no neck stiffness + GCS score<15 and/or seizures, n = 878	42 (18.2)	64 (9.9)	**0.001**	
Fever + neck stiffness + GCS score of 15 + no seizures, n = 878	30 (13.0)	128 (19.8)	**0.021**	
Fever + neck stiffness + GCS score<15 and/or seizures, n = 878	128 (55.4)	278 (43.0)	**0.001**	
Fever + neck stiffness, n = 878	158 (68.4)	406 (62.8)	0.124	
Fever + GCS score <15 and/or seizures, n = 878	170 (73.6)	342 (52.9)	**<0.001**	
Peripheral blood analysis				
Total leukocyte count, n = 829, 10^3cells/mm^3, median (IQR)	10.8 (8.0–15.6)	10.8 (7.8–14.3)	0.848	
Elevated leukocyte count,†† n = 829	106 (49.1)	289 (47.2)	0.625	
Low leukocyte count,†† n = 829	8 (3.7)	25 (4.1)	0.809	
Hematocrit, n = 826, %, median (IQR)	38 (32–41)	38 (33.6–42)	0.133	
Anemia, n = 826	92 (43.0)	210 (34.3)	**0.023**	
Platelets, n = 595, 10^3count/mm^3, median (IQR)	210 (180–280)	220 (190–290)	0.339	
Thrombocytopenia,†† n = 595	14 (9.5)	35 (7.8)	0.532	
CRP, n = 712, mg/L, median (IQR)	33.5 (9.3–106.3)	15.9 (2.9–58.8)	**<0.001**	
Elevated CRP,†† n = 712	143 (77.3)	307 (58.3)	**<0.001**	

Characteristic	Patients who died/discharged moribund, n = 235	Patients discharged alive and well, n = 658	p value, χ^2	p value, Fisher
Creatinine, n = 640, µmol/L, median (IQR)	79.6 (61.9–132.6)	79.6 (53.0–97.2)	**0.018**	
Total bilirubin, n = 701, µmol/L, median (IQR)	5.8 (3.6–10.3)	5.1 (3.4–10.3)	0.538	
ALP, n = 600, IU/L, median (IQR)	93 (64.5–140)	96 (68–161.5)	0.113	
ALT, n = 681, IU/L, median (IQR)	17 (11–31)	16 (10–28)	0.065	
AST, n = 690, IU/L, median (IQR)	50 (33–99)	42 (27–73)	**0.002**	
Hyperglycemia,‡‡ n = 836	72 (32.7)	140 (22.7)	**0.003**	
Severe hyperglycemia,‡‡ n = 836	25 (11.4)	41 (6.7)	**0.026**	
CSF				
Turbid, n = 840	34 (15.0)	88 (14.3)	0.795	
Hemorrhagic, n = 840	37 (16.4)	66 (10.8)	**0.028**	
Xanthochromia, n = 840	12 (5.3)	25 (4.1)	0.438	
Opening pressure, n = 823, H_2O cm, median (IQR)	20 (14–33.3)	20 (14–29)	0.219	
Elevated opening pressure,†† n = 823	81 (37.5)	194 (32.0)	0.138	
Red cell count, n = 740, cells/mm^3, median (IQR)	0 (0–5)	0 (0–5)	0.886	
Elevated red cells,†† n = 740	53 (27.0)	148 (27.2)	0.964	
Total white cell count, n = 822, cells/mm^3, median (IQR)	30 (5–185)	45 (10–240)	0.080	
Elevated white cell count,†† n = 822	160 (74.4)	466 (76.8)	0.487	
Lymphocytes, n = 746, %, median (IQR)	25 (0–67)	26 (0–63)	0.656	
Elevated lymphocyte count,†† n = 746	99 (50.8)	301 (54.6)	0.353	
Neutrophils, n = 746, %, median (IQR)	50 (0–82.6)	50 (0–83)	0.526	
Elevated neutrophil count,†† n = 746	140 (71.8)	408 (74.1)	0.540	
CSF eosinophilia,§§ n = 845	7 (3.1)	33 (5.3)	0.176	
Protein, n = 805, g/L, median (IQR)	0.74 (0.33–1.63)	0.57 (0.32–1.08)	**0.013**	
Elevated protein,†† n = 805	143 (67.5)	384 (64.8)	0.478	
Glucose, n = 807, mmol/L, median (IQR)	3.81 (2.25–5.61)	3.61 (2.5–4.78)	0.391	
Decreased glucose,†† n = 807	70 (33.0)	156 (26.2)	0.058	
Decreased CSF:venous glucose ratio,†† n = 783	122 (60.4)	326 (56.1)	0.289	
Lactate, n = 814, mmol/L, median (IQR)	3.5 (2.3–6.2)	2.6 (1.8–4.3)	**<0.001**	
Elevated lactate,†† n = 827	175 (78.5)	372 (61.6)	**<0.001**	
Treatment post LP				
Treatment antibiotic, n = 874	214 (94.3)	586 (90.6)	0.085	
Treatment steroid, n = 845	63 (28.3)	135 (21.7)	**0.048**	
Delay in LP, n = 862				
Days between admission and LP, median (IQR)	1 (0–2)	1 (0–2)	0.640	
>2 d between admission and LP	56 (24.2)	147 (23.3)	0.772	

*Values are no. (%) unless indicated otherwise. Univariate analysis was performed to compare patients who died (235, including discharge moribund) to patients who were discharged alive (658). Bolded values are statistically significant. History or physical examination were taken into account for: rash, confusion, neck stiffness, fever (history of fever or >37.5°C during physical examination). ALP, alkaline phosphatase; ALT, alanine transaminase; AST, aspartate transaminase; CNS, central nervous system; CRP, C-reactive protein; CSF, cerebrospinal fluid; GCS, Glasgow coma scale; IQR, interquartile range; LP, lumbar puncture; TB, *Mycobacterium tuberculosis*; WHO, World Health Organization.

†Population density of the village of residence: Population densities per village were from population census 2005, recovered from Lao DECIDE info website (platform of Government of Lao PDR, www.decide.la).

‡Occupation: work indoors = teacher, government official, business, factory worker, accountant; work outdoors = driver, building worker, merchant, carpenter, soldier, mechanic; other: housewife, no job, monk, retired, singer, health worker.

§Data collected for children (<15 years old) were excluded for analysis.

¶Considered as not reliable, the data were excluded from analysis for children <3 y old.

#Including confused and disoriented.

**WHO clinical CNS infection: fever with either GCS score <15, neck stiffness (history or examination), or history of seizure, patients with missing data for 1 of those criteria were not counted. WHO encephalitis = fever with GCS score<15 or history of seizure or both. WHO meningitis = fever with GCS score <15 or neck stiffness or both. WHO meningoencephalitis = meeting both WHO encephalitis and WHO meningitis criteria.

††Elevated and low parameters = above or below normal ranges (Appendix Table 3), anemia: hematocrit below normal range. In elevated CSF white cell count, were not taken into account the cases that could not be counted because of high turbidity.

‡‡Hyperglycemia: blood glucose higher than 7.7 mmol/L, severe hyperglycemia: blood glucose higher than 11.1 mmol/L.

§§Eosinophilia: CSF eosinophils >10%.

Appendix Table 15. Estimation of the risk factors associated with death*

Factors	% Missing values	Complete case analysis, n = 515†			MICE, n = 950‡		
		aOR	p value	95% CI	aOR	p value	95% CI
Aspartate aminotransferase§	20.9	1.0	0.098	1.0–1.0	1.0	0.058	1.0–1.0
C-reactive protein§	18.5	1.0†	0.011†	1.0–1.0†	1.0	0.052	1.0–1.0
Hyperglycemia¶	6.9	0.9	0.824	0.5–1.6			
Adult occupation§#	9.8						
Work inside		0.7†	0.398†	0.3–1.7†	1.1	0.900	0.5–2.3
Work outside		0.8†	0.526†	0.4–1.6†	1.1	0.749	0.6–2.2
Student		0.2†	0.010†	0.1–0.7†	0.3	0.049	0.1–1.0
Other		1.2†	0.588†	0.6–2.4†	1.3	0.341	0.7–2.5
Child		0.5†	0.018†	0.2–0.9†	0.7	0.365	0.3–1.6
CSF lactate§	9.0	1.1†	0.009†	1.0–1.1†	**1.1‡**	**0.001‡**	**1.0–1.1‡**
GCS score§	5.2	0.8†	<0.001†	0.8–0.9†	**0.8‡**	**<0.001‡**	**0.8–0.9‡**
Viral infection	2.4	0.5	0.035	0.2–1.0	**0.4‡**	**0.001‡**	**0.3–0.7‡**
Village population density	1.3	1.0	0.698	1.0–1.0	1.0	0.850	1.0–1.0
Bacterial infection	1.4	0.6	0.191	0.3–1.2	0.6	0.036	0.3–1.0
Confusion	0.5	2.1	0.026	1.1–4.2	1.0	0.888	0.6–1.7
Headache**	0.1	0.6	0.162	0.3–1.2	0.6	0.123	0.3–1.1
Shortness of breath	0.1	1.3	0.375	0.7–2.6	1.4	0.145	0.9–2.4
Age	0	1.0	0.308	1.0–1.0	1.0	0.995	1.0–1.0

*The factors that showed p<0.01 in univariate analysis were submitted to multivariate analysis. Some factors were excluded: clinical central nervous system infection, meningitis, encephalitis, menignoencephalitis that are correlated with GCS score. aOR, adjusted odds ratio; CSF, cerebrospinal fluid; GCS, Glasgow coma scale; MICE, multiple imputation by chained equation.
†Complete case analysis was repeated with only significant factors (p<0.05) identified by stepwise approach (n = 572).
‡Final model with imputed values with only significant variables included (n = 984).
§Variables with imputed values, plus mortality (including moribund, as outcome, 16.2% of missing values). Other variables included in the imputation model: sex, age, headache, confusion, GCS score, shortness of breath, village population density.
¶Hyperglycemia: blood glucose higher than 7.7 mmol/L.
#With farmer as reference group.
**Data provided only for adults and children ≥3 y old.

Appendix Table 16. In patients with confirmed etiology, the proportion of patients with etiology treatable by ceftriaxone or doxycycline among patients presenting with criteria consistent with bacterial meningitis*

Patients with confirmed etiology and:	Etiology treatable by ceftriaxone (not including *Leptospira*), no. (%)	Etiology treatable by ceftriaxone (including *Leptospira*), no. (%)	Etiology treatable by doxycycline (not including *Leptospira*), no. (%)	Etiology treatable by doxycycline (including *Leptospira*), no. (%)	Other confirmed etiologies, no. (%)
Neck stiffness,† n = 316	41 (13.0)	60 (19.0)	46 (14.6)	63 (19.9)	213 (67.4)
GCS score <15, n = 225	34 (15.1)	47 (20.9)	27 (12.0)	40 (17.8)	152 (67.6)
Elevated CRP, n = 265	44 (16.6)	60 (22.6)	36 (13.6)	51 (19.2)	171 (64.5)
CSF turbid, n = 80	27 (33.8)	31 (38.8)	6 (7.5)	9 (11.5)	45 (54.3)
Elevated CSF lactate, n = 298	44 (14.8)	63 (21.4)	44 (14.8)	62 (20.8)	193 (64.8)
Elevated CSF protein, n = 281	44 (15.7)	57 (20.3)	32 (11.4)	43 (15.3)	195 (69.4)
Decreased CSF glucose, n = 138	23 (16.7)	26 (18.8)	12 (8.7)	15 (10.9)	101 (73.2)
Decreased CSF:venous glucose ratio, n = 253	40 (15.8)	49 (19.4)	27 (10.7)	35 (13.8)	179 (70.8)
Elevated CSF white cell count, n = 341	44 (12.9)	64 (18.8)	39 (11.4)	57 (16.7)	241 (70.7)
Combinations, ≥1 of:					
Abnormal CSF lactate, protein, glucose, WCC, CSF turbid, n = 418	53 (12.7)	76 (18.2)	54 (12.9)	75 (17.9)	291 (69.6)
Elevated CRP, CSF lactate, protein, turbid, n = 427	56 (13.1)	82 (19.2)	59 (13.8)	83 (19.4)	289 (67.7)
Elevated CRP, CSF lactate, protein, n = 425	56 (13.2)	82 (19.3)	58 (13.6)	82 (19.3)	288 (67.8)
Elevated CRP, CSF lactate, n = 385	54 (14.0)	78 (20.3)	56 (14.5)	78 (20.3)	254 (66.0)
Elevated CRP, CSF protein, n = 382	54 (14.1)	75 (19.6)	49 (12.8)	68 (17.8)	261 (68.3)
Elevated CRP, GCS score<15, n = 348	50 (14.4)	72 (20.7)	49 (14.1)	70 (20.1)	229 (65.8)
Elevated CSF protein, GCS score <15, n = 348	49 (14.1)	68 (19.5)	44 (12.6)	61 (17.5)	239 (68.7)
GCS score <15, elevated CSF lactate, n = 361	48 (13.3)	69 (19.1)	50 (13.9)	70 (19.4)	244 (67.6)
GCS score <15, elevated CSF lactate, protein, n = 404	52 (12.9)	75 (18.6)	53 (13.1)	74 (18.3)	279 (69.1)

*GCS score <15 = GCS score total <15 and when GCS score total is missing = confused or disoriented CRP, C-reactive protein; CSF, cerebrospinal fluid; GCS, Glasgow coma scale; WCC, white cell count.
†Neck stiffness: history or examination.

Appendix Table 17. Criteria for definitions of encephalitis and meningitis as used in different published studies*

Reference	Study	Clinical syndrome	Definition
WHO 2003 guidelines (*56*)		Encephalitis	Acute onset of fever and $\geq$1 of: change in mental status (including confusion, disorientation, coma, or inability to talk, defined here as Glasgow Coma Score <15); new onset of seizures (excluding simple febrile seizures).
		Meningitis	A history of fever or documented fever (>38.5°C) and $\geq$1 of: neck stiffness, altered consciousness, or other meningeal signs.
Olsen et al. 2015 (*42*)	Prospective study in 7 hospitals in Thailand, 2003–2005	Enrolment	Acute brain dysfunction requiring hospitalization (altered mental status, focal central neurologic findings, or new onset of seizures), within 14 d or 7 d after admission and documented fever ($\geq$38°C) or history of fever or hypothermia ($\leq$35°C) and clinical indication for LP as determined by patient's physician
		Encephalitis	And $\geq$1 of: abnormal neuroimaging; abnormal EEG; CSF pleocytosis ($\geq$15 leukocytes/mm^3 for $\leq$6 weeks of age, $\geq$5 leukocytes/mm^3 for >6 weeks of age).
		Meningoencephalitis	Encephalitis with CSF pleocytosis and neck stiffness
Polage and Cohen 2016 (*57*)	Review on epidemiology and diagnosis for meningitis and encephalitis in developed countries	Encephalitis	Altered mental status and $\geq$2 of: fever; seizure; focal neurologic findings; CSF pleocytosis ($\geq$5 CSF leukocytes/mm^3); abnormal neuroimaging; abnormal EEG (refer to Venkatesan et al. 2013) (*58*).
		Meningitis	No clear definition. Patients with meningitis typically present with some combination of fever, headache, meningeal irritation, and altered mental status.
Tarantola et al. 2014 (*59*)	Review on burden of JEV in Mekong region	Acute encephalitis syndrome	Fever and $\geq$1 of (of sudden onset [<7 d]): altered mental status; motor deficit; sensory deficit; seizures of new onset (excluding simple febrile seizures).
		Meningoencephalitis	And meningism (nuchal rigidity)
Venkatsen et al. 2013 (*58*)	Consensus statement of the international Encephalitis consortium	Encephalitis and encephalopathy	Major Criterion (required): Patients presenting to medical attention with altered mental status (defined as decreased or altered level of consciousness, lethargy or personality change) lasting $\geq$24 h. And minor criteria (2 required for possible encephalitis; $\geq$3 required for probable or confirmed encephalitis): Documented fever $\geq$38°C (100.4°F) within the 72 h before or after presentation; generalized or partial seizures not fully attributable to a preexisting seizure disorder; new onset of focal neurologic findings; CSF leukocytes $\geq$5 leukocytes/mm^3; abnormality of brain parenchyma on neuroimaging suggestive of encephalitis that is either new from prior studies or appears acute in onset; abnormality on electroencephalography that is consistent with encephalitis and not attributable to another cause.
Glaser et al. 2003 (*60*), Glaser et al. 2006 (*61*)	Prospective study in California, 1998 to 2005	Encephalitis	Encephalopathy (depressed, or altered level of consciousness lasting $\geq$24 h, lethargy, or change in personality) and $\geq$1 of: fever; seizure; focal neurologic findings; CSF pleocytosis; electroencephalography; neuroimaging findings consistent with encephalitis.
Kolski et al. 1998 (*62*)	Prospective study at Toronto hospital, 1994–1995	Encephalitis	Depressed or altered level of consciousness $\geq$24 h and included lethargy, extreme irritability, or a significant change in personality or behavior and $\geq$2 of: fever; seizure; focal neurologic findings; >5 CSF WCC/µL; electroencephalogram findings compatible with encephalitis; abnormal results of neuroimaging.
Kupila et al. 2006 (*63*)	Prospective study at Finland hospital, 1999–2003	Aseptic meningitis	Symptoms or signs of meningeal inflammation, without evidence of brain parenchymal involvement and first CSF WCC >5 per µL and CSF bacterial culture negative.
		Encephalitis	$\geq$1 of altered consciousness or personality; epileptic seizures; focal neurologic signs and either >5 CSF WCC/µL; neuroradiological finding; EEG findings.
Mailles et al. 2009 (*64*)	National multicenter prospective study in France, 2007	Encephalitis	Acute onset of illness and $\geq$1 of: $\geq$4 CSF WCC/µL; CSF protein $\geq$40 mg/dL and fever and $\geq$1 of: decreased consciousness; seizure; altered mental status; focal neurologic signs.
Granerod et al. 2010 (*65*)	Prospective study in 24 hospitals in England, 2005 2006	Encephalitis	Altered consciousness $\geq$24 h and $\geq$2 of: fever; seizure; focal neurologic findings; $\geq$5 CSF WCC/µL; EEG findings; abnormal neuroimaging.
Ho Dang Trung et al. 2012 (*46*)	Prospective study in 13 hospitals in Vietnam, 2007 2010	Viral encephalitis and meningitis	Fever and $\geq$1 of: meningeal signs (neck stiffness, Kernig sign, Brudzinski sign); change in mental status; new onset of seizure. And $\geq$10 CSF WCC/µL (and 2 of: protein $\leq$1 g/L, normal glucose, lactate <4 mmol/L) or clear CSF (when <10 CSF WCC/µL)

Reference	Study	Clinical syndrome	Definition
		Bacterial meningitis	Fever and ≥1 of: meningeal signs (neck stiffness, Kernig sign, Brudzinski sign); altered consciousness and ≥10 CSF WCC/µL (and 2 of: protein >1 g/L, glucose <2.2 mmol/L, lactate ≥4 mmol/L) or turbid CSF (when <10 CSF WCC/µL).
Xie et al. 2015 (*44*)	Prospective study in 12 hospital in China, 2007–2012	Acute meningitis and encephalitis	≥1 of: fever; headache; vomiting And meningeal sign or change in mental status
Srey et al. 2002 (*52*)	Prospective study in 1 hospital in Cambodia, October 1999 September 2000	Encephalitis syndrome	Fever and ≥1 of: altered consciousness; focal neurologic sign.
Touch et al. 2009 (*51*)	JEV sentinel surveillance in children in 6 Cambodian hospital, 2006 2008	Meningoencephalitis	Fever and ≥1 of: neck stiffness; altered consciousness; another meningeal sign.
Han et al. 2016 (*53*)	Retrospective study in single hospital in Korea, March 2008 to Feb 2013	Aseptic meningitis	Fever with headache, meningeal irritation, and ≥5 CSF WCC/µL and normal CSF glucose and negative bacterial culture and not altered consciousness or seizure, or focal neurologic deficit.
Horwood et al. 2007 (*50*)	Prospective study from July 2010 to December 2013 at Kantha Bopha and Jayavarman VII, children hospitals in Phnom Penh and Siem Reap respectively	Acute meningoencephalitis	Fever >38°C, or febrile episode reported within the previous month. And CSF abnormalities (>4 WCC/µL or CSF protein >0.4g/L) and at least 1 of: confusion; prolonged, altered consciousness; seizure; central neurologic deficiency.

*In 2015, we reviewed articles published in English in the Medline database in the past 20 y, using the terms “encephalitis,” “meningitis,” “CNS syndrome” “CNS infection” “central nervous system syndrome” “central nervous system infection.” We selected article presenting prospective study of patients or review, where the criteria for definition of encephalitis and/or meningitis were clearly specified. CSF, cerebrospinal fluid; EEG, electroencephalogram; JEV, Japanese encephalitis virus; LP, lumbar puncture; WCC, white cell count; WHO, World Health Organization.

ppendix Table 18. List of primers and probes used for the detection or the typing of pathogens by PCR

Test	Gene	Oligo	5′→3′ sequence
Cryptococcus PCR for typing	*CAP59*	Forward primer	CCTTGCCGAAGTTCGAAACG
		Reverse primer	AATCGGTGGTTGGATTCAGTGT
Neisseria meningitidis serotyping Quadruplex qPCR (*22*)	CtrA	Forward primer	GCTGCGGTAGGTGGTTCAA
		Reverse primer 1	TTGTCGCGGATTTGCAACTA
		Reverse primer 2	TTGCCGCGGATTGGCCACCA
		Probe	6FAM-CATTGCCACGTGTCAGCTGCACAT
	SiaD$_B$	Forward primer	ATTATACAGCCTGCTCATCTCTATATGC
		Reverse primer	TCCCTTCATCAATTAAATGAGTCGTA
		Probe	6FAM-TTACAGGCCACTACTCCT-NFQ-MGB
	Ply	Forward primer	TGCAGAGCGTCCTTTGGTCTAT
		Reverse primer	CTCTTACTCGTGGTTTCCAACTTGA
		Probe	VIC-TGGCGCCCATAAGCAACACTCGAA
	Internal control	Forward primer	CCCTTGTCGAGCATTTAAAAGAG
		Reverse primer	TTCATGTATGGTTCATCCTCGAA
		Probe	Cy5-CATCGAGGCCAACTCGAAACATCGG-BHQ
Haemophilus influenzae typing, (*20*)	Hib *cap* locus	Forward primer	TGTTCGCCATAACTTCATCTTAGC
		Reverse primer	CTTACGCTTCTATCTCGGTGATTAATAA
		Probe	JOE-CACAAAACTTCTCATTCTTCGAGCCTA-BHQ1
	bexA	Forward primer	CTGAATTRGGYGATTATCTTTATGA
		Reverse primer	ACAATCAAAYTCAACHGAAAGHGA
		Probe	CY3-AGGGATGAAAGCYCGRCTTGCAT-BHQ2
	ompP2	Forward primer	GGTGCATTCGCAGCTTCAG
		Reverse primer	GATTGCGTAATGCACCGTGTT
		Probe	6FAM-TTGTTTATAACAACGAAGGGACTAACGT-BHQ1
Leptospira spp.	*rrs*	Forward primer	CCCGCGTCCGATTAG

Test	Gene	Oligo	5′→3′ sequence
		Reverse primer	TCCATTGTGGCCGRACAC
		Probe	6FAM-CTCACCAAGGCGACGATCGGTAGC-BHQ1
Niesseria meningitidis qPCR	*ctrA*	Forward primer	GCTGCGGTAGGTGGTTCAA
		Reverse primer	TTGTCGCGGATTTGCAACTA
		Probe	FAM-CATTGCCACGTGTCAGCTGCACAT-BHQ1
H. influenzae qPCR	bexA	Forward primer	GGCGAAATGGTGCTGGTAA
		Reverse primer	GGCCAAGAGATACTCATAGAACGTT
		Probe	HEX–CACCACTCATCAAACGAATGAGCGTGG-BHQ1
Streptococcus pneumoniae qPCR	*lytA*	Forward primer	ACGCAATCTAGCAGATGAAGCA
		Reverse primer	TCGTGCGTTTTAATTCCAGCT
		Probe	ROX-GCCGAAAACGCTTGATACAGGGAG-BHQ2
Streptococcus. suis qPCR	*cps2J*	Forward primer	GGTTACTTGCTACTTTTGATGGAAATT
		Reverse primer	CGCACCTCTTTTATCTCTTCCAA
		Probe	6FAM-TCAAGAATCTGAGCTGCAAAAGTGTCAAATTGA-TAMRA
S. pneumoniae typing qPCR	*Cps* serotype 1	Forward primer	CTATAGAAGGTCTACATCAGGTTC
		Reverse primer	TTTCTGTCAGATACGGCTTAC
		Probe	HEX-TCT[+T]CA[+A]TG[+C]GT[+A]GT[+C]TGC-BHQ1
	Cps serotype 3	Forward primer	ATGTTATTACACTCCTGTTCCTG
		Reverse primer	TCTAGGCGTCCATACTGTATC
		Probe	FAM-AGA[+A]CT[+G]TA[+A]TA[+T]CA[+C]TCTGCGA-BHQ1
	Cps serotype 4	Forward primer	TATTTCTAGGGTAATAACTGATTCTAAAAC
		Reverse primer	CTCCTAAATCATCTATTATTCCTGAAC
		Probe	Cy5-CTG[+C]CT[+C]TG[+A]AT[+A]TG[+C]TGAAT-BHQ2
	Cps serotype 5	Forward primer	TCCGAACGAAGATATTTGGTG
		Reverse primer	ATATAGAATTCCCCTCATGAACAC
		Probe	ROX-ACC[+A]CA[+A]CA[+T]CC[+T]CA[+A]TCAAC-BHQ2
	Cps serotype 6 A/B	Forward primer	TATTATTCTTTAGGGAATGTGTATACTG
		Reverse primer	ATATAACCACGCTGTAAAACTC
		Probe	HEX-CAA[+T]AC[+C]AA[+T]TA[+C]AC[+C]AAAGTCT-BHQ1
	Cps serotype 7 A/F	Forward primer	CCTTATAAATTTTGTGACTATAGACCTG
		Reverse primer	CCTAGTAAGACATCTGTGTCAC
		Probe	FAM-AAC[+C]CC[+A]GT[+A]AT[+C]AT[+A]ACCC-BHQ1
	Cps serotype 9 A/L/N/V	Forward primer	GTTAGTTGCTTCTTACAGGAAATAC
		Reverse primer	AAATTCATATTCCCACTCATTGTATG
		Probe	Cy5-ACT[+T]CC[+A]TC[+A]GT[+A]AG[+C]AGTTT-BHQ2
	Cps serotype 14	Forward primer	TCTATATACAAAGAGGCTCCAATG
		Reverse primer	ACCTGTATATCTTACACCATAACTAG
		Probe	ROX-AAA[+T]CC[+G]TC[+C]CA[+G]TC[+T]AAC-BHQ2
	Cps serotype 18 B/C	Forward primer	TCGATTTAGTAATCCCTGAAAC
		Reverse primer	GATAATCAAATTTACCTTTCCAATC
		Probe	HEX-TCA[+G]AT[+G]TT[+A]AA[+G]ACTACC-BHQ1
	Cps serotype 19 A	Forward primer	TGTTTGTTTTTGTGTCTGGTTTTTC
		Reverse primer	AGATGAGACGATTGTTAGCG
		Probe	ROX-TCT[+T]TG[+T]TG[+C]TC[+T]TT[+C]TT[+C]TTCT-BHQ2
	Cps serotype 19 F	Forward primer	TCGGACACTAGGAGTTACTG
		Reverse primer	AAAGCACCTACAGCAAAGAC
		Probe	FAM-ACA[+T]AC[+A]TA[+C]CA[+A]CT[+A]GA[+C]CAA-BHQ1
	Cps serotype 23	Forward primer	GAACGGTAGAGATGCCTTTAC
		Reverse primer	GAAGATATAAACTTAAACAGCACTATAATG
		Probe	Cy5-CAA[+C]TA[+A]CC[+C]AA[+C]AT[+A]AC[+C]ATTT-BHQ2
Orientia tsutsugamushi	47-kD	Forward primer	AACTGATTTTATTCAAACTAATGCTGCT
		Reverse primer	TATGCCTGAGTAAGATACRTGAATRGAATT
		Probe	6FAM-TGGGTAGCTTTGGTGGACCGATGTTTAATCT-TAMRA
Rickettsia spp.	*17kDa*	Forward primer	GGGCGGTATGAAYAAACAAG
		Reverse primer	CCTACACCTACTCCVACAAG
		Probe	6FAM-CCGAATTGAGAACCAAGTAATGC-TAMRA
R. typhi	*ompB*	Forward primer	TGGTATTACTGCTCAACAAGCT
		Reverse primer	CAGTAAAGTCTATTGATCCTACACC
		Probe	6FAM-CGCGATCGTTAATAGCAGCACCAGCATTATCGCG-BHQ1
Rickettsia sp. heminested PCR	*17kDa*	Forward primer 1	ACTTTACAAAATTCTAAAAACCATATACT
		Forward primer 2	GCTCTTGCAGCTTCTATGTTACA
		Reverse primer	CATTGTCCGTCAGGTTGGCG
Pan-dengue qPCR	3′NC	Forward primer	AGGACYAGAGGTTAGAGGAGA
		Reverse primer	CGYTCTGTGCCTGGAWTGAT
		Probe	6FAM-ACAGCATATTGACGCTGGGARAGACC-TAMRA
Dengue 1 qPCR	Capsid	Forward primer	ATACCYCCAACAGCAGGAATT
		Reverse primer	AGCATRAGGAGCATGGTCAC
		Probe	6FAM-TTGGCTAGATGGRGCTCATTCAAGAAGAAT-TAMRA
Dengue 2 qPCR	5′NC-capsid	Forward primer	TGGACCGACAAAGACAGATTCTT
		Reverse primer	CGYCCYTGCAGCATTCCAA
		Probe	6FAM-CGCGAGAGAAACCGCGTGTCRACTGT-TAMRA
Dengue 3 qPCR	Capsid	Forward primer	AAGACGGGAAAACCGTCTATCAA
		Reverse primer	TTGAGAATCTCTTCGCCAACTG
		Probe	6FAM-ATGCTGAAACGCGTGAGAAACCGTGT-TAMRA

Test	Gene	Oligo	5′→3′ sequence
Dengue 4 qPCR	Capsid	Forward primer	CCATCCCACCRACAGCAGG
		Reverse primer	CAAGATGTTCAGCATGCGGC
		Probe	6FAM-ATGGGGACAGTTRAAGAAAAAYAAGGCCAT-TAMRA
Pan-enterovirus qPCR	5′ NC	Forward primer	CCCCTGAATGCGGCTAATCC
		Reverse primer	ATTGTCACCATAAGCAGCCA
		Probe	6FAM-CANGGACACCCAAAGTAGTCGGTTCC-TAMRA†
Influenzavirus A SYBR Green RT-PCR or qPCR	Matrix	Forward primer	GGACTGCAGCGTAGACGCTT‡
		Reverse primer	CATYCTGTTGTATATGAGGCCCAT
		Probe	6FAM-CTCAGTTATTCTGCTGGTGCACTTGCCA-TAMRA
Influenzavirus B SYBR Green RT-PCR or qPCR	Hemagglutinin	Forward primer	AAATACGGTGGATTAAAYAAAAGCAA§
		Reverse primer	CCAGCAATAGCTCCGAAGAAA
		Probe	6FAM-CACCCATATTGGGCAATTTCCTATGGC-TAMRA
Panflavivirus SYBR Green RT-PCR	Nonstructural protein 5	Forward primer 1	TGYRTBTAYAACATGATGGG
		Forward primer 2	ATHTGGTWYATGTGGYTDGG
		Reverse primer	GTGTCCCAICCNGCNGTRTC⁋
HSV1 and HSV2 qPCR	pol	Forward primer	CATCACCGACCCGGAGAGGGAC
		Reverse primer	GGGCCAGGCGCTTGTTGGTGTA
		Probe	6FAM-CCGCCGAACTGAGCAGACACCCGCGC-TAMRA
HSV1 qPCR	Glycoprotein D	Forward primer	CGGCCGTGTGACACTATCG
		Reverse primer	CTCGTAAAATGGCCCCTCC
		Probe	6FAM-CCATACCGACCACACCGACGAACC-TAMRA
HSV2 qPCR	Glycoprotein G	Forward primer	CGCTCTCGTAAATGCTTCCCT
		Reverse primer	TCTACCCACAACAGACCCACG
		Probe	6FAM-CGCGGAGACATTCGAGTACCAGATCG-TAMRA
Varicella zoster virus qPCR	pol	Forward primer	GGTTAAACGTTTGAATCCATCC
		Reverse primer	CAGCAGACTTTCTCGAACGT
		Probe	6FAM-ATGCCACCTTTACAGTTGGAGGAA-TAMRA
West Nile virus qPCR	3′NC	Forward primer	CAGACCACGCTACGGCG
		Reverse primer	CTAGGGCCGCGTGGG
		Probe	6FAM-TCTGCGGAGAGTGCAGTCTGCGAT-TAMRA
T4 phage qPCR	rIIA	Forward primer	CCATCCATAGAGAAAATATCAGAACGA
		Reverse primer	CGCTGGGAAAAGAGGAATTATTTA#
		Probe	VIC-AACCAGTAATTTCATCTGCTTCTGATGTGAGGC-TAMRA
MS2 phage qPCR	Replicase	Forward primer	CTCTGAGAGCGGCTCTATTGGT
		Reverse primer	GTTCCCTACAACGAGCCTAAATTC
		Probe	VIC-TCAGACACGCGGTCCGCTATAACGA-TAMRA
Mumps virus qPCR	Fusion	Forward primer	TCTCACCCATAGCAGGGAGTTATAT
		Reverse primer	GTTAGACTTCGACAGTTTGCAACAA
		Probe	6FAM-AGGCGATTTGTAGCACTGGATGGAACA-TAMRA
Human cytomegalovirus qPCR	pp65	Forward primer	GCAGCCACGGGATCGTACT
		Reverse primer	GGCTTTTACCTCACACGAGCATT
		Probe	6FAM-CGCGAGACCGTGGAACTGCG-TAMRA
Measles virus qPCR	N3	Forward primer	TGGCATCTGAACTCGGTATCAC
		Reverse primer	TGTCCTCAGTAGTATGCATTGCAA
		Probe	6FAM-CCGAGGATGCAAGGCTTGTTTCAGA-TAMRA
Tick-borne encephalitis virus qPCR	3′NC	Forward primer	GGAMGRACMGATGAATACAT
		Reverse primer	GYGCYTCYTTCCAYTGCA[5]
		Probe	6FAM-CTCTGGACAGTGTGATGATGATGA-TAMRA
Henipahvirus qPCR	Nucleocapsid	Forward primer	TTCTTYGCRACYATCAGATT
		Reverse primer	ATTTCTCTGTAGAGYAGCATCA
		Probe	6FAM-TTCCAGAGTGAYCTCAAYACCATCAAA-TAMRA
Enterovirus reverse transcription for typing (*40*)	VP1	RT primer 1	GTYTGCCA
		RT primer 2	GAYTGCCA
		RT primer 3	CCRTCRTA
		RT primer 4	RCTYTGCCA
Enterovirus typing (*40*)	VP1	Forward primer 1	GCi-ATG-YTi-GGi-ACi-CAY-RT
		Reverse primer 1	CiC-CiG-GiG-GiA-YRW-ACA-T
	VP1	Forward primer 2	CCA-GCA-CTG-ACA-GCA-GYN-GAR-AYN-GG
		Reverse primer 2	TAC-TGG-ACC-ACC-TGG-NGG-NAY-RWA-CAT
Enterovirus sequencing (*40*)	VP1	Forward primer	CCA-GCA-CTG-ACA-GCA
		Reverse primer	TAC-TGG-ACC-ACC-TGG

*BHQ, black hole quencher; TAMRA, 6-carboxytetramethyl-rhodamine, Cy5, cyanine 5; HEX, hexachlorofluorescein; FAM, carboxyfluorescein; VIC, 2′-chloro-7′phenyl-1,4-dichloro-6-carboxy-fluorescein; ROX, 5- and 6-carboxy-X-rhodamin. Hib, *H. influenza* type b; HSV, herpes simplex virus; NC, noncoding; NS, nonstructural; PCR I, primary PCR; PCR II, secondary PCR; pol, polymerase; pp65, 65 kDa phosphoprotein; qPCR, quantitative PCR; RT-PCR, reverse transcriptase PCR; VP1, virus protein 1.

†The sequence has been slightly modified from the one published by Watkins-Riedel et al. (*32*). D was replaced by a N.

‡The sequence has been slightly modified from the one published by van Elden et al. (*38*). Second C was replaced by Y.

§The sequence has been slightly modified from the one published by van Elden et al. (*38*). Fifth T was replaced by Y.

⁋The sequence has been slightly modified from the one published by Moureau et al. (*34*). First D was replaced by I. The second and third D were replaced by N.

#The sequences published in original publications are wrong. They are the reverse complement of the right primers, in this table.

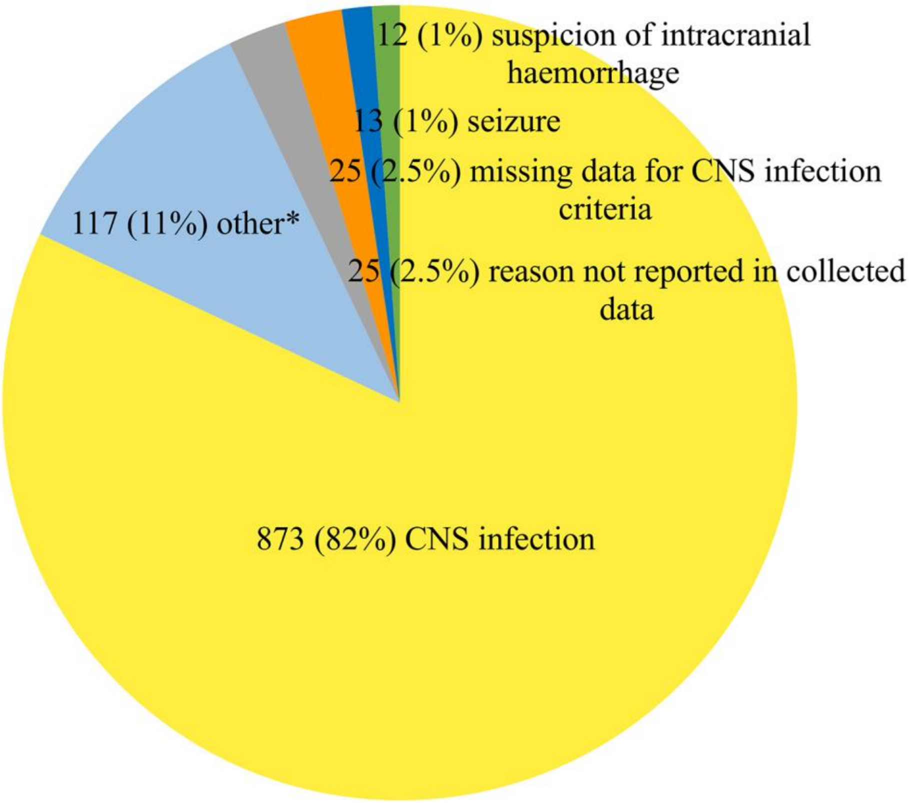

Appendix Figure 1. Distribution of indications for lumbar puncture. *Other reasons include headache, confusion, neck stiffness, beriberi, lupus, suspicion of Guillain Barré syndrome, hepatic encephalopathy, diabetes with coma. Lumbar puncture was unsuccessful and cerebrospinal fluid could not be collected for 40 (3.7%) patients. CNS, central nervous system.

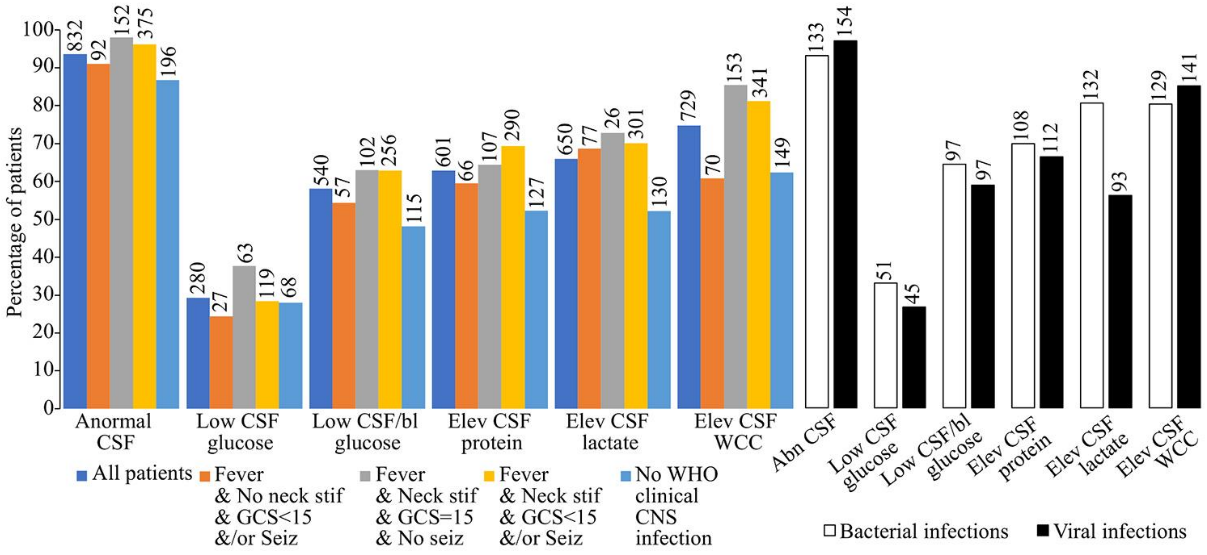

Appendix Figure 2. Percentage of patients with abnormal CSF parameters according to clinical presentations, viral and bacterial infections. No CNS infection indicates patients who don't meet criteria for WHO clinical CNS infection (fever with either GCS score <15, neck stiffness, or history of seizure). Patients with $\geq$1 of elevated CSF WCC, decreased glucose, elevated CSF protein, or elevated CSF lactate are presented on left of histograms as "abnormal CSF." Frequency of each criteria alone are also presented as well as decrease CSF: blood glucose. In total, 832 patients (93.6%) had abnormal CSF (elevated CSF WCC and/or low CSF glucose and/or elevated CSF lactate and/or elevated CSF protein), significantly more frequently in patients presenting with pure meningitis (98.1%, p = 0.026) and significantly less frequent in patients presenting without criteria for WHO clinical CNS infection (86.7%, p = 0.001). Two hundred eighty (29.3%) patients had low CSF glucose, significantly more frequent in patients presenting with pure meningitis (37.7% p = 0.030). Five hundred forty (58.1%) patients had low CSF/blood glucose ratio, significantly less frequent in patients presenting without criteria for clinical WHO clinical CNS infection (48.1%, p = 0.005). Six hundred and one (62.9%) patients had elevated CSF protein, significantly less frequent in patients presenting without criteria for WHO clinical CNS infection (52.3%, p = 0.003). Six hundred fifty (66%) patients had elevated CSF lactate, significantly less frequent in patients presenting without criteria for clinical WHO clinical CNS infection (52.2%, p<0.001) and in patients with viral infection (56%, p<0.001), and significantly more frequent in patients with bacterial infection (80.5%, p<0.001). Seven hundred twenty-nine (74.8%) patients had elevated CSF WCC, significantly less frequent in patients presenting with pure encephalitis (60.9%, p = 0.001) and in patients presenting without criteria for WHO clinical CNS infection (62.3%, p<0.001), and significantly more frequent in patients presenting with pure meningitis (85.5%, p = 0.002) and in patients presenting with meningoencephalitis (81.2%, p = 0.009). See Appendix Table 3 for reference ranges for laboratory variables. abn, abnormal; bl, blood; CSF, cerebrospinal fluid; elev, elevated; GCS, Glasgow coma scale; seiz, seizure; stif, stiffness; WCC, white cell count; WHO, World Health Organization.
